# Genetic and geographical insights call for early conservation of Mae Hong Son’s blue mahseer to prevent population crisis

**DOI:** 10.1371/journal.pone.0313505

**Published:** 2025-02-12

**Authors:** Tavun Pongsanarm, Thitipong Panthum, Trifan Budi, Wongsathit Wongloet, Aingorn Chaiyes, Chadaphon Thatukan, Wattanawan Jaito, Chananya Patta, Worapong Singchat, Prateep Duengkae, Narongrit Muangmai, Kiatisak Wangwon, Kornsorn Srikulnath

**Affiliations:** 1 Animal Genomics and Bioresource Research Unit (AGB Research Unit), Faculty of Science, Kasetsart University, Chatuchak, Bangkok, Thailand; 2 Laboratory of Animal Cytogenetics and Comparative Genomics (ACCG), Department of Genetics, Faculty of Science, Kasetsart University, Chatuchak, Bangkok, Thailand; 3 Special Research Unit for Wildlife Genomics (SRUWG), Department of Forest Biology, Faculty of Forest, Kasetsart University, Chatuchak, Bangkok, Thailand; 4 Interdisciplinary Graduate Program in Bioscience, Faculty of Science, Kasetsart University, Chatuchak, Bangkok, Thailand; 5 School of Agriculture and Cooperatives, Sukhothai Thammathirat Open University, Nonthaburi, Thailand; 6 Sciences for Industry, Faculty of Science, Kasetsart University, Chatuchak, Bangkok, Thailand; 7 Department of Fishery Biology, Faculty of Fisheries, Kasetsart University, Chatuchak, Bangkok, Thailand; 8 Tham Pla—Namtok Pha Suea National Park, Huai Pha, Mueang Mae Hong Son, Mae Hong Son, Thailand; 9 Biodiversity Center Kasetsart University (BDCKU), Chatuchak, Bangkok, Thailand; The Islamia University of Bahawalpur Pakistan, PAKISTAN

## Abstract

Ecosystems are being disrupted by climate change and habitat fragmentation, which affect species survival through altered mating, feeding, and migration patterns. Mae Hong Son Province, Thailand, harbors a unique hydrological network that supports rich freshwater fish biodiversity. Blue mahseer (*Neolissochilus stracheyi*), which is restricted to headwater streams in Mae Hong Son, is particularly sensitive to habitat disturbances and has experienced population decline. Despite their vulnerability to climate change and habitat fragmentation, information on the genetic diversity, population structure, and environmental drivers of their distribution remains limited. In this study, microsatellite genotyping and mitochondrial DNA displacement loop sequence analysis were used to assess the genetic diversity and population structure of five blue mahseer populations in Mae Hong Son, with the aim of identifying reliable conservation units for effective management. Low genetic diversity levels across populations were identified (expected heterozygosity = 0.452 ± 0.037; allelic richness = 3.150 ± 0.506) with no evidence of inbreeding or outbreeding. A forecasted drop in heterozygosity below 0.1 within 50 years indicated the urgency of conservation attention. The five blue mahseer populations were clustered into three evolutionarily significant units (ESUs) based on historical isolation, phylogenetic distinctness, and significant genetic differentiation. Habitat suitability was assessed using MaxEnt species distribution modeling, which identified distance to rivers and annual mean total precipitation as significant environmental variables. The correlation between genetic differentiation and geographical distance suggested that habitat conditions primarily influence population genetic structure. Stocking between ESUs with differing genetic stocks is discouraged to avoid negative genetic effects. A comprehensive understanding of blue mahseer population dynamics, informed by the integration of genetic and ecological data, is needed to inform conservation strategies for resource management in Mae Hong Son.

## Introduction

Climate change causes large-scale alterations in water quality and temperature, thereby affecting species survival. These changes, including shifts in seasonal patterns and water cleanliness, directly affect breeding, feeding, and migration. Such environmental shifts are critical for species that rely on specific habitat conditions and render them vulnerable to rapid climate-induced changes [1, 2]. Mae Hong Son Province, Thailand (97° 20’–98° 39’ E, 17° 38’–19° 48’ N), consists primarily of highlands and complex mountain ranges with rainforests, covering an area of 12 780 km^2^ and elevations of 26–2005 m [3]. Mae Hong Son, known for its abundant freshwater resources and minimal urbanization, features various drainage systems comprising small rivers flowing into the Salween River and the Andaman Sea, making it a biodiversity hotspot for freshwater fish [4]. Most of Mae Hong Son’s land area, recognized for its high biodiversity, is under protection by the Department of National Parks, Wildlife, and Plant Conservation (DNP) of the Thai government [5]. The protected areas, primarily forested mountains with headwater streams, are central to research focusing on species diversity and endemism. However, the occurrence of common freshwater fish species, such as mahseer (Cyprinidae), particularly in pristine environments such as caves and natural canals in Mae Hong Son, is frequently underrepresented.

**Fig 1:**
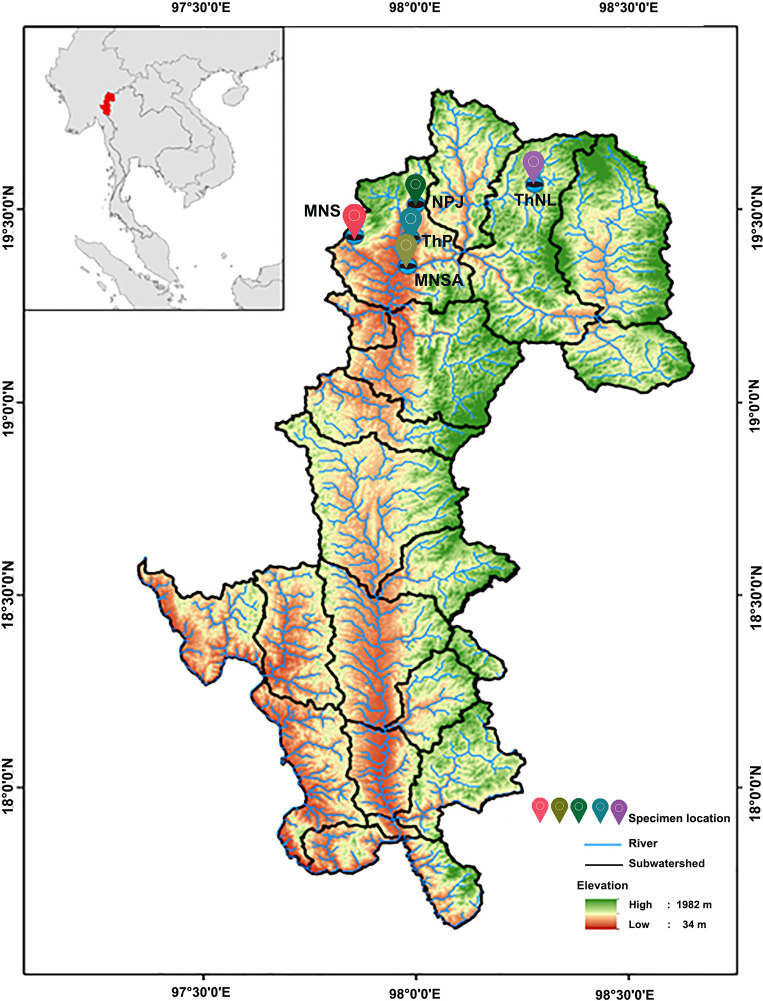
Map showing survey locations of blue mahseer (*Neolissochilus stracheyi*) in Mae Hong Son Province, North Thailand. Province boundary and river network data were obtained from the Land Development Department (http://sql.ldd.go.th/ldddata/mapsoilB1.html) generated using QGIS version 3.34.8.

Mahseers, a valuable group of freshwater fish in Asia, belong to the *Tor*, *Neolissochilus*, and *Naziritor* genera of the Cyprinidae family [6]. These fish, commonly found in Southeast Asian rivers and streams, are of ecological and economic significance [7, 8]. Blue mahseer (*Neolissochilus stracheyi*), is distributed along the rivers and headwater streams between the Myanmar–Thailand boundary, including Mae Hong Son, and reach a maximum length of 60.0 cm [9]. These waters are part of two sub-watersheds: the Lower Mae Pai (Part 1) and Lower Mae Pai (Part 2) (Fig 1) [10]. This species is typically found in the clear upstream waters of fast-flowing mountain and hill streams with rocky bottoms in the Dan Lao Range, as well as in clear forest streams, and is sensitive to habitat disturbance [7]. They are known to migrate long distances (>120 km) to feed and spawn during monsoons, exhibiting potamodromous homing behavior [11]. Given the species’ typical restriction to headwater streams and its absence downstream, differentiation among populations at various sites may be expected, as previously observed in the Soi River (Mai Sapa), Sa-at River (Mae Surin Waterfall National Park), Sanghi River (Nong Pla Jat), Tham Pa (Pa Cave), and Tham Nam Lot (Nam Lot Cave) in Mae Hong Son. (Kiatisak Wangwon, personal communication). Blue mahseers, also known as large-scaled barbels, are characterized by bronze backs, silver abdomens, and a black lateral stripe [6, 7]. This species is a popular tourist attraction because of its abundance, with tourists often watching and feeding on the fish. However, natural populations of blue mahseers have sharply declined because of habitat degradation, environmental pollution, and illegal fishing, even in protected core habitats [12, 13]. The impact of climate change on water quality and ecosystem dynamics has greatly affected blue mahseers in Mae Hong Son’s headwater streams and caves. The breeding and growth of blue mahseer may be disrupted by temperature increases and altered precipitation, which alter water flow and pollutant runoff and leading to disrupt the lifecycle [14]. Urgent monitoring, adaptive management, and conservation strategies are necessary to address the threats to blue mahseer populations.

Species management and conservation are greatly informed by studies on genetic diversity. The characterization of populations and species within conservation units (CUs) is based on genetic divergence and gene flow rates. More than 20 species and subspecies of the genus *Neolissochilus* have been recognized, but their taxonomy, systematics, and uniform identification remain unclear [15]. Accurate species identification units are critical in conservation management because they inform the designation of CUs, evolutionarily significant units (ESUs), and management units (MUs) [16]. To fill this research gap in free-living blue mahseer, a study was conducted using rapid DNA barcoding to accurately reflect barcode efficiency in mahseer species, with the ultimate aim of enhancing the error detection capability and improve data quality. The CU, ESU, and MU of Mae Hong Son’s inland freshwater ecosystem were assessed based on the genetic diversity and population structure of populations in five locations using the displacement loop (D-loop) region of mitochondrial DNA (mtDNA D-loop) and microsatellite genotyping. In addition, species distribution within habitats is critically assessed in conservation biology, focusing on survival in interconnected populations and dependence on crucial environmental characteristics. Maximum entropy (MaxEnt) [17] now used in habitat suitability models, enables the ecological evaluation of niche functions for wildlife, such as blue mahseer, by converting them into interpretable factors for resource management [18]. Investigating the relationships between blue mahseers and their preferred habitats enables the prediction of the effects of habitat change on population management. In light of these scenarios, the following hypotheses were proposed: (i) central highland freshwater habitats in Mae Hong Son contain at least one CU of blue mahseer, possibly several ESUs or MUs, with variation between sub-watersheds, and (ii) there is little gene flow between sub-watersheds. This study found significant genetic variation in blue mahseer populations with a strong genetic–geographical correlation. The identification of diverse healthy populations is crucial for effective conservation.

## Materials and methods

### Obtaining partial mtDNA sequences of blue mahseer

Core nucleotide databases of public repositories were searched for mahseer (of the genera *Tor*, *Neolissochilus*, and *Naziritor*) mitochondrial cytochrome c oxidase I (*COI*) and cytochrome b (*Cytb*) sequences, that are frequently used for species identification and phylogenetics in teleosts [19–22]. In November 2023, 758 *COI* and 701 *Cytb* sequences were obtained from public repositories and aligned using Geneious Prime (version 2023.2, https://www.geneious.com). Large sequence length variations of 287–701 bp for *COI* and 594–1181 bp for *Cytb* were observed. The most common sequence lengths (590 bp for *COI* and 421 bp for *Cytb*) were selected for further analysis. Only sequence lengths with no missing nucleotides or gaps were included to reduce the possibility of nuclear mtDNA contamination [23]. The selected *COI* and *Cytb* coding sequences were aligned and translated using Molecular Evolutionary Genetics Analysis (MEGA) version 11 to verify the presence of an open reading frame without stop codons [24].

### Barcode validation and species delimitation

Sequence properties and suitability for species identification were evaluated for both *COI* and *Cytb* datasets. Alignment length, variable positions, and GC content were compared using the "ape" package in R [25]. Substitution saturation was assessed using S-V plots against Kimura’s-two-parameter (K2P) sequence divergence and information entropy-based index (I_ss_)/critical I_ss_ value (I_ss.c_) comparisons in DAMBE [26, 27]. Nucleotide substitutions at various codon positions were also examined. Sequence divergence based on the K2P model [28] was used to evaluate both distance and tree-based identification approaches. The nearest-neighbor test [29] was used to assess intraspecific relationships and the presence of a "barcoding gap" between intra- and interspecific divergences. Markers with high discriminatory power were identified based on correct nearest neighbor identification rates and positive barcoding gaps calculated using the "spider" package [30]. Kruskal–Wallis and Dunn’s tests were used to compare the barcoding gaps between *COI* and *Cytb* datasets. Phylogenetic analyses were conducted using MrBayes [31] along with Bayesian inference on rooted trees for the outgroups *Barbus chelynoides* (*Puntius chelynoides*) and Jullien’s golden carp (*Probarbus jullieni*). Monophyly and tree-based barcoding efficiency were evaluated using "spider," considering the limitations of small datasets [30, 32]. Species delimitation within mahseers was assessed separately for *Tor*, *Neolissochilus*, and *Naziritor* using the Bayesian Poisson Tree Process (bPTP) and Generalized Mixed Yule Coalescent (GMYC) methods on all datasets [32–34]. The bPTP web server was used with the maximum likelihood tree as input. GMYC analyses employed an ultrametric tree constructed with the "chronos" function in R [35].

### Specimen collection and DNA extraction

A total of 93 blue mahseer specimens were collected from five localities in Mae Hong Son province, North Thailand: 1) MNS: Soi River (Mai Sapa); 2) MNSA: Sa-at River (Mae Surin Waterfall National Park); 3) NPJ: Sanghi River (Nong Pla Jat); 4) ThP: Tham Pa (Pa cave); and 5) ThNL: Mae Lang River (Tham Nam Lot, Nam Lot cave) (Fig 1 and S1 Table in S1 File). This study was conducted under the authority of the Department of National Parks (DNP), Wildlife and Plant Conservation, and the Ministry of Natural Resources and Environment, Thailand (02-012-20092566). All animal care and experimental procedures were approved by the Animal Experiment Committee of the Kasetsart University (approval no. ACKU66-SCI-009) and conducted in accordance with the regulations for animal experiments at Kasetsart University and ARRIVE guidelines (https://arriveguidelines.org). Approximately 0.3 × 0.3 cm of the caudal fin from each live individual was collected and preserved in 95% ethanol, stored at 4°C, and subjected to genomic DNA extraction using the standard salting-out protocol [36]. Individual fish were released into the wild immediately after caudal fin collection. DNA quality and concentration were assessed using 1% agarose gel electrophoresis and spectrophotometry (NanoDrop One Microvolume UV-Vis Spectrophotometer; Thermo Fisher Scientific, Cleveland, OH, USA).

### Species identification using barcoding gap of mitochondrial *COI* and *Cytb* genes

Fifteen DNA samples (from three individuals per population) from 93 blue mahseer extractions were randomly selected to test the barcoding gap of mtDNA *COI* and *Cytb* genes using polymerase chain reaction (PCR) amplification. Partial fragments of the mtDNA *COI* gene were amplified using the primer pair Fish Primer 1_F1 (5′-TCAACCAACCAC AAAGACATTGGGAC-3′) and Fish Primer 2_R1 (5′-TAGACTTCTGGGTGGCCAAAGAATCA-3′) [37], while the primer pair L15138 (5′-ATGATGACCGCCTT CGTGGGCTA-3′) and H15560 (5′-GCGTAGGCAAATAGGAAGTATC-3′) were used to amplify partial fragments of the mtDNA *Cytb* gene [38]. PCR amplification was performed in a 15-μL reaction volume containing 1× buffer, 1.5 mM MgCl_2_, 0.2 mM dNTPs, 0.5 μM primers, 0.5 U of *Taq* polymerase (Apsalagen, Bangkok, Thailand), and 25 ng of genomic DNA. The PCR conditions were as follows: initial denaturation 94°C for 3 min, followed by 35 cycles of 94°C for 30 s, 52–58°C for 30 s, 72°C for 30 s, and a final extension at 72°C for 5 min. The PCR product sizes were confirmed using 1.5% agarose gel electrophoresis. The PCR products were purified using a GenUP PCR Cleanup Kit (Biotechrabbit, Hennigsdorf, Germany). The nucleotide sequences of the DNA fragments were determined using the DNA sequencing service of First Base Laboratories Sdn Bhd (Seri Kembangan, Selangor, Malaysia). The BLASTn and BLASTx programs (http://blast.ncbi.nlm.nih.gov/Blast.cgi, accessed on November 8, 2023) were used to search for nucleotide sequences in the National Center for Biotechnology Information database to confirm the identity of the amplified DNA fragments. The generated sequences have been deposited in the DNA Data Bank of Japan (DDBJ, S5–S6 Tables in S1 File). All nucleotide sequences were used for multiple sequence alignment with a set of nucleotide sequences derived from public repositories to validate the accipitrid DNA barcodes using the default parameters of MEGA version 11 [25].

### Microsatellite genotyping and data analysis

Thirteen microsatellite primer pairs were selected [39]. The 5′-end of the forward primer in each pair was labeled with a fluorescent dye (FAM, HEX, and TAMRA; Macrogen, Seoul, Korea). PCR amplification was performed in a 15-μL reaction volume of 1× Apsalagen buffer containing 2 mM MgCl_2_, 0.2 mM dNTPs, 0.5 μM primers, 0.5 U *Taq* polymerase (Apsalagen), and 25 ng genomic DNA. The PCR protocol was as follows: initial denaturation at 94°C for 3 min, followed by 35 cycles of 94°C for 30 s, 55°C for 30 s, and 72°C for 30 s, with a final extension at 72°C for 10 min. The PCR products were detected by 1% agarose gel electrophoresis. To decrease the influence of false alleles, PCR amplification was performed at least thrice for each sample. Fluorescent DNA fragment length analysis was performed using an ABI 3730XL automatic sequencer (Applied Biosystems, Foster City, CA, USA) at the DNA Genotyping Service of Macrogen. Allelic size was determined using Peak Scanner software (version 1.0; Applied Biosystems). The analysis of genetic diversity considered allelic frequency, number of alleles (*N*_a_), allelic richness (*AR*), number of effective alleles (*N*_ea_), Shannon’s information index (*I*), observed heterozygosity (*H*_o_), expected heterozygosity (*H*_e_), polymorphic information content (*PIC*), fixation index (*F*), Hardy–Weinberg equilibrium (HWE), Welch’s *t*-test (for significant differences between the *H*_o_ and *H*_e_ tests), linkage disequilibrium, relatedness (*r*), inbreeding coefficient (*F*_IS_), Wright’s F-statistic for subpopulations within the total population (*F*_ST_), principal coordinate analysis (PCoA), discriminant analysis of principal components (DAPC), null alleles, STRUCTURE analysis, and genetic selective sweep analysis based on microsatellite genotyping data, which was performed as previously described [40]. Forward population genetic and demographic scenarios were simulated for each population using individual-based genotype data in quantiNEMO version2 [41] as outlined by [3], with slight modifications. Four scenarios, each with a varied population carrying capacity, were simulated: a decrease in population size by –0.5; fixed current population size of 93; and increasing population size fixed to 46, 93, 139, and 186 individuals. The model parameters assumed a random mating system [39]. For each scenario, 1,000 replications were performed across 400 generations, with an assumed evolution rate of 3.61 × 10^6^/million years ago [42].

### Demography and landscape analyses

To investigate the population dynamics of blue mahseer, the probability of a bottleneck event was determined [43]. Recent gene flow among the populations was determined using BayesAss version 3.0.5 [44], historical gene flow among the populations were assessed using MIGRATE-N software version 3.6.11 [45]. Recent and historical gene flow dynamics were visualized using Circos version 0.69–8 [46]. Ancestral population admixture and phylogenetic relationships were evaluated in TreeMix software version 1.12 [47], isolation by distance (IBD) was assessed in IBD version 1.52 [48], and landscape shape interpolation (LSI) was assessed based on alleles in space (AIS) visualizations of the spatial patterns of genetic diversity [49]. All parameters and settings were followed [43]. The genetic variation values (*H*_e_, *AR*, *F*_IS_, and gene flow) of the five georeferenced localities were mapped onto the modelled distribution of blue mahseers by projecting the values using the inverse distance weighting spatial interpolation method (Watson, 1992) in Quantum GIS 3.34.8 [50].

### Mitochondrial DNA D-loop sequencing and data analysis

The mtDNA D-loop fragments were amplified using the in-house primer pair Tor_D-loop_F (5′-TAACCATAAAGCAAGTACTAAYTTTTAAGGTA-3′) and Tor_D-loop_R (5′-TTGRCAWGGATAACAGGATTTGYTGAGCGTA-3′). PCR amplification was performed in a 15-μL reaction volume containing 1× standard reaction buffer, 2.0 mM MgCl_2_, 0.2 mM dNTPs, 0.5 μM primers, 0.5 U Taq polymerase (Apsalagen), and 50 ng genomic DNA. The PCR conditions were as follows: initial denaturation at 94°C for 3 min, followed by 37 cycles of 94°C for 30 s, 52°C for 30 s, 72°C for 40 s, and a final extension at 72°C for 5 min. The PCR products were purified using the GenUP PCR Cleanup Kit (Biotechrabbit). The nucleotide sequences of the DNA fragments were determined using the DNA sequencing service of First Base Laboratories Sdn Bhd (Seri Kembangan, Selangor, Malaysia). All sequences were deposited in the DDBJ (https://www.ddbj.nig.ac.jp/, accessed on November 8, 2023; accession numbers LC785336–LC785346; S7 Table in S1 File).

The mtDNA D-loop sequence analysis considered haplotype diversity (*h*); nucleotide diversity (*π*) diversity; population differentiation based on *G*_ST_, *F*_ST_, and *Ф*_ST_ values; haplotype network; gene flow (*N*_m_) was conducted as previously described [40]. The *Tor* sequences available in the NCBI database (accession numbers: HQ625378 and HQ625381) were retrieved and used as outgroups in the phylogenetic tree. Migration rate, neutrality based on neutrality (Tajima’s D*, Fu and Li’s D* and F* tests, and Fu’s *F*s), raggedness index, mismatch distribution, and population expansion analyses were performed as previously described [40]. To evaluate historical demographic fluctuations, an extended Bayesian skyline plot (EBSP) was constructed [51].

### Study area, occurrence, and environmental data

A recent assessment found that 83% of Mae Hong Son (Fig 1) province’s area is forest, 14% is agricultural, 1% is urban, and only 0.2% is water bodies [52]. The distribution of blue mahseer in Mae Hong Son was predicted using 10 environmental layers, considering known limiting factors. These layers include the annual mean temperature, annual total precipitation (1981–2010), elevation, slope, aspect, flow accumulation, flow direction, proximity to rivers, normalized difference water index (NDWI), and normalized difference vegetation index (NDVI). Data on the annual mean temperature (°C) and total precipitation (mm) for 1981–2010 were obtained from meteorological stations of the Thailand Meteorological Department and then converted to raster format using inverse distance weighting. Elevation data (m) and river information for Thailand were acquired from the National Parks, Wildlife and Plant Conservation Department. The Euclidean distance to the main river (m) was calculated to determine the distance to water. The slope (degree), aspect, flow direction, and flow accumulation were derived using GIS version 3.34.8 software. Data for NDVI and NDWI were derived from Landsat 8 satellite images with a 30-m spatial resolution, specifically from five tiles of original images for January–April 2023 [53], and converted to raster format.

### Species distribution modeling

Distribution models were created using MaxEnt (beta version 3.4.4), a species distribution modeling program that employs a maximum entropy algorithm for predictions based on presence-only data [54]. MaxEnt is recognized for its superior performance among various modeling methods [55]. and its effectiveness with small sample sizes [56–58]. The logistic output was selected, featuring suitability data of 0–1 representing the occurrence probability of the target species. Default settings were used for the convergence threshold and maximum number of iterations, which were set to 500 [59]. Regularization data were selected automatically using MaxEnt software to reduce model overfitting [57]. The optimal MaxEnt model was selected based on the 10th percentile presence probability, and a five-fold cross-validation method was used to generate a binary map. Response curves of the predictor variables were computed and jackknife importance was tested in the final optimal model [60]. The predictive uncertainty was reduced using the ensemble forecasting approach [61]. Final logistic outputs were calculated using the basic mathematical function of mean ensembles [62].

## Results

### Datasets and sequence database evaluation

Using specific keyword terms such as “COI, COX1, MTCO1, Co I, CO1, cytochrome c oxidase I, CYTB, MTCYB, MT-CYB, cytochrome b, D-loop,” and Cyprinidae filters on Mitochondrion (dated November 20, 2023), a total of 1,483 nucleotide barcoding sequences (comprising 758 *COI* sequences, 701 *Cytb* sequences, and 24 D-loop sequences) were identified. The effectiveness of DNA barcoding in identifying erroneous sequences within public repositories was assessed using a compiled dataset of *COI* and *Cytb* sequences from the master group. Variations were observed in the lengths of the available *COI*, *Cytb*, and mtDNA D-loop sequences, presenting a trade-off between maximizing the alignment length and taxonomic coverage. Parameters based on the number and length of the sequences were applied for filtering, resulting in a final dataset consisting of 1,168 total sequences, including 659 *COI* sequences (590 bp) and 509 *Cytb* sequences (421 bp). Data on the mtDNA D-loop sequences were excluded from further analysis because of the low sample size and very short fragment sequences. In the final database, no sequences contained stop codons or frameshift mutations. Sequence alignments were 590 bp for *COI* and 421 bp for *Cytb*. For *COI* and *Cytb*, the number of variable sites was 392 and 77, respectively, and the GC contents were 43.59% for *COI* and 44.71% for *Cytb*. In the *COI* datasets, no intraspecific sequence divergence was observed among the 14 species. The minimum and maximum interspecific sequence divergence values for each species were 0.005‒0.076 (S3 Table in S1 File). No intraspecific sequence divergence was found among the 12 *Cytb* species. In 13 species, the minimum interspecific sequence divergence was 0.000–0.068 and the maximum interspecific sequence divergence was 0.003–0.068 (S3 Table in S1 File). The barcoding gap value was 0.00 for both the *COI* and *Cytb* datasets, indicating that DNA barcoding might be ineffective in distinguishing between closely related species with low genetic variation.

Sequence-based evaluation was conducted on the final dataset, and sequences from the same species with both intraspecific and distinct interspecific clustering with a probability of 0.90–1.00 were classified as “Class 1,” with a total of 539 *COI* (81.66%) and 434 *Cytb* sequences (85.26%). A total of 117 *COI* (17.72%) and 69 *Cytb* sequences (13.55%) were assigned as “Class 2,” comprising sequences of the same species without intraspecific clustering. “Class 3” consisted of sequences from different species, including four *COI* (0.60%) and 6 *Cytb* sequences (1.17%). For the tree-based evaluation, the *COI* and *Cytb* sequences were classified into two groups based on their position in the phylogenetic tree. Group 1 comprised sequences from closely related species clustered in a single clade containing 599 *COI* (90.75%) and 429 *Cytb* sequences (84.28%). Group 2 consisted of sequences from distinct species that were unexpectedly grouped in a single clade and contained 61 *COI* (9. 24%) and 80 *Cytb* sequences (15.71%). No sequences were classified as Group 3, which had no similarity to most sequences with the same species name [63]. Phylogenetic analyses based on *COI* and *Cytb* sequences strongly supported the 660 *COI* and 509 *Cytb* sequences, which belonged to the monophyletic group of each master species, with higher posterior probability. The phylogenetic tree of the *Cytb* and *COI* datasets had 47% and 41% of the monophyletic groups, respectively (S1C Fig). Substitution saturation was observed for both the *COI* and *Cytb* datasets (S2 Fig). The number of mutations (transitions and transversions) was nearly equal between the *COI* and *Cytb* datasets. A linear correlation was observed between the number of transitions and transversions plotted against sequences in the *COI* dataset (S3 Fig). The I_ss_ was lower than the I_ss.c_ in both the *COI* and *Cytb* datasets (S4 Table in S1 File), suggesting the absence of substitution saturation in both the *COI* and *Cytb* sequences [64]. Both *COI* and *Cytb* are appropriate markers for mahseer species identification; however, *Cytb* can give higher certainty (S1C Fig). Species delimitation, determined for the *COI* and *Cytb* datasets using GMYC and bPTP, supported 14 species based on the *COI* dataset and 12 species based on the *Cytb* dataset, and no tentative cryptic species were identified (S4‒S7 Figs).

### *COI* and *Cytb* sequence analysis

The amplicon lengths of *COI* and *Cytb* were 572–700 bp and 410–620 bp, respectively, while the alignment lengths were 590 and 421 bp, respectively. *COI* and *Cytb* sequences were aligned with consensus sequences from public repositories used in Section 3.1 to confirm their similarity within the expected range. All validated sequences were correctly identified at the species level based on phylogenetic tree and barcoding gaps, with 100% accuracy for *N*. *stracheyi* (S8 and S9 Figs).

### Genetic variability of blue mahseer (*Neolissochilus stracheyi*) population based on microsatellite data

From the total of 93 blue mahseer from five populations that were genotyped, 102 alleles were found across all 13 loci, with the mean number of alleles per locus being 3.815 ± 0.328 (Table 1). All allelic frequencies in the captive population significantly deviated from that expected under the Hardy–Weinberg equilibrium, indicating the presence of linkage disequilibrium(https://datadryad.org/stash/share/h8uyRkLq7XVtcnFjKuUSQv8FyyfPH7sgZBBcrGG4HBA). Departures from Hardy–Weinberg equilibrium in most microsatellite loci are commonly observed in natural fish populations across various species [65–67]. Null alleles were frequently observed in NY05, BS04, NY07, NY11, NY14, and NY06, and all the markers were treated similarly. The *PIC* of all blue mahseer populations was 0.369–0.439, and *I* was 0.741–0.985 (Table 1). The *H*_o_ values were 0.438–0.472 [mean ± standard error (SE): 0.455 ± 0.049] and the *H*_e_ values were 0.416–0.478 (0.452 ± 0.037; Table 1 and S11 Table in S1 File). Welch’s *t*-test showed that *H*_o_ did not differ from *H*_e_ in any population (S9 Table in S1 File), consistent with the pairwise *H*_o_ and *H*_e_ values between populations (S8 Table in S1 File). The *AR* value was 3.150 ± 0.506. The standard genetic diversity indices are summarized in Table 1 and S8 Table in S1 File. Four scenarios with varied carrying capacities were genetically simulated to estimate the loss of genetic diversity across populations, represented by *H*_e_ and *AR*. A decrease in genetic diversity was observed in 400 simulated generations. When blue mahseer populations have a relatively large carrying capacity, the decline in genetic diversity is relatively slow (S10 Fig).

**Table 1:** Genetic diversity of 93 blue mahseer (*Neolissochilus stracheyi*) individuals based on 13 microsatellite loci (detailed information for all individuals is presented in S1 Table in S1 File).

Population		N	*N*_a_	*AR*	*N*_ea_	*I*	*H*_o_	*H*_e_	*M* ratio	*PIC*	*F*
MNS ^1^	Mean	31	3.615	2.870	2.415	0.827	0.461	0.443	0.174	0.399	-0.028
	SE	0	0.646	1.491	0.381	0.170	0.108	0.085	0.068	0.286	0.183
MNSA ^2^	Mean	19	3.692	2.982	2.304	0.839	0.438	0.451	0.200	0.400	0.022
	SE	0	0.683	1.587	0.344	0.162	0.110	0.077	0.066	0.261	0.185
NPJ ^3^	Mean	19	4.615	3.606	3.170	0.985	0.443	0.471	0.215	0.438	0.038
	SE	0	1.003	2.359	0.650	0.229	0.116	0.098	0.096	0.342	0.183
ThP ^4^	Mean	12	4.077	3.536	3.088	0.958	0.472	0.478	0.193	0.439	-0.082
	SE	0	0.858	2.343	0.594	0.220	0.113	0.098	0.074	0.341	0.168
ThNL ^5^	Mean	12	3.077	2.758	2.006	0.741	0.464	0.416	0.228	0.369	0.007
	SE	0	0.366	1.067	0.211	0.125	0.116	0.070	0.039	0.223	0.183
All Population	Mean	93	3.815	3.150	2.596	0.870	0.455	0.452	0.200	0.409	-0.007
	SE	0	0.328	0.506	0.209	0.081	0.049	0.037	0.032	0.286	0.078

Sample size (N); number of alleles (*N*_a_); allelic richness (*AR*); number of effective alleles (*N*_ea_); Shannon’s information index (*I*); observed heterozygosity (*H*_o_); expected heterozygosity (*H*_e_); *M*-ratio test (*M* ratio); polymorphic information content (*PIC*); fixation index (*F*).

^1^ MNS = Mae Nam Soi.

^2^ MNSA = Mae Nam Sa-At.

^3^ NPJ = Nong Pla Jat.

^4^ ThP = Tham Pla (Pla cave).

^5^ ThNL = Tham Nam Lot (Nam Lot cave).

The degree of relatedness between individuals was determined using a pairwise test. Mean *r* values were calculated for all 93 blue mahseers from the five populations (S13 Table in S1 File). The distribution of *r* values exhibited a left skew, indicating lower pairwise *r* values than expected by chance under the null hypothesis of unrelated individuals. The distributions of pairwise *r* values differed (*p* < 0.05) for most population pairs, except for MNS–MNSA, MNS–NPJ, MNSA–NPJ, and MNSA–ThNL (S11A Fig); (https://datadryad.org/stash/share/h8uyRkLq7XVtcnFjKuUSQv8FyyfPH7sgZBBcrGG4HBA). The pairwise *F*_IS_ distributions exhibited a left skew and was comparable (*p* > 0.05, 0.057–0.873) for all population pairs (S11B Fig; S11–15 Tables in S1 File, https://datadryad.org/stash/share/h8uyRkLq7XVtcnFjKuUSQv8FyyfPH7sgZBBcrGG4HBA). The *N*_e_ for individual blue mahseer was highest in ThP, whereas the lowest was observed in ThNL (S10 Table in S1 File). Low Nei’s genetic distance (0.108–0.348) and *R*_ST_ (0.117–0.556) were observed among the four subpopulations (S14 Table in S1 File). After 1000 permutations, *F*_ST_ differed (*p* < 0.05, 0.024–0.262) between all populations, indicating that the populations were genetically differentiated. However, *F*_ST_^ENA^ estimates between the two populations were comparable (*F*_ST_^ENA^ = 0.142, *p* = 0.077). An analysis of molecular variance (AMOVA) showed that genetic variation was distributed in 14.94% of the population, with 7.690% attributable to within-population differences (S16 Table in S1 File).

**Fig 2:**
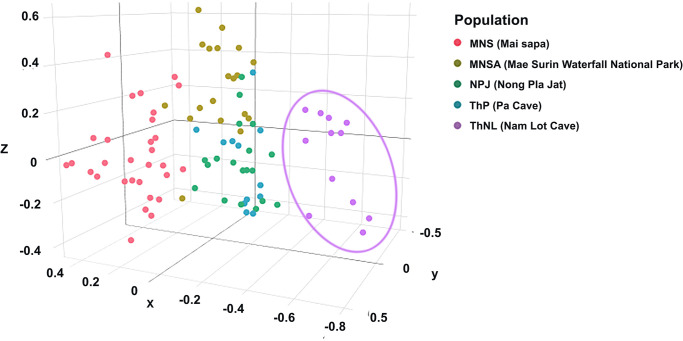
Principal coordinate analysis (PCoA) was calculated by GenAlEx version 6.5 of five blue mahseer (*Neolissochilus stracheyi*) populations based on 13 microsatellite loci. MNS (Mai sapa): Soi River, Subwatershed: Lower Mae Pai (Part 1); MNSA (Mae Surin Waterfall National Park): Sa-at River, Subwatershed: Lower Mae Pai (Part 1); NPJ (Nong Pla Jat): Sanghi River, Subwatershed: Lower Mae Pai (Part 1); ThNL (Nam Lot Cave): Tham Nam Lot, Subwatershed: Lower Mae Pai (Part 2).

**Fig 3:**
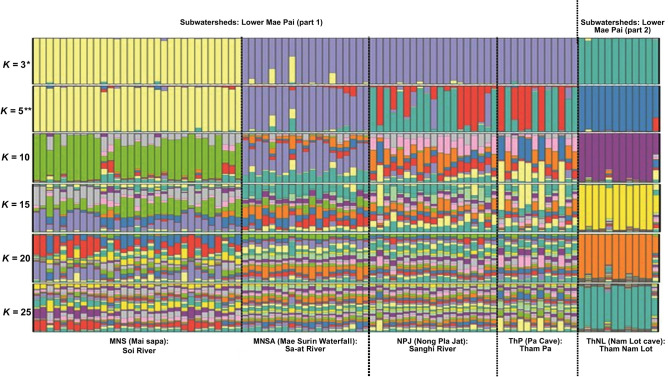
Population structure of five blue mahseer (*Neolissochilus stracheyi*) populations was analyzed using STRUCTURE version 2.3.4. Each vertical bar on the *x*-axis represents an individual, while the *y*-axis represents the proportion of membership (posterior probability) in each genetic cluster. The most probable number of *K*-values is represented by an asterisk (*) and star (✶), according to Evanno’s Δ*K* and ln Pr (X|*K*), respectively.

The PCoA results revealed three distinct clusters within blue mahseer population. Individuals from the ThNL and MNS populations clustered independently, whereas those from the MNSA, NPJ, and ThP populations showed similar clustering (Fig 2). This was corroborated by the DAPC results, which indicated three distinct separations (S12 Fig). The results of the model-based Bayesian clustering algorithms using STRUCTURE showed that blue mahseer populations exhibited different structural patterns for various *K*-values (2–25, Fig 3). The optimized population structure patterns were assigned to three clusters (*K* = 3) on the basis of Evanno’s Δ*K*; based on the mean ln P(*K*), the STRUCTURE analysis identified a single peak at *K* = 5 (S13 Fig). Under *K* = 3, the MNS and ThNL populations showed independent gene pools, whereas MNSA, NPJ, and ThP tended to share similar gene pools. Under *K* = 5, the MNS and ThNL populations showed independent gene pools. The MNSA population showed divergent gene pools from other populations, whereas NPJ and ThP shared a similar pattern. Under *K* = 25, the ThNL population exhibited unique gene pool patterns that were not shared with the other populations. Selective sweep analysis showed a tendency toward balanced selection, which was reflected by high *H*_e_ and low *F*_IS_ values, except for loci NY07, NY11, and NY14 (S14 Fig).

### Migration between populations

**Fig 4:**
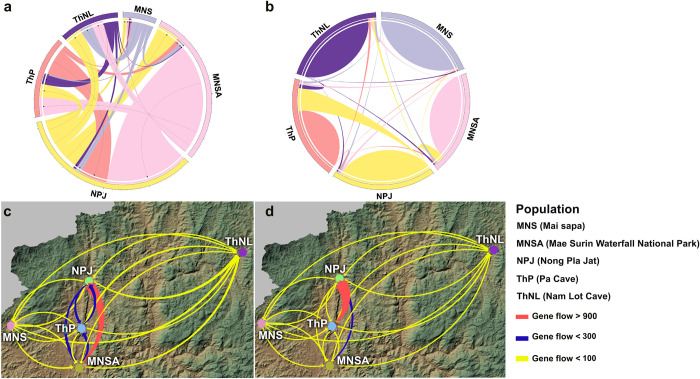
Source–sink migration revealed by Circos version 0.69–8 and gene flow dynamics visualized by QGIS 2.18. (**A**) Historical source–sink migration estimated using MIGRATE-N version 4.4.3.5. (**B**) Current source–sink migration estimated using BayesAss version 3.0. (**C**) Historical gene flow dynamics based on MIGRATE-N between blue mahseer populations across space. (**D**) Recent gene flow dynamics based on BayesAss between blue mahseer population over space. The width of the curves indicates the relative magnitude of migration. MNS (Mai sapa): Soi River, Subwatershed: Lower Mae Pai (Part 1); MNSA (Mae Surin Waterfall National Park): Sa-at River, Subwatershed: Lower Mae Pai (Part 1); NPJ (Nong Pla Jat): Sanghi River, Subwatershed: Lower Mae Pai (Part 1); ThNL (Nam Lot Cave): Tham Nam Lot, Subwatershed: Lower Mae Pai (Part 2).

Wilcoxon signed-rank tests for recent population bottlenecks generated a stepwise mutation model (SMM) and two-phase mutation model (TPM) of a normal L-shaped distribution for all populations (S17 Table in S1 File). The average *M* ratio for all populations was 0.200 ± 0.032. An *M* ratio of <0.68 indicated a historical reduction in the population [68] (Table 1). Recent gene flow estimates using BayesAss ranged from 0.686 ± 0.019 to 0.962 ± 0.017 within populations and from 0.01 ± 0.01 to 0.234 ± 0.036 between populations, with the highest gene flow observed from ThP to NPJ (Fig 4, S18 Table in S1 File). Microsatellite genotyping datasets were subsequently used in independent runs for the MIGRATE-N analysis to estimate historic gene flow (Fig 4, S19 Table in S1 File). A broad range of mutation-scaled immigration rates (*M*, 1.667–983.667) were observed in the MIGRATE-N analysis. The highest *M* value was observed from MNSA to NPJ, indicating that the migration rate relative to the mutation rate was highest from population MNSA to NPJ than among the other population pairs. Mutation-scaled population size (Θ) values (0.003–0.09863) were highest in the MNSA population, indicating that the effective MNSA population size was relatively large compared to the mutation rate. The ThNL population exhibited the lowest Θ values. In most populations, deficient gene flow is generally indicated by the calculated effective number of migrants per generation or the gene flow rate (*N*_m_). A broad range of *N*_m_ values was observed for gene flow among the five populations (7.667–983.667). The highest *N*_m_ value was observed from MNSA to NPJ, indicating relatively high gene flow between these populations.

**Fig 5:**
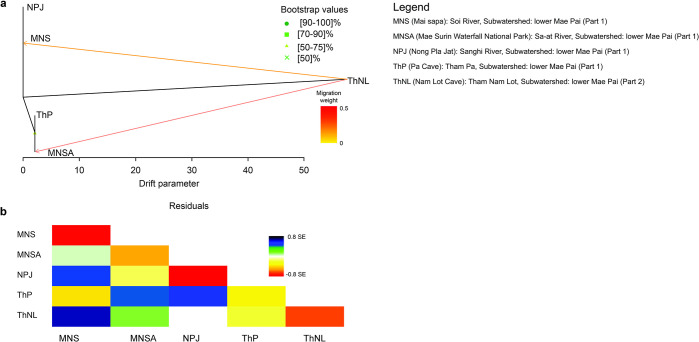
TreeMix-based maximum likelihood trees for blue mahseer (*Neolissochilus stracheyi*). (**A**) Maximum likelihood tree and (**B**) Residual fit for one migration event.

Based on the OptM function in TreeMix, the optimum number of major gene flow events was 2: (1) ThNL and inter-MNS–NPJ and (2) ThNL and MNSA (Fig 5A). After accounting for the two major introgression events, the residuals indicated the remaining potential admixtures within the five populations (Fig 5B). A significant correlation between population IBDs was observed (*r* = 0.927, *p* < 0.05), indicating a strong correlation between population differentiation based on *F*_ST_ and geographical distance (S15 Fig). Moreover, LSI analyses indicated the presence of genetically divergent areas in the five populations. Relatively high genetic divergence was detected in the NPJ and ThP populations, and low genetic divergence was detected in the MNS, MNSA, and ThNL populations (S16 and S17 Figs).

### Genetic variability of blue mahseer population based on mitochondrial DNA D-loop sequences

The amplicon and alignment lengths of the mtDNA D-loop sequences were 520–630 bp and 580 bp, respectively, with 11 haplotypes. Mean haplotype and nucleotide diversities were 0.828 ± 0.022 and 0.020 ± 0.001 (Table 2). A complex haplotype network was constructed using several polymorphic sites and haplotypes (S18 Fig). The overall theta (per site) from the *S* value and average number of nucleotide differences (*k*) were 0.013 and 11.416, respectively (Table 2). The *G*_ST_ value ranged from –0.023 to 0.405, while *Ф*_ST_ was 0.007–0.554. The *F*_ST_ value ranged from –0.056 to 0.701 and was significant for all population pairs, except for NPJ–ThP. The average number of nucleotide substitutions per site between populations (*D*_xy_) was 0.017–0.033, whereas the net number of nucleotide substitutions per site between populations (*D*_a_) ranged from –0.001 to 0.025 (S21 Table in S1 File). There was no statistical significance in Tajima’s *D* values (ranging from –1.747 to 1.554), and only one showed a significant difference (3.235). The Fu and Li’s F* and D* values ranging from 1.643 to 2.461 and 1.403 to 1.618, respectively, were significant. The Fu and Li’s F* and D* values ranging from –2.285 to 1.460 and 1.403 to 1.566, respectively, were nonsignificant. The Ramos–Onsins and Rozas’s R_2_ values were 0.187–0.276 (S22 Table in S1 File). The EBSPs based on mtDNA D-loop sequences showed a decreasing population size from 2012 to 2022 (S19 Fig). Mismatch distribution analysis of each population dataset exhibited a unimodal distribution for the MNS, MNSA, and ThP populations, whereas the NPJ and ThNL populations showed a bimodal distribution (S20 Fig). The raggedness index values were 0.091–0.750, with no significant difference (S22 Table in S1 File). *M* based on the mtDNA D-loop sequence varied from 4.300 to 236.300, with the highest value observed between ThP and NPJ. Mutation-scaled population size (Θ) values were 0.098–0.099, with the highest value observed in MNS and ThP (S23 Table in S1 File). A broad range of *N*_m_ values was observed (0.106–5.795), with the highest observed from ThP to NPJ (S24 Table in S1 File).

**Table 2:** Mitochondrial DNA D-loop sequence diversity for blue mahseer (*Neolissochilus stracheyi*).

Population	N	Number of haplotypes (H)	Theta (per site) from *S*	Average number of nucleotide differences (*k*)	Haplotype diversity	Nucleotide diversities (*π*)
MNS ^1^	31	2	0.007	7.845	0.490± 0.045	0.013±0.001
MNSA ^2^	19	3	0.008	6.246	0.433±0.117	0.011±0.002
NPJ ^3^	19	5	0.013	9.357	0.778± 0.055	0.016±0.002
ThP ^4^	12	5	0.015	11.167	0.803±0.090	0.019±0.003
ThNL ^5^	12	2	0.002	0.667	0.167±0.134	0.001±0.001
All populations	93	11	0.013	11.416	0.828±0.022	0.020±0.001

^1^MNS = Mae Nam Soi.

^2^ MNSA = Mae Nam Sa-At.

^3^ NPJ = Nong Pla Jat.

^4^ ThP = Tham Pla (Pla Cave).

^5^ ThNL = Tham Nam Lot (Nam Lot cave)

### Species distribution and landscape

Potential niches and probability distribution maps were predicted using MaxEnt models based on occurrence records. The area under the curve (AUC) estimates are presented in S21 Fig. The models for this species exhibited mean AUCs near 1, indicating their effectiveness as classifiers. The permutation importance (%) of the major predictors in the distribution of this species is shown in S22 Fig "Distance to river" (proximity to main river) was the most significant predictor of blue mahseer distribution, followed by "annual mean total precipitation" (1280–1300 mm), "slope" (0–5°, flat), "NDWI" (0.15–0.30, indicating water surface), and "NDVI" (0.35–0.55, representing shrubs and dense grassland vegetation or tropical rainforest). The jackknife test revealed that "distance to river" and "annual mean total precipitation" were the most important predictors and presented higher gains compared to the other variables. The likely duration of blue mahseer species presence in different watershed rivers was estimated by calculating their extent of occurrence range for each class, using defined probability classes at a threshold of ≥0.5, at which point the species is considered to be present. The suitability of land areas was classified as highly suitable (*p* > 0.75; comprising 99 km^2^, 0.77% of the total area), moderately suitable (0.75 ≤ *p* ≤ 0.50; 194 km^2^ or 1.52%), and least suitable (*p* < 0.5; 12 487 km^2^ or 97.71%; S23 Fig).

## Discussion

The distribution of blue mahseers has been documented in Thailand beyond Mae Hong Son. However, most observations occur within protected areas (PAs), such as national parks, particularly in waterfalls and stream habitats [69]. In Mae Hong Son, blue mahseers were observed in three PAs (MNSA: Namtok Mae Surin National Park, ThP: Tham Pla-Pha Suea National Park, and ThNL: Tham Lot Nature and Wildlife Education Centre) and two protected public waterways (Mai Sapa and NPJ: Nong Pla Jat in Lower Mae Pai Part 2). Using *COI* and *Cytb* DNA barcoding for species identification, all blue mahseer specimens were identified as *N*. *stracheyi* without any sequence divergence. This indicates that all populations had the same CU. Allocating resources to distinct ecological, genetic, and phylogenetic intraspecific-level groups is optimal for species protection, but is complicated by habitat fragmentation from natural and human activities.

### Low genetic diversity without signs of inbreeding or outbreeding

From the genetic diversity of the species, insights into their evolutionary history and adaptation were derived, with a potential limitation on establishment posed by dispersal patterns and decreases in diversity. Low heterozygosity (<0.5) was consistent across all populations. Similarly, most studies have emphasized a decline in genetic diversity across mahseer populations [70–72]. No differences between *H*_o_ and *H*_e_ were observed, indicating no signs of inbreeding or outbreeding effects. Interestingly, the consistency between low *F*_IS_ and *r* values suggested nearly random mating within geographic populations, despite the continuous observation of low *N*_e_ values. This suggests that only a limited number of allelic resources are available. The high *AR* values reported in rare minnow (*Gobiocypris rarus*, 4–7) and Atlantic salmon (*Salmo salar*, 6–13) [73, 74] contrasted with the low *AR* (*AR* = 2–3) found in this study. *AR* is crucial for conservation and indicates the adaptability, persistence potential, and long-term survival of a population [75]. A forecasted drop in *H*_e_ and *AR* to below 0.1 and 2, respectively, within 50 years was shown by forward simulation, suggesting a crisis in the genetic resources of blue mahseer in Mae Hong Son. Low positive *F*-values were observed in the MNSA, NPJ, and ThNL populations, indicating that habitat adaptation and contemporary environmental conditions probably influenced the population structure, leading to diversity structuring within populations (S24 Fig) [76]. From an alternative perspective on maternal DNA inheritance via the mtDNA D-loop, blue mahseer showed a broader range of genetic diversity (*h* = 0.167–0.803, π = 0.001–0.019) compared to, for example, chocolate and golden mahseer (*h* = 0.000–0.977, π = 0.000–0.016; *h =* 0.629–0.889, π = 0.000–0.001, respectively) [77, 78]. Assuming independence from sample size among the populations, high *h* values were observed in the NPJ and ThP populations, both of which are located in the Sanghi River. In contrast, a low *h* value was found in the MNSA population located in the Sa-at River (the same river system as the Sanghi River) and connected downstream in the Lower Mae Pai (Part 1) sub-watershed. This was consistent with the results obtained from the LSI analysis, which was calculated using microsatellite genotyping. This suggests larger genetic diversity upstream, correlating with habitat suitability, "distance to river," and "annual mean total precipitation," being the most important predictors for the probable distribution of blue mahseer. No samples were analyzed from sites upstream of the Soi River before the MNS population and the Mae Lang River in the Lower Mae Pai (Part 2) sub-watershed before the ThNL population, but similar cases have been discussed [79]. Low genetic diversity may be influenced by the occurrence of weirs, reservoirs, or small dams with small in-stream infrastructure or natural damming, such as that formed by landslides in some study sites [52]. This is supported by studies on other taxa, in which small in-stream infrastructure and upstream ranges may contribute to the maintenance of ancient lineages and species diversity [80, 81]. However, the results are probably limited by small sample size, with factors such as genetic patchiness (Wahlund effect), reduced *N*_e_, and null alleles contributing to heterozygote deficiency. The high polymorphism of most loci suggests that their value in estimating genetic diversity necessitates further research in Mae Hong Son.

### Understanding blue mahseer clusters in Mae Hong Son and their genetic diversity maintenance

This investigation focused on genetic variation in Mae Hong Son’s blue mahseer to identify long-term evolutionary and ecological influences. Three genetic clusters were identified using STRUCTURE analysis, which revealed clear spatial patterns. A relatively low genetic admixture (individual q value > 0.8) was observed in all blue mahseer at *K* = 3, suggesting weak differentiation among individuals in each population. This along with the AMOVA results indicated higher genetic variation among than within populations. These genetically uncontaminated populations are thus ideal sources of fresh alleles for future conservation efforts. Three spatially distinct clusters were observed in the Soi (MNS), Sanghi-Sa-at (NPJ, ThP, and MNSA), and Mae Lang (ThNL) rivers. Clustering patterns identified through Bayesian STRUCTURE analysis were similar to those in the PCoA and DAPC, aligning with the major river systems and sub-watersheds from which the blue mahseer samples were obtained. Higher possible inter-population gene flow was observed within the Sanghi-Sa-at River system. At higher *K*-levels, the gene pool of the NPJ and ThP populations diverged from that of the MNSA population, indicating that the Sanghi-Sa-at River system encompasses two subclusters—subsets of a cluster with minor yet significantly distinct alleles and/or haplotypes. Significant *F*_ST_ values derived from both microsatellite genotyping and mtDNA D-loop sequencing indicated differentiation among the five populations, except between the NPJ and ThP populations, which belonged to the same cluster according to the mtDNA D-loop analysis. Notably, low migration rates may contribute to high *F*_ST_ values. To estimate population differentiation, *F*_ST_ and *R*_ST_ statistics, which measure connectivity and gene flow patterns among populations, were used. Among most populations, higher *R*_ST_ values than *F*_ST_ were observed. *F*_ST_, assuming the infinite allele model, considers mutation and migration, whereas the independence of *R*_*ST*_ from mutation rate is based on the stepwise mutation model (SMM) [82]. This suggests that mutations, rather than migration, were implicated in the high genetic differentiation observed among blue mahseer populations until recently [83]. A strong correlation was observed between the genetic (*F*_ST_) and geographical distances of populations, with the differentiation of population structure following linear trends in IBD. This suggests that the genetic structure of blue mahseer populations in Mae Hong Son may be affected by geographical distance. Demographic analysis indicated that no recent bottleneck events affected the genetic diversity and structure of Mae Hong Son’s blue mahseer. GIS mapping indicated a clear separation of sampling locations, which could obstruct any active migration or intermixing of individuals, further substantiating the findings of the migration analysis. For instance, the ThP population, located in a nearly closed system within a cave and several weirs in the PA, is challenged by the limited inflow or outflow of individuals, except during flooding events common in Mae Hong Son [52]. A scenario of population expansion was identified based on the unimodal and bimodal distributions revealed by mismatch analysis. Neutrality statistics, a significant raggedness index, and EBSPs indicated a nearly constant size over time for all populations. These findings support the resilience of blue mahseer to recent natural and anthropogenic threats.

Limited historical gene flow was observed between the clusters, potentially influencing the occurrence of the clusters. A long historical bottleneck in all populations was revealed, possibly relating to ≥50 generations with a relatively low *M* ratio. A relatively low nucleotide diversity, which might be attributed to a possible bottleneck during earlier colonization, supported the low level of haplotype diversity, consistent with previous findings [72]. This suggests that the occurrence of these clusters may have resulted from historically limited gene flow and genetic drift. This agreed with the *N*_m_ values of >1 across the overall population, indicating that genetic drift was likely not the primary factor responsible for genetic differentiation. However, gene flow between subclusters within the Sanghi-Sa-at River may be facilitated by flooding during the rainy season or potamodromous homing behavior in strictly clear headstream waters where long-distance migrations (over 120 km) may occur [18]. Therefore, mating and spawning may have occurred in the same stream. Human-mediated translocation may be possible; however, no evidence has been reported, and only precautions regarding such activity can be taken. Further testing of this hypothesis would require a larger sample size and additional molecular markers.

### Ancestral genetic admixture and introgression

**Fig 6:**
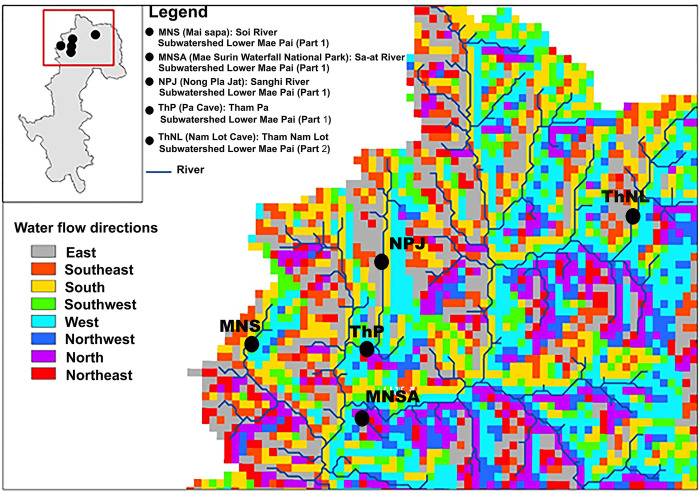
Water flow patterns, analyzed using QGIS version 3.34.8 and data from http://sql.ldd.go.th/ldddata/mapsoilB1.html, potentially facilitated gene flow among blue mahseer populations.

Interpretation of the observed genetic relationships among freshwater fish populations is based on the phylogeographical species distribution, which is strongly influenced by present and historical river connections [84]. Mae Hong Son’s freshwater habitats are separated by the Daen Lao Range Mountain, which acts as a geological barrier, and sourced from the headwaters of the mountain, with rivers extending into the Salween River watershed from the northeast to the south and west [52]. Remarkably, the Soi, Sanghi-Sa-at, and Mae Lang rivers, which drain into the Salween River, are isolated by lower ranges and hills due to separation by mountain ridges, leading to disconnections or connections only downstream among neighboring rivers. Blue mahseer movement is restricted by this isolation, resulting in genetic differentiation and distinct lineages within the unconnected river systems. However, the TreeMix analysis revealed ancestral gene flow from the ThNL population to the west and south, likely before blue mahseer colonization during low historical genetic differentiation. A similar result was predicted from the haplotype network, where no sharing of haplotypes was observed between the ThNL and other populations. This suggests that large proportions of ancestral genotypes are retained by the Mae Lang River in the Lower Mae Pai (part 2), marking it as the origin of colonization before expansion to the western and southern clades. Geomorphological phenomena, such as floods depicted in the waterflow landscape (Fig 6) or historical connections between river systems, may facilitate opportunities for blue mahseer migration across rivers and drainage systems, leading to historical introgression. This allowed for a potential admixture or shared gene pool among the MSN, MNSA, ThP, and NPJ populations. The low introgression observed from ThNL to ThP compared with other populations could be attributed to the nearly closed system (cave) and small weirs in the PAs.

### Impacts on conservation efforts and potential solutions

Effective conservation management requires the estimation of genetic diversity and population structure. For blue mahseer, conservation hinges on genetic insights, especially regarding genetic diversity and spatial patterns, to define MUs and craft propagation plans. In short-term conservation management, genetic stocks, which are comparable to MUs, are the major unit for maintaining genetic resources and local abundance by avoiding overexploitation. By contrast, in long-term conservation, genetic stocks are equated to ESUs, representing key facets of adaptive diversity and evolutionary potential, with a focus on sustaining viable local populations. The current study showed that blue mahseer populations appear to be significantly structured into geographically distinct clusters. Intense deforestation and exploitation in the recent history of Mae Hong Son limited blue mahseer movement, leading to the lower expected heterozygosity. Habitat modification due to logging, agricultural activities, and illegal fishing are the primary local threats to aquatic biodiversity in Mae Hong Son, and these impacts are likely exacerbated by extreme global climate change [85]. Climate change is recognized as a significant threat to global biodiversity; however, freshwater fishes have often been overlooked in climate change assessments [86]. In this study, it is demonstrated that high-quality distributional data, which improves ecological understanding of the species, can also be used to predict the future effects of climate change. All clades were historically isolated, phylogenetically distinct, and had a high level of genetic differentiation, fulfilling the ESU criteria. This collectively suggests the existence of three ESUs in within Mae Hong Son’s blue mahseer: (1) MSN, (2) MNSA–ThP–NPJ, and (3) ThNL, broadly defined as phylogenetically distinct intraspecific groups, were identified across the natural geographical range, despite significant genetic differentiation. According to the stream hierarchy model, genetic structuring and differentiation in obligate freshwater fish populations, are often driven by gene flow restrictions and dispersal limitations within watersheds [85]. Influences on genetic variation in riverine freshwater fish include IBD; barriers such as weirs, reservoirs, and dams; and resistance factors such as temperature and stream gradient. This is supported by the finding that contemporary species distribution patterns may have been shaped by climate change and geomorphological phenomena through historical dispersal, leading to population differentiation owing to geographical isolation. Maintenance of the three ESUs in Mae Hong Son is important for the long-term viability of the population.

Two MUs (MNSA and ThP–NPJ), defined as ESU subsets with minor yet significantly distinct alleles and/or haplotypes, encompassed the Sanghi-Sa-at River system. The ThP and NPJ populations were designated as the same MU and characterized by a shared gene pool and the highest rate of gene flow. The delineation of each ESU/MU in this study can be used to guide existing management measures, such as stocking and monitoring programs. Each ESU/MU consists of members from a distinct stock with limited dispersal ability, suggesting limited potential for local recruitment from other sub-watersheds or different ESUs/MUs. Consequently, local population decline due to illegal fishing is unlikely to be compensated for, at least in the short term, by recolonization from other ESUs/MUs. To minimize the negative genetic impacts of stocking programs/translocations, such as outbreeding depression, loss of genetic diversity, and loss of inter-population variation, it is recommended that stocking be avoided between ESUs/MUs, that is, separate management of each ESU/MU is advised, with no interclade stocking. Blue mahseer should only be released into the same ESU/MU from which they were collected to prevent the introduction of different genetic stocks that may hybridize with the local population. Further implementation of monitoring programs to prevent illegal fishing based on the delineation of ESUs/MUs is highly recommended.

## Conclusions

This study classified blue mahseer populations in Mae Hog Son into three ESUs, with one further divided into two MUs. Significant genetic differentiation was observed between groups. When implementing conservation measures, it is recommended that the ESUs/MUs enjoy specific management programs. The correlation between genetic differentiation and geographical distance among populations suggested that habitat conditions are the primary factors influencing blue mahseer genetic structure. The low genetic diversity observed across all populations highlights the urgent need for conservation attention, with a forecasted drop in heterozygosity below 0.1 within 50 years. Stocking between ESUs or MUs is discouraged to avoid negative genetic impacts. The insights gained from this study on population structure can inform the development of stock-specific conservation and management strategies for the long-term maintenance of free-living blue mahseer populations and the associated tourism economy.

## Supporting information

S1 Fig Distance-based comparison of efficiency among barcoding markers for mahseer species in the database. (**A**) Distribution of barcoding gaps, defined by the difference between minimum and maximum intraspecific distance. (**B**) Percentage of correct identifications from the nearest neighbor test. A tree-based comparison of efficiency among the barcoding markers for mahseer species from the database using the percentage of monophyletic groups recovered between cytochrome c oxidase I (*COI*) and cytochrome b (*Cytb*).
(DOCX)

S2 Fig DAMBE substitution saturation plots for public repository-based whole sequences of *COI* (**A**), *COI* codon positions 1 and 2 (**B**), *COI* codon position 3 (**C**), whole sequences of *Cytb* (**D**), *Cytb* codon positions 1 and 2 (**E**), and *Cytb* codon position 3 (**F**).
(DOCX)

S3 Fig Distribution of maximum intraspecific (orange line) and minimum interspecific (green line) Kimura’s-two-parameter (K2P) divergence of 14 species based on cytochrome c oxidase I (*COI*) and 12 species based on cytochrome b (*Cytb*). (DOCX)

S4 Fig Phylogram clarifying the phylogenetic relationships among the 659 GenBank accessions, constructed from a Bayesian inference analysis using mitochondrial cytochrome c oxidase I (*COI*) sequences. (DOCX)

S5 Fig Phylogram clarifying the phylogenetic relationships among the 509 GenBank accessions, constructed from a Bayesian inference analysis using mitochondrial cytochrome b (*Cytb*) sequences. (DOCX)

S6 Fig Phylogram clarifying the phylogenetic relationships among 659 GenBank accessions, constructed from Bayesian inference analysis using mitochondrial cytochrome c oxidase I (*COI*) sequences. Group 1: higher-level similarity with the same species. Group 2: higher-level similarity with multiple species. Group 3: unique sequences with no similarity within most sequences. Class 1: sequences with the same species name exhibiting intraspecific cohesive clustering and interspecific distinct clustering with high posterior probability (0.90–1.00). Class 2: sequences with the same species name that do not exhibit intraspecific cohesive clustering. Class 3: sequences with a different species name exhibiting cohesive clustering. There is only one accession number (*).
(DOCX)

S7 Fig Phylogram clarifying the phylogenetic relationships among 509 GenBank accessions, constructed from Bayesian inference analysis using mitochondrial cytochrome b (*Cytb*) sequences. Group 1: higher-level similarity with the same species. Group 2: higher-level similarity with multiple species. Group 3: unique sequences with no similarity within most sequences. Class 1: sequences with the same species name exhibiting intraspecific cohesive clustering and interspecific distinct clustering with high posterior probability (0.90–1.00). Class 2: sequences with the same species name that do not exhibit intraspecific cohesive clustering. Class 3: sequences with a different species name exhibiting cohesive clustering. There is only one accession number (*).
(DOCX)

S8 Fig Phylogram clarifying the phylogenetic relationships among the collected sample sequences from 15 blue mahseer, constructed from a Bayesian inference analysis using mitochondrial cytochrome b (*COI*) sequences. The common *Tor douronensis* was identified as an outgroup.
(DOCX)

S9 Fig Phylogram clarifying the phylogenetic relationships among the collected sample sequences from 15 blue mahseer, constructed from a Bayesian inference analysis using mitochondrial cytochrome b (*Cytb*) sequences. The common *Tor douronensis* was identified as an outgroup.
(DOCX)

S10 Fig Simulation results showing relationships between generations; (**A**) heterozygosity, and (**B**) allelic richness.
(DOCX)

S11 Fig (**A**) Observed distribution of inbreeding coefficients (*r*) in blue mahseer (*Neolissochilus stracheyi*), plotted against expected distributions. (**B**) Observed distribution of relatedness (*F*_IS_) in blue mahseer, plotted against expected distributions.
(DOCX)

S12 Fig Discriminant analysis of principal components (DAPC) for blue mahseer (*Neolissochilus stracheyi*) based on 13 microsatellite loci. (DOCX)

S13 Fig Different population structure patterns of 93 blue mahseer based on the genotyping of 13 microsatellite loci generated by the model-based Bayesian clustering algorithms in STRUCTURE. (**A**) Plot based on Evano’s Δ*K* and (**B**) ln P(*K*).
(DOCX)

S14 Fig Spatial distribution of genetic diversity in blue mahseer populations. (**A**) Expected heterozygosity (*H*_e_). (**B**) Inbreeding coefficients (*F*_IS_).
(DOCX)

S15 Fig IBD test (10,000 permutations) showing correlation among geographical and genetic distances (FST) between five different populations. (DOCX)

S16 Fig Results of genetic landscape shape interpolation analysis using an 80 × 80 grid. *x* and *y* axes correspond to geographic locations within the populations.
(DOCX)

S17 Fig Mapping of expected heterozygosity (*H*_e_) and inbreeding coefficients (*F*_IS_) using QGIS version 3.34.8 (**A**) *H*_e_ and *F*_IS_ values in blue mahseer from Soi River (Mai Sapa), Sa-at River (Mae Surin Waterfall National Park), Sanghi River (Nong Pla Jat), Tham Pa (Pa Cave), and Tham Nam Lot (Nam Lot Cave) populations. (**B**) *H*_e_ and *F*_IS_ values at 13 microsatellite loci.
(DOCX)

S18 Fig Haplotype network of three populations of blue mahseer (*Neolissochilus stracheyi*) based on mitochondrial DNA D-loop sequences. (DOCX)

S19 Fig Historical demographic fluctuations in the mtDNA D‐loop sequences of blue mahseer (*Neolissochilus stracheyi*) according to coalescent Bayesian skyline analysis. The median effective population size is delimited by the black lines. The blue shaded area delimits the upper and lower bounds of the 95% highest posterior density interval. The *x*-axis represents time in years and *y*-axis is displayed in logarithmic scale.
(DOCX)

S20 Fig Mismatch distribution of the mitochondrial DNA D-loop sequences in five populations of blue mahseer. (**A**) Soi River (Mai sapa), (**B**) Sa-at River (Mae Surin Waterfall National Park), (**C**) Sanghi River (Nong Pla Jat), (**D**) Tham Pa (Pa Cave), and (**E**) Tham Nam Lot (Nam Lot Cave) population. The *x*-axis represents the number of pairwise differences (mismatches) and the *y*-axis represents the frequency of these differences. The frequency distribution of the observed mismatches (red line) is compared to that of the expected mismatches (green line).
(DOCX)

S21 Fig Diagnostic performance of blue mahseer classification model. Area under curve (AUC) between average model sensitivity and specificity.
(DOCX)

S22 Fig Important predictor variables and their response curve for blue mahseer with the percent contribution (average of five replicate runs). The *x*‑axes represent the prediction probabilities between 0 (absent) and 1 (100% present). Distance to Rivers (51.7%), annual mean total precipitation (40.8%), slope (3.7%), normalized difference water index (NDWI, 1.1%), normalized difference vegetation index (NDVI, 0.9%), aspect (0.8%), elevation (0.4%), and flow directions (0.4%).
(DOCX)

S23 Fig Predicted habitat suitability for blue mahseer generated by MaxEnt version 3.4.4 in Mae Hong Son Province, Thailand. (DOCX)

S24 Fig Environmental variables were used to assess the species distribution model of the blue mahseer. These include: (**A**) annual mean temperature (°C), (**B**) annual total precipitation (mm), (**C**) elevation, (**D**) distance to rivers, (**E**) slope, (**F**) aspect, (**G**) flow direction, and (**H**) flow accumulation. The data for variables (A–H) were obtained from the Land Development Department (2021) and are free for use with no restrictions. Additionally, (I) NDWI and (J) NDVI were derived from Landsat 8 satellite images taken from January to April 2023. These images were accessed via the USGS Earth Explorer (2023), and Landsat data is freely available for use. More information can be found at: https://www.usgs.gov/landsat-missions/landsat-8.
(DOCX)

S1 File (DOCX)
