# Supplementary figures and images for: Genetic and geographical insights call for early conservation of Mae Hong Son’s blue mahseer to prevent population crisis
Source: PLoS One. 2025 Feb 12;20(2):e0313505. doi: 10.1371/journal.pone.0313505 (PMC12143899; doi:10.1371/journal.pone.0313505)

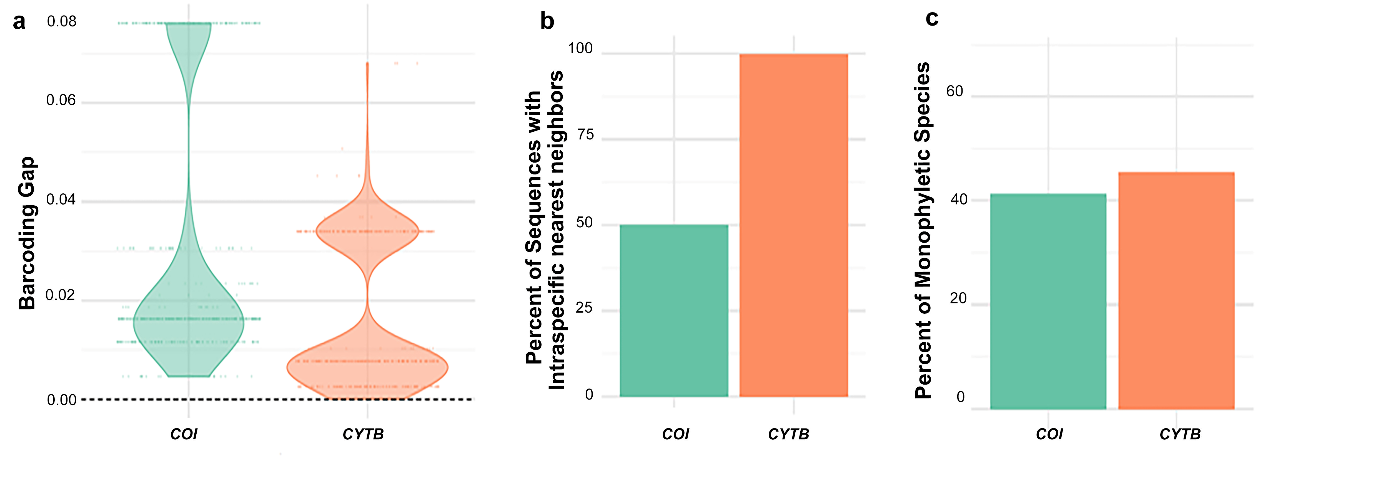


**Fig. S1.** (Pongsanarm et al.)

Supplement: S1 Fig — (A) Distribution of barcoding gaps, defined by the difference between minimum and maximum intraspecific distance. (B) Percentage of correct identifications from the nearest neighbor test. A tree-based comparison of efficiency among the barcoding markers for mahseer species from the database using the percentage of monophyletic groups recovered between cytochrome c oxidase I (COI) and cytochrome b (Cytb). (DOCX) [file pone.0313505.s001.docx]

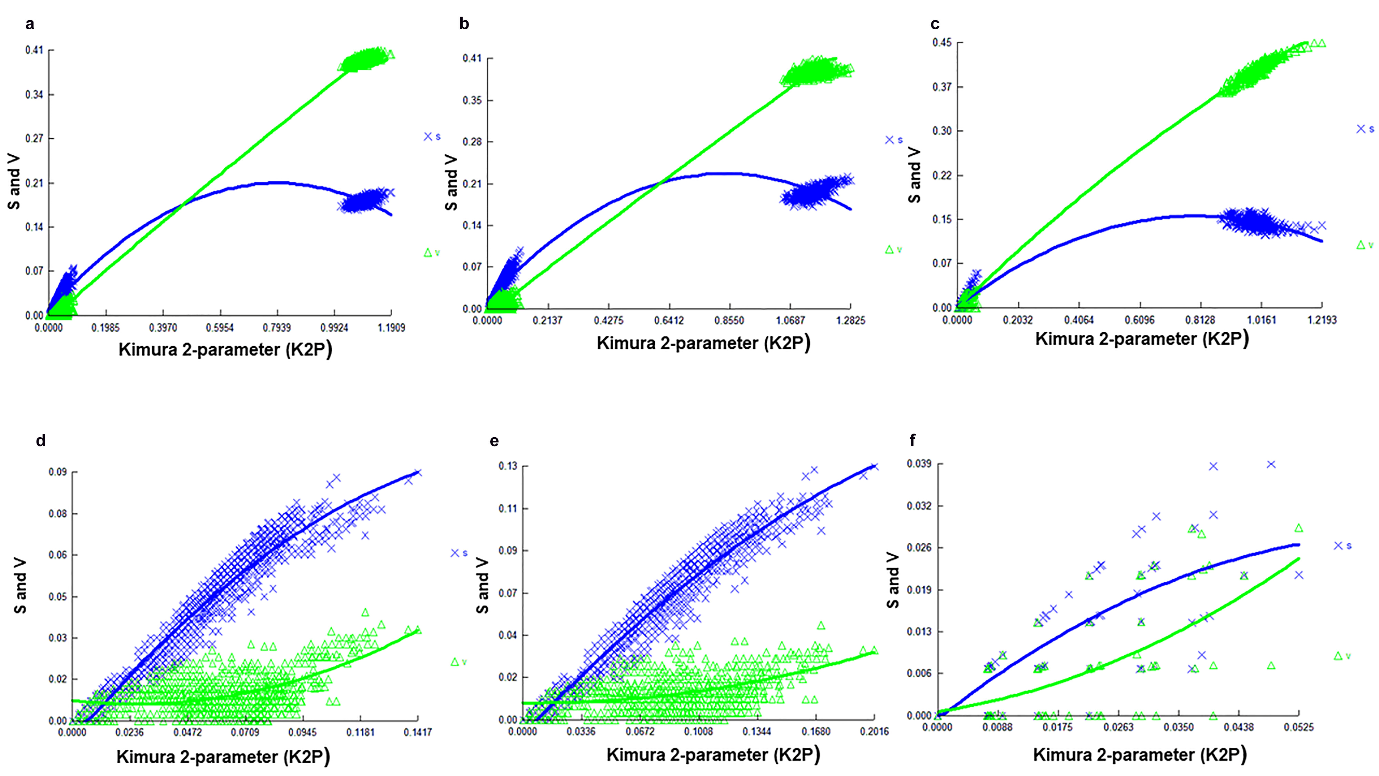


**Fig. S2.** (Pongsanarm et al.)

Supplement: S2 Fig — DAMBE substitution saturation plots for public repository-based whole sequences of COI (A), COI codon positions 1 and 2 (B), COI codon position 3 (C), whole sequences of Cytb (D), Cytb codon positions 1 and 2 (E), and Cytb codon position 3 (F). (DOCX) [file pone.0313505.s002.docx]

**
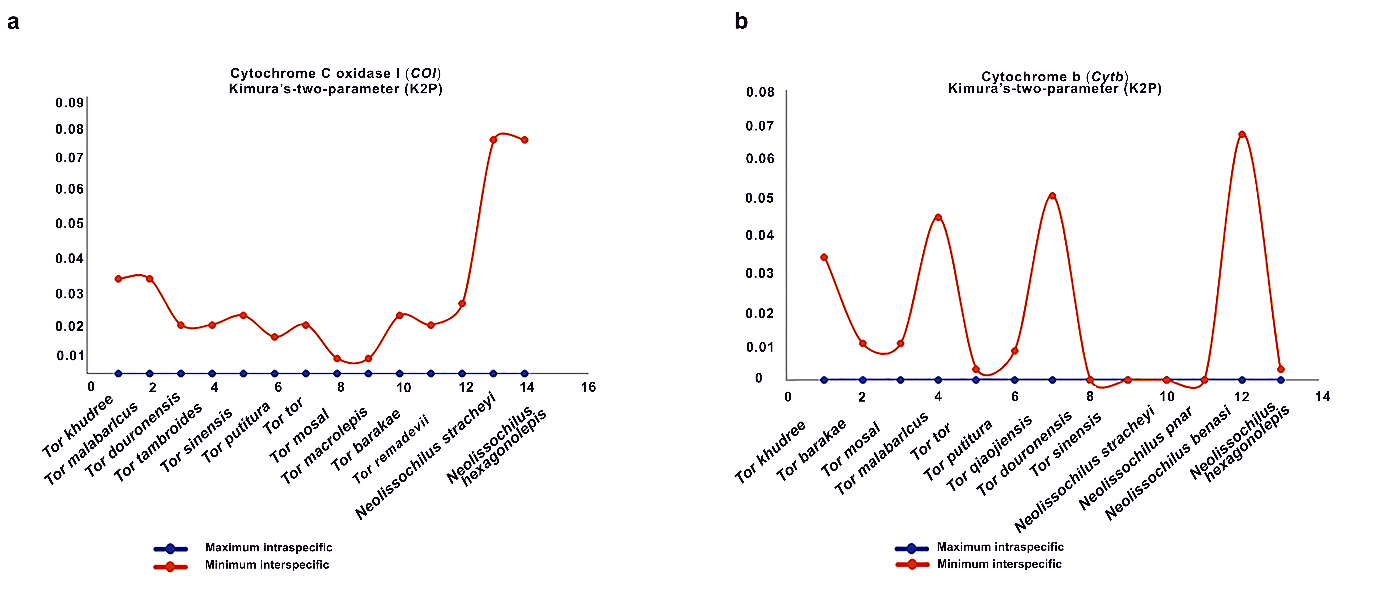
**

**Fig. S3.** (Pongsanarm et al.)

Supplement: S3 Fig — (DOCX) [file pone.0313505.s003.docx]

**
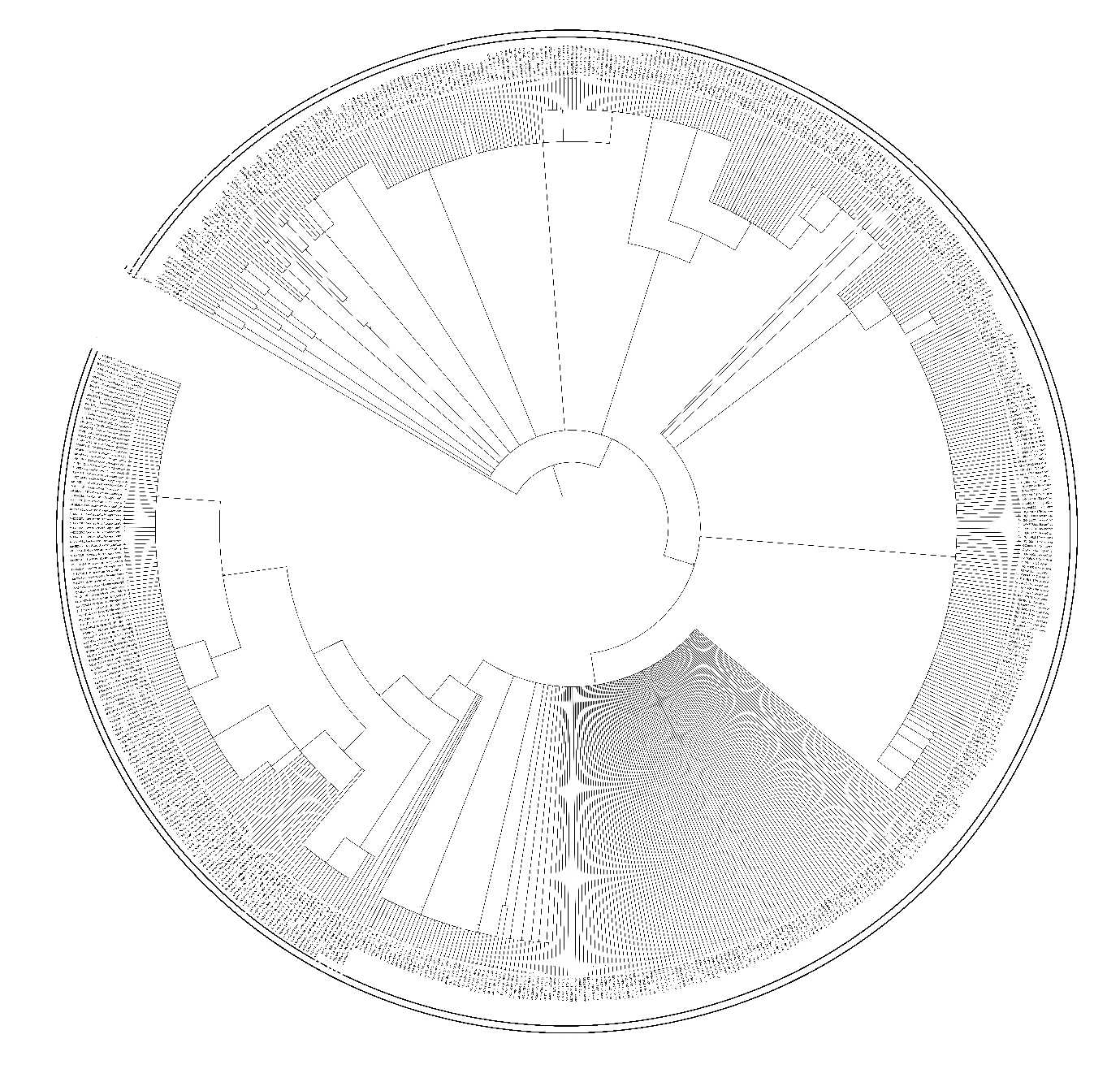
Fig. S4.** (Pongsanarm et al.)

Supplement: S4 Fig — (DOCX) [file pone.0313505.s004.docx]

**
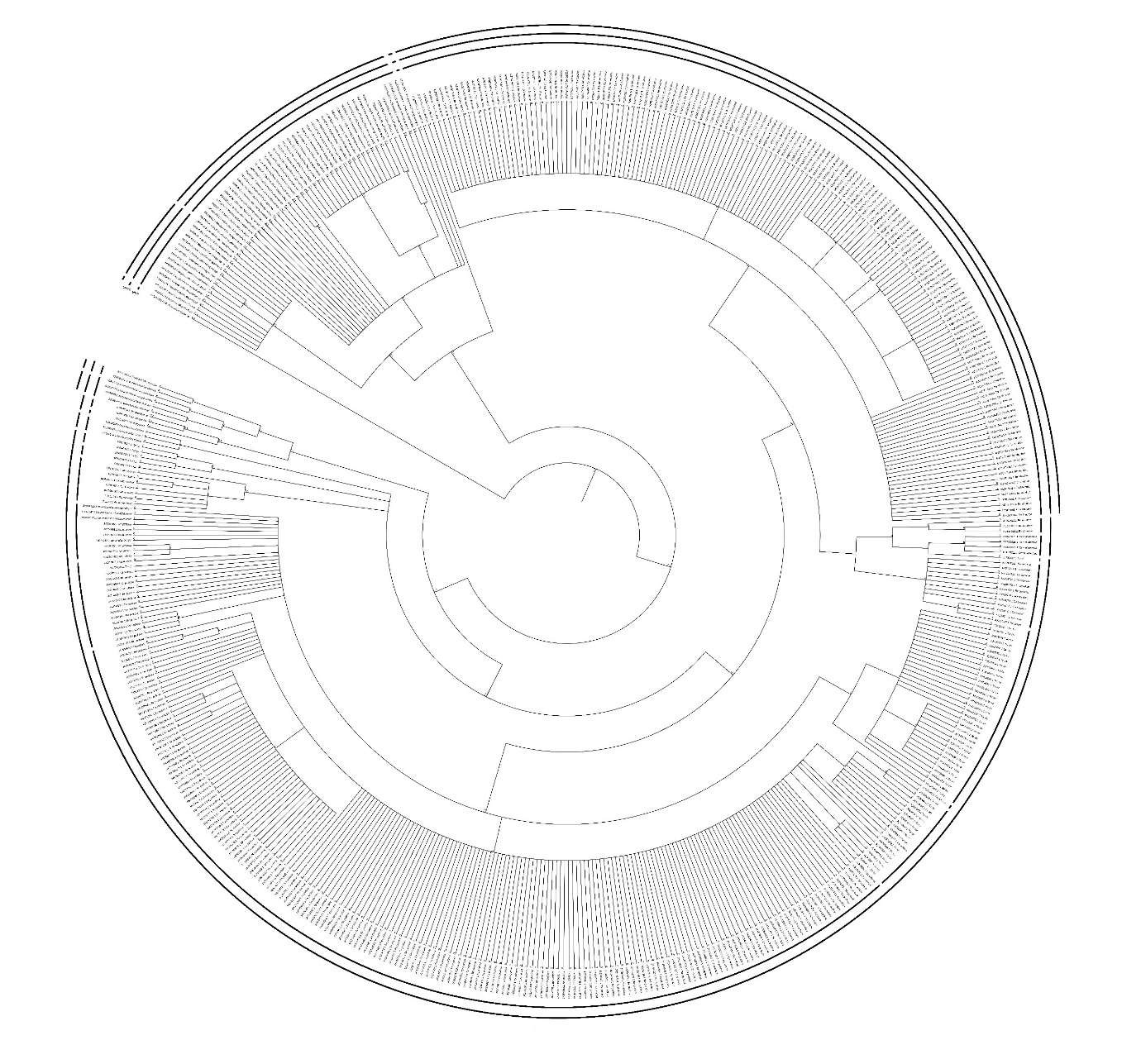
Fig. S5.** (Pongsanarm et al.)

Supplement: S5 Fig — (DOCX) [file pone.0313505.s005.docx]

**
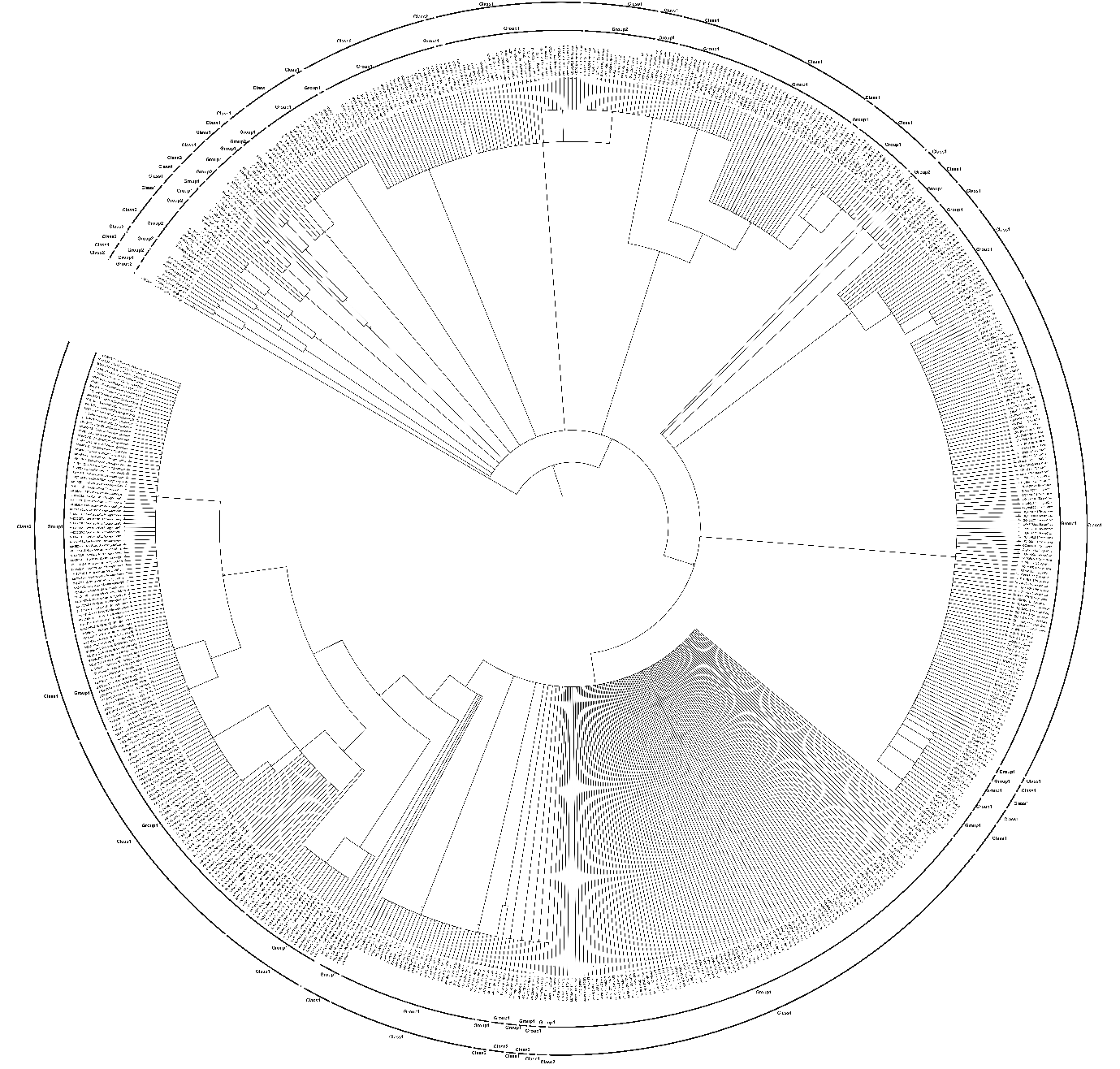
Fig. S6.** (Pongsanarm et al.)

Supplement: S6 Fig — Group 1: higher-level similarity with the same species. Group 2: higher-level similarity with multiple species. Group 3: unique sequences with no similarity within most sequences. Class 1: sequences with the same species name exhibiting intraspecific cohesive clustering and interspecific distinct clustering with high posterior probability (0.90–1.00). Class 2: sequences with the same species name that do not exhibit intraspecific cohesive clustering. Class 3: sequences with a different species name exhibiting cohesive clustering. There is only one accession number (*). (DOCX) [file pone.0313505.s006.docx]

**
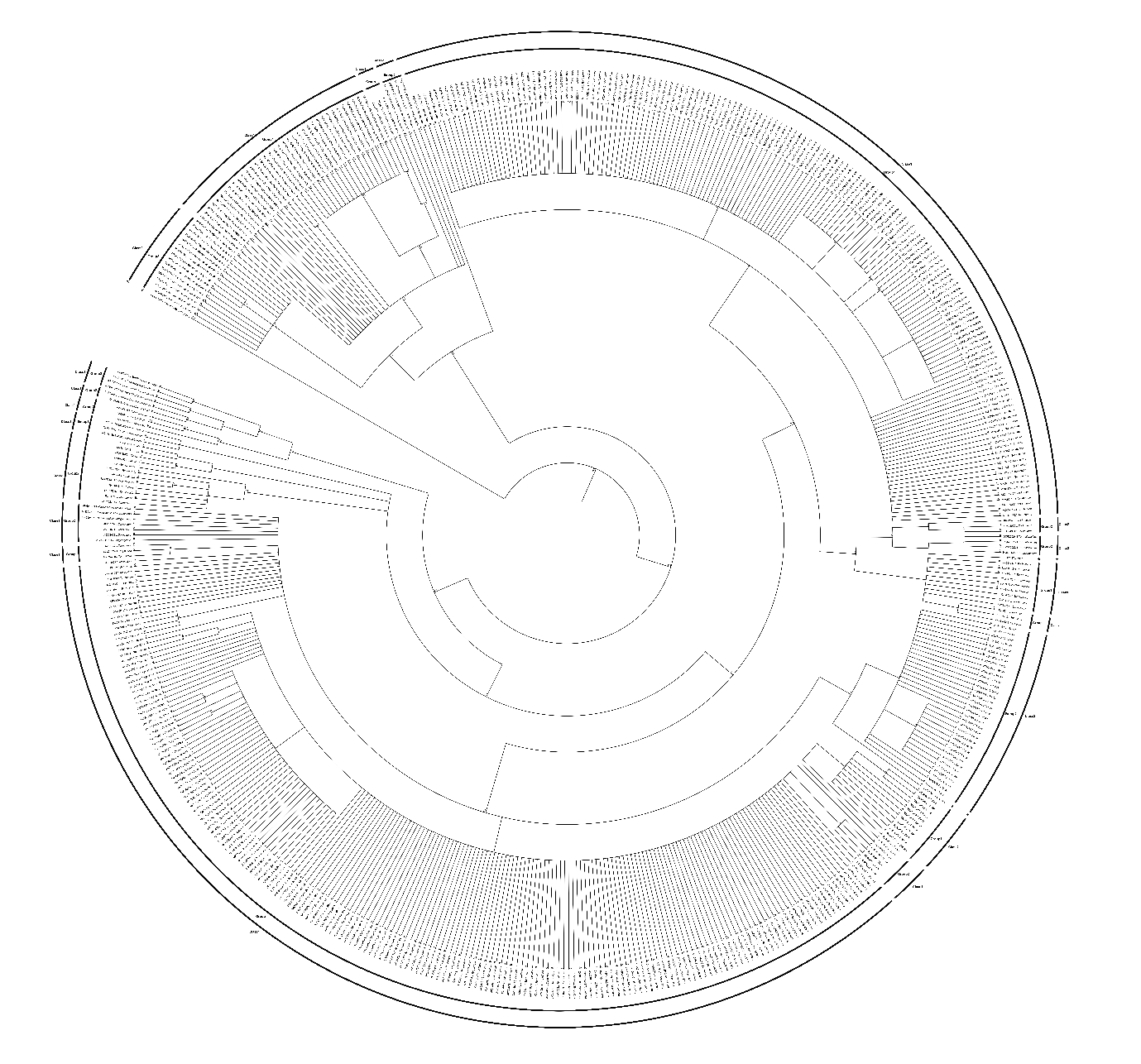
Fig. S7.** (Pongsanarm et al.)

Supplement: S7 Fig — Group 1: higher-level similarity with the same species. Group 2: higher-level similarity with multiple species. Group 3: unique sequences with no similarity within most sequences. Class 1: sequences with the same species name exhibiting intraspecific cohesive clustering and interspecific distinct clustering with high posterior probability (0.90–1.00). Class 2: sequences with the same species name that do not exhibit intraspecific cohesive clustering. Class 3: sequences with a different species name exhibiting cohesive clustering. There is only one accession number (*). (DOCX) [file pone.0313505.s007.docx]

**
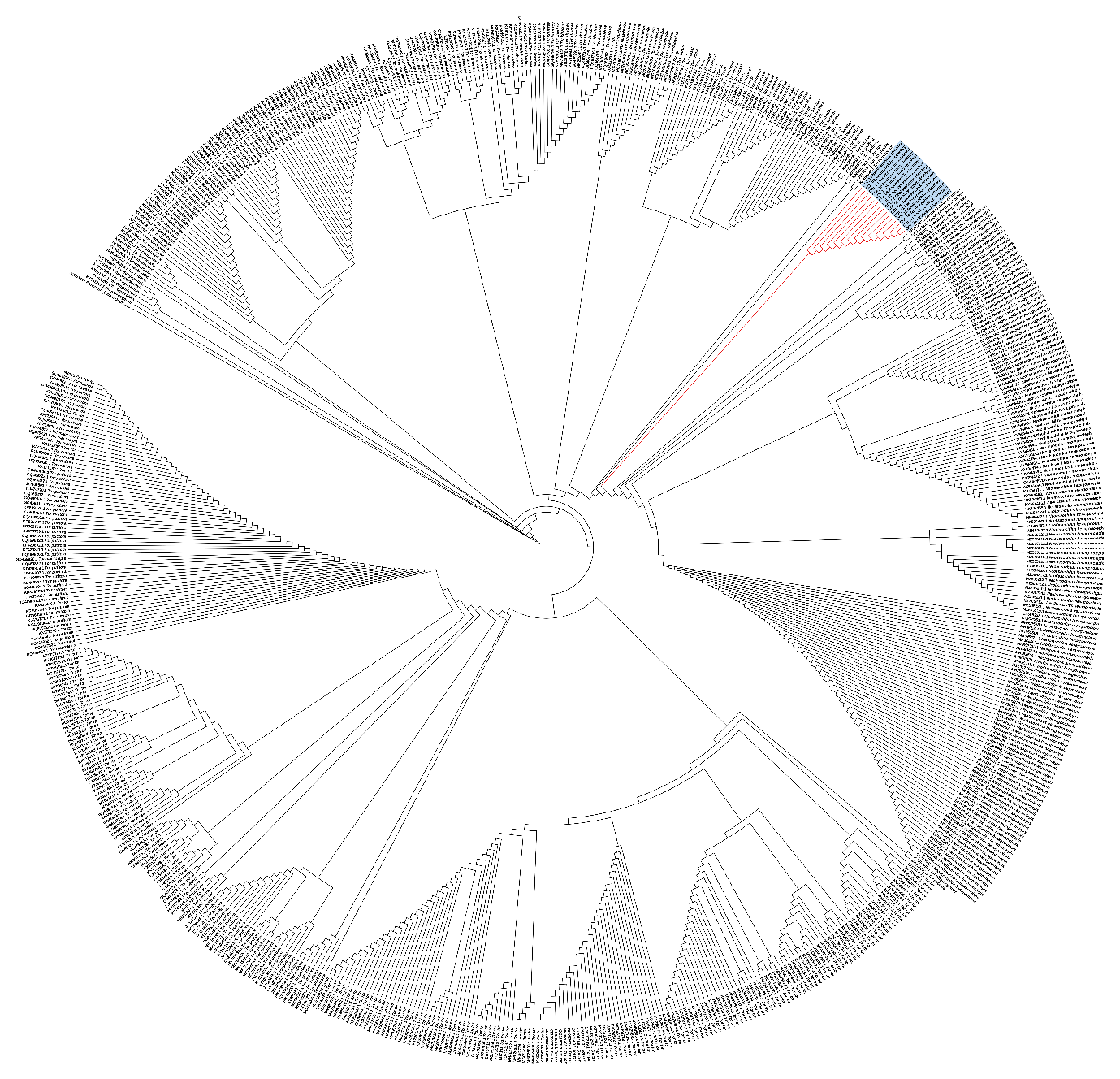
Fig. S8.** (Pongsanarm et al.)

Supplement: S8 Fig — The common Tor douronensis was identified as an outgroup. (DOCX) [file pone.0313505.s008.docx]

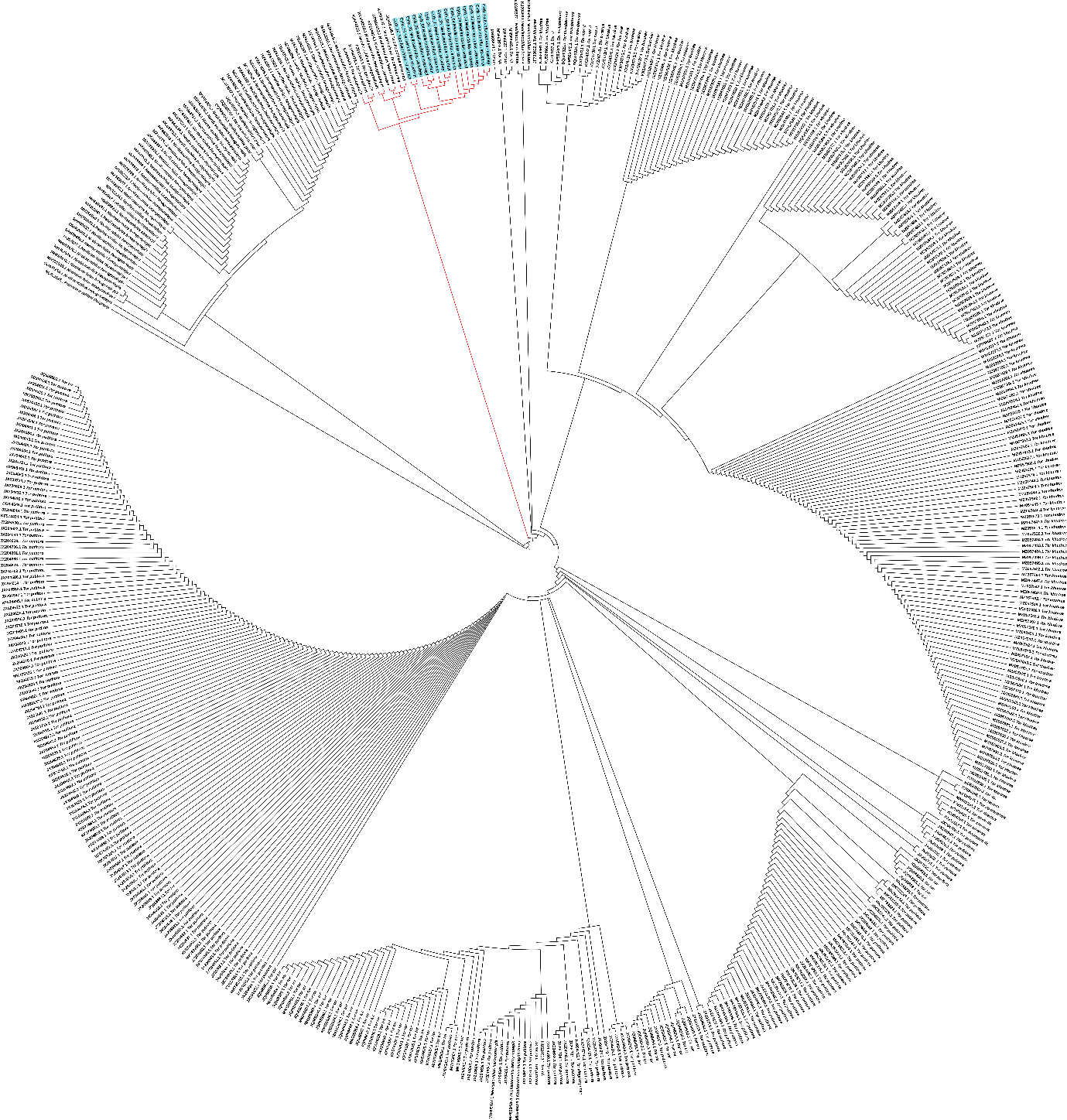


**Fig. S9.** (Pongsanarm et al.)

Supplement: S9 Fig — The common Tor douronensis was identified as an outgroup. (DOCX) [file pone.0313505.s009.docx]

**
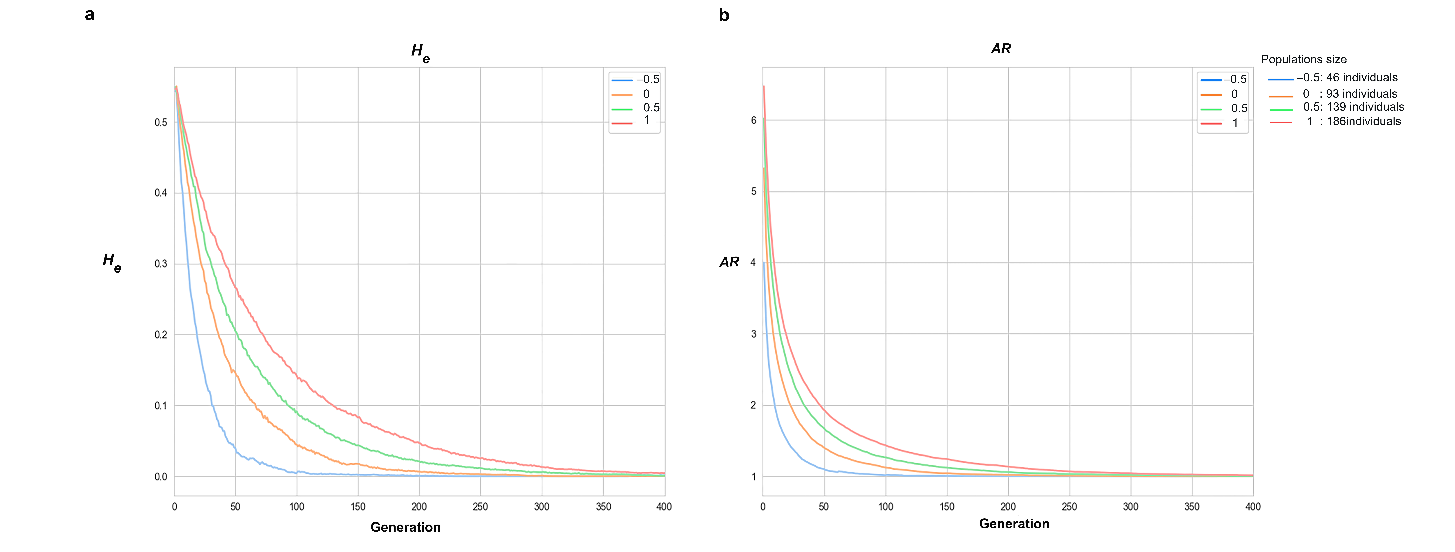
**

**Fig. S10.** (Pongsanarm et al.)

Supplement: S10 Fig — Simulation results showing relationships between generations; (A) heterozygosity, and (B) allelic richness. (DOCX) [file pone.0313505.s010.docx]

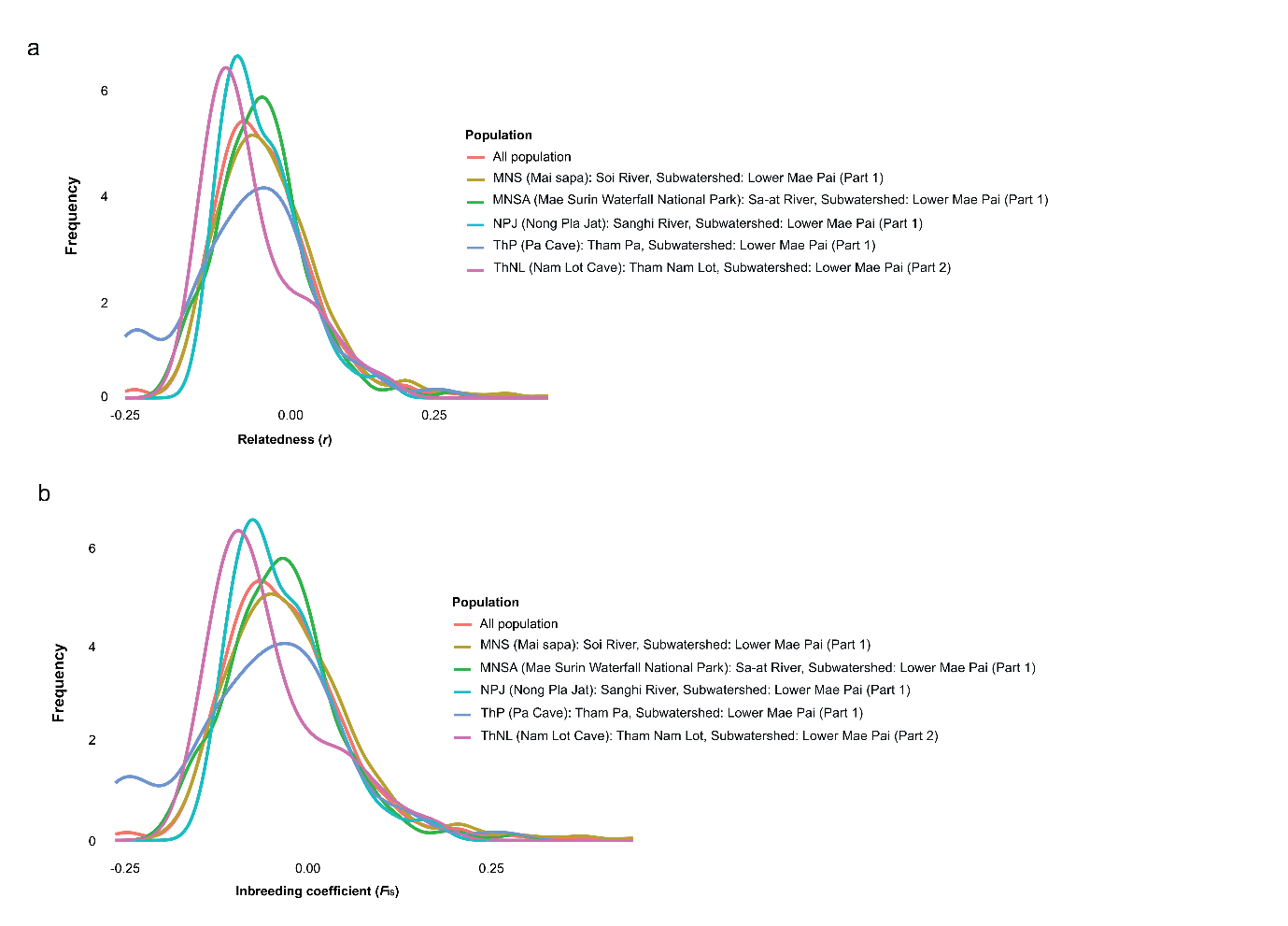


**Fig. S11.** (Pongsanarm et al.)

Supplement: S11 Fig — (A) Observed distribution of inbreeding coefficients (r) in blue mahseer (Neolissochilus stracheyi), plotted against expected distributions. (B) Observed distribution of relatedness (FIS) in blue mahseer, plotted against expected distributions. (DOCX) [file pone.0313505.s011.docx]

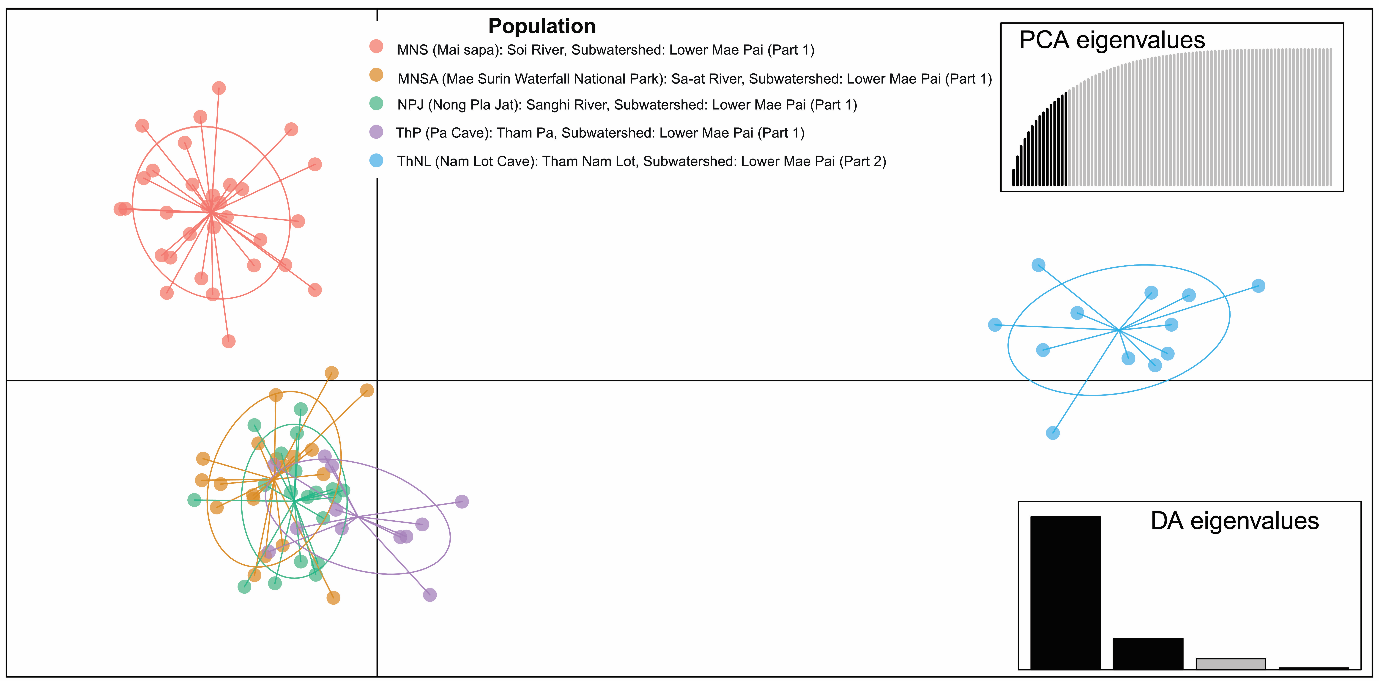


**Fig. S12.** (Pongsanarm et al.)

Supplement: S12 Fig — (DOCX) [file pone.0313505.s012.docx]

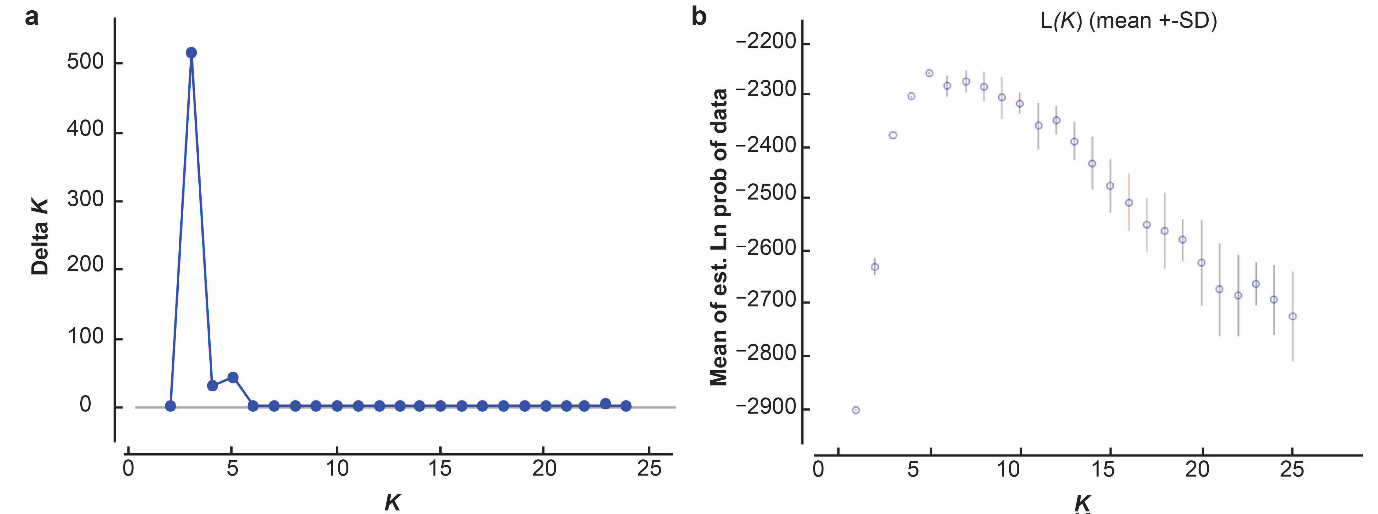


**Fig. S13.** (Pongsanarm et al.)

Supplement: S13 Fig — (A) Plot based on Evano’s ΔK and (B) ln P(K). (DOCX) [file pone.0313505.s013.docx]

**
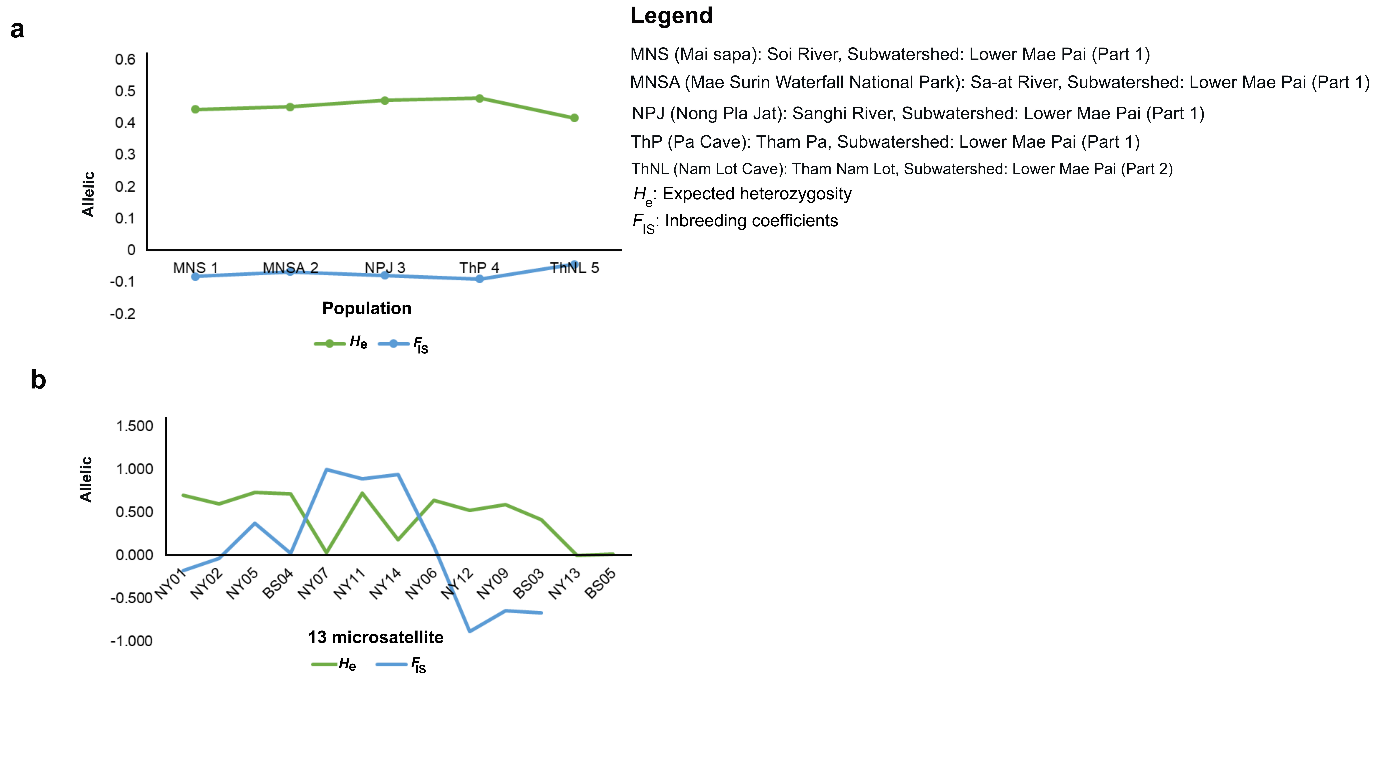
**

**Fig. S14.** (Pongsanarm et al.)

Supplement: S14 Fig — (A) Expected heterozygosity (He). (B) Inbreeding coefficients (FIS). (DOCX) [file pone.0313505.s014.docx]

**
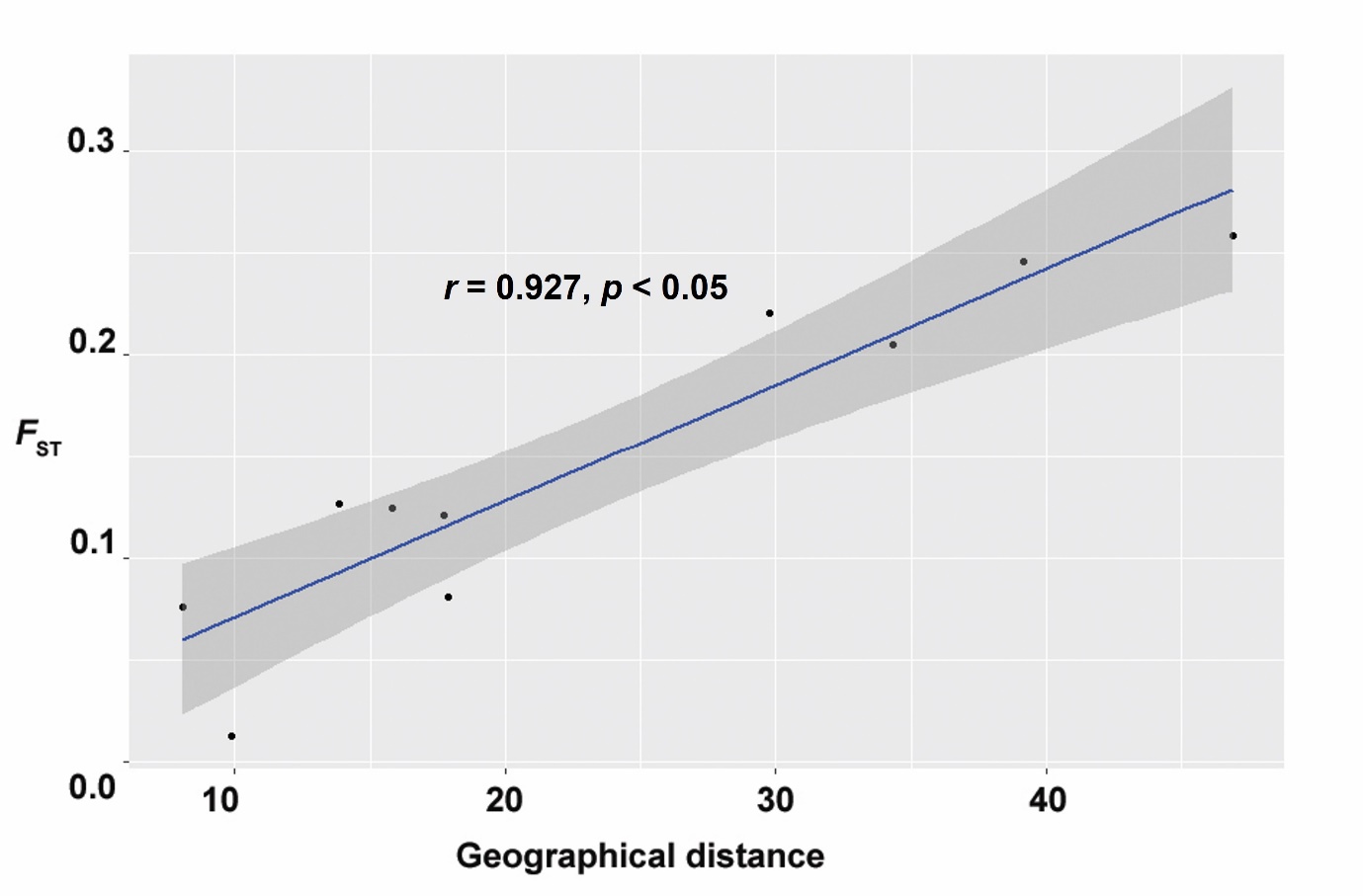
**

**Fig. S15.** (Pongsanarm et al.)

Supplement: S15 Fig — (DOCX) [file pone.0313505.s015.docx]

**
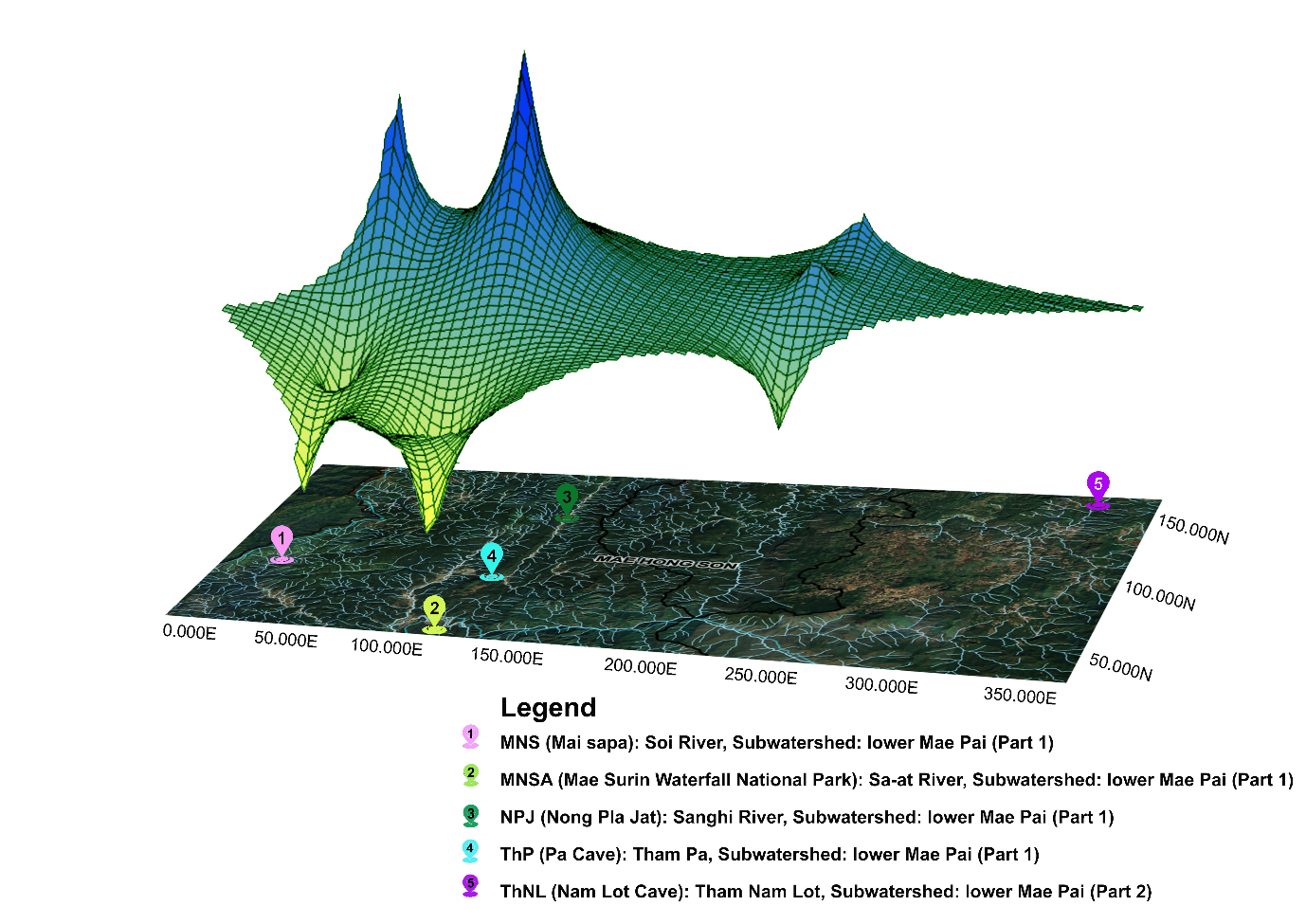
Fig. S16.** (Pongsanarm et al.)

Supplement: S16 Fig — x and y axes correspond to geographic locations within the populations. (DOCX) [file pone.0313505.s016.docx]

**
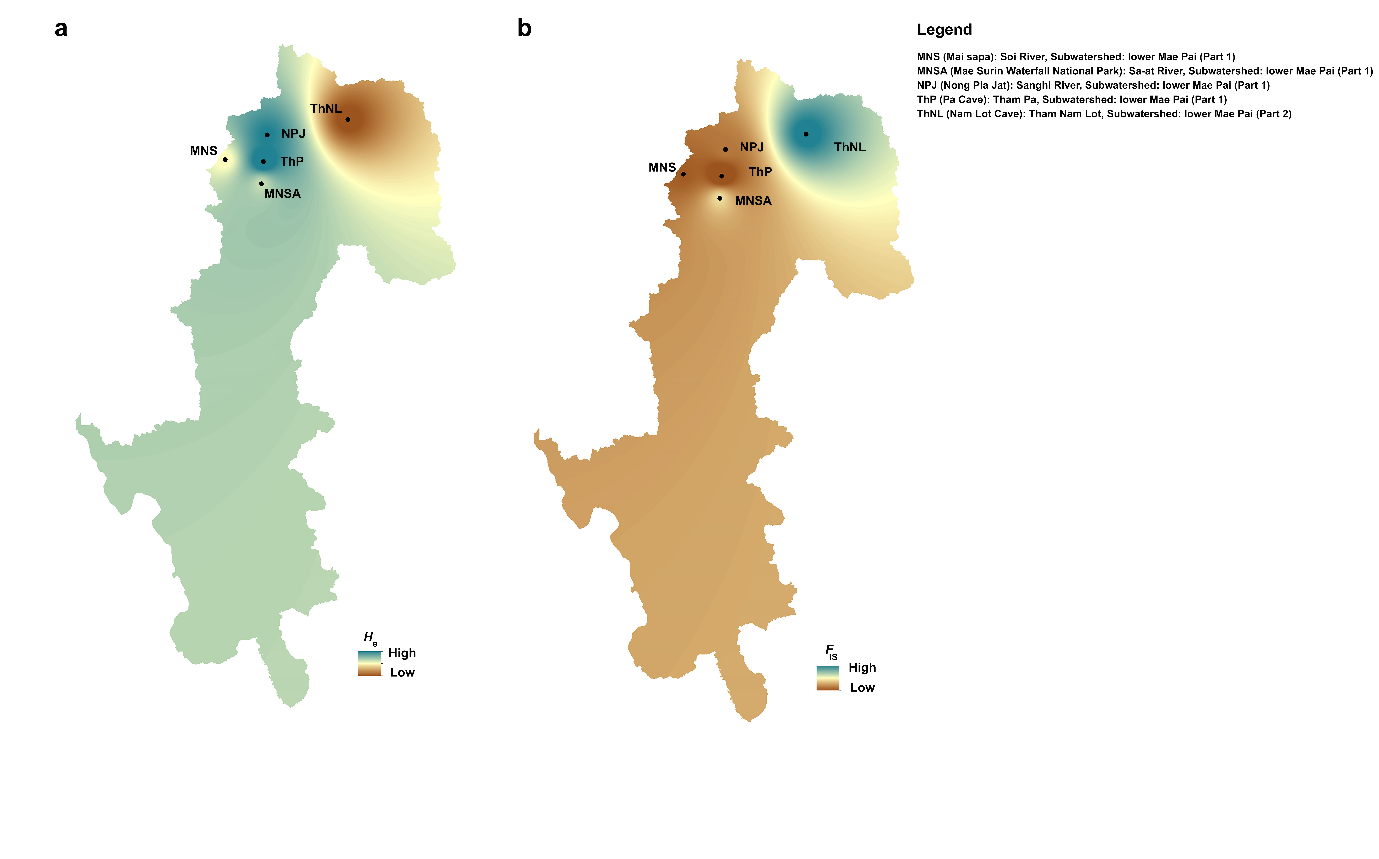
Fig. S17.** (Pongsanarm et al.)

Supplement: S17 Fig — Mapping of expected heterozygosity (He) and inbreeding coefficients (FIS) using QGIS version 3.34.8 (A) He and FIS values in blue mahseer from Soi River (Mai Sapa), Sa-at River (Mae Surin Waterfall National Park), Sanghi River (Nong Pla Jat), Tham Pa (Pa Cave), and Tham Nam Lot (Nam Lot Cave) populations. (B) He and FIS values at 13 microsatellite loci. (DOCX) [file pone.0313505.s017.docx]

**
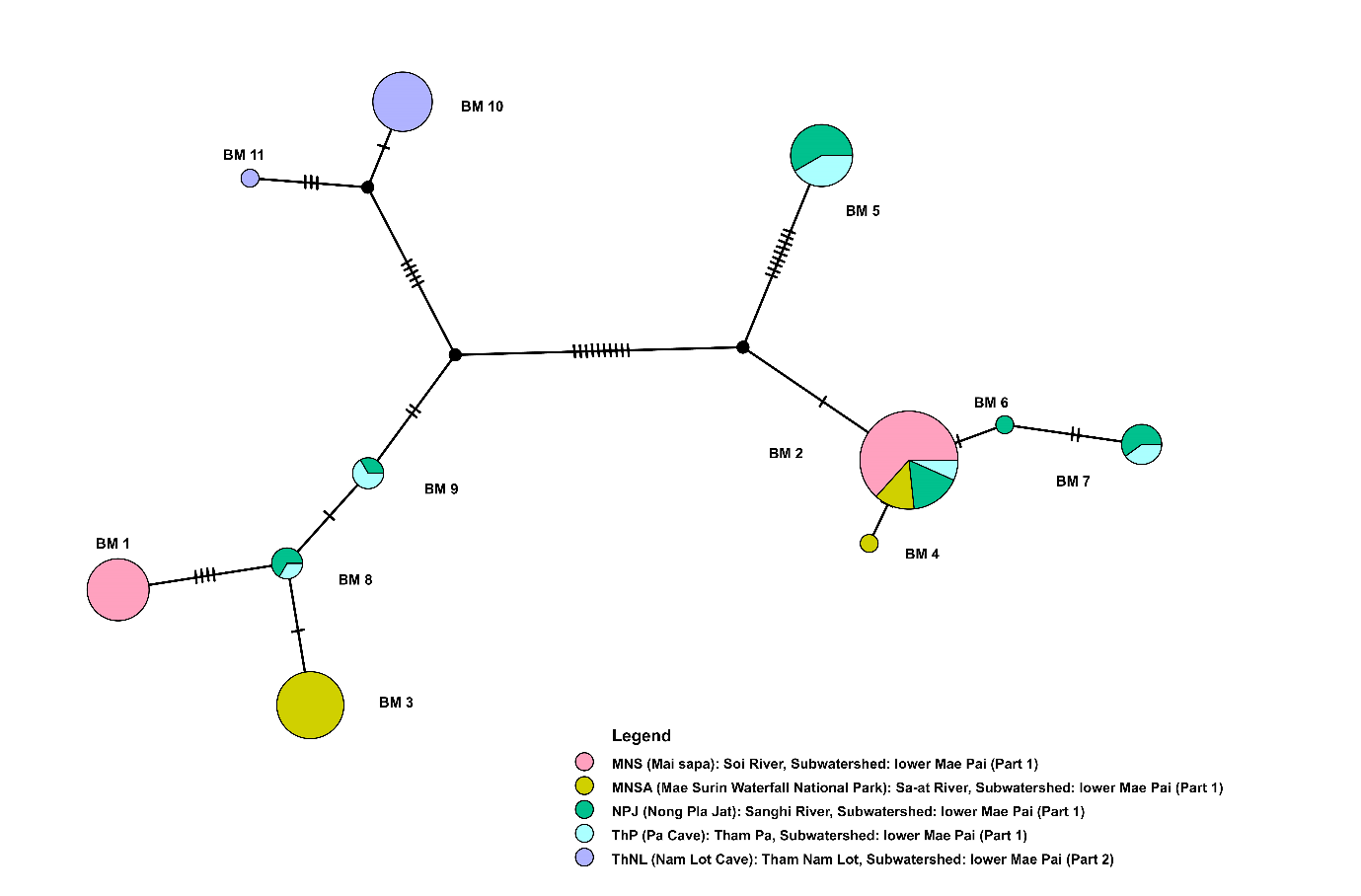
Fig. S18.** (Pongsanarm et al.)

Supplement: S18 Fig — (DOCX) [file pone.0313505.s018.docx]

**
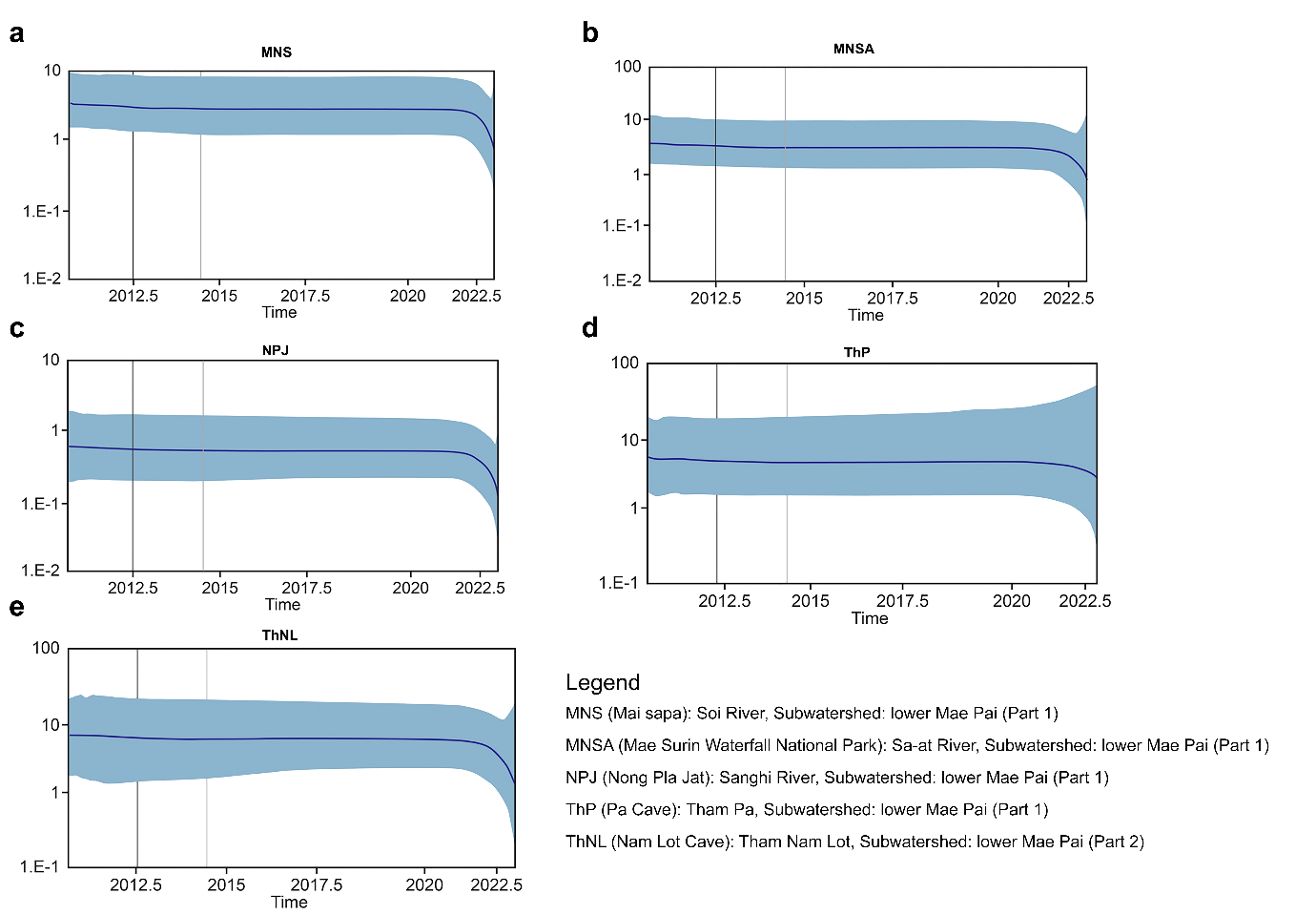
Fig. S19.** (Pongsanarm et al.)

Supplement: S19 Fig — The median effective population size is delimited by the black lines. The blue shaded area delimits the upper and lower bounds of the 95% highest posterior density interval. The x-axis represents time in years and y-axis is displayed in logarithmic scale. (DOCX) [file pone.0313505.s019.docx]

**
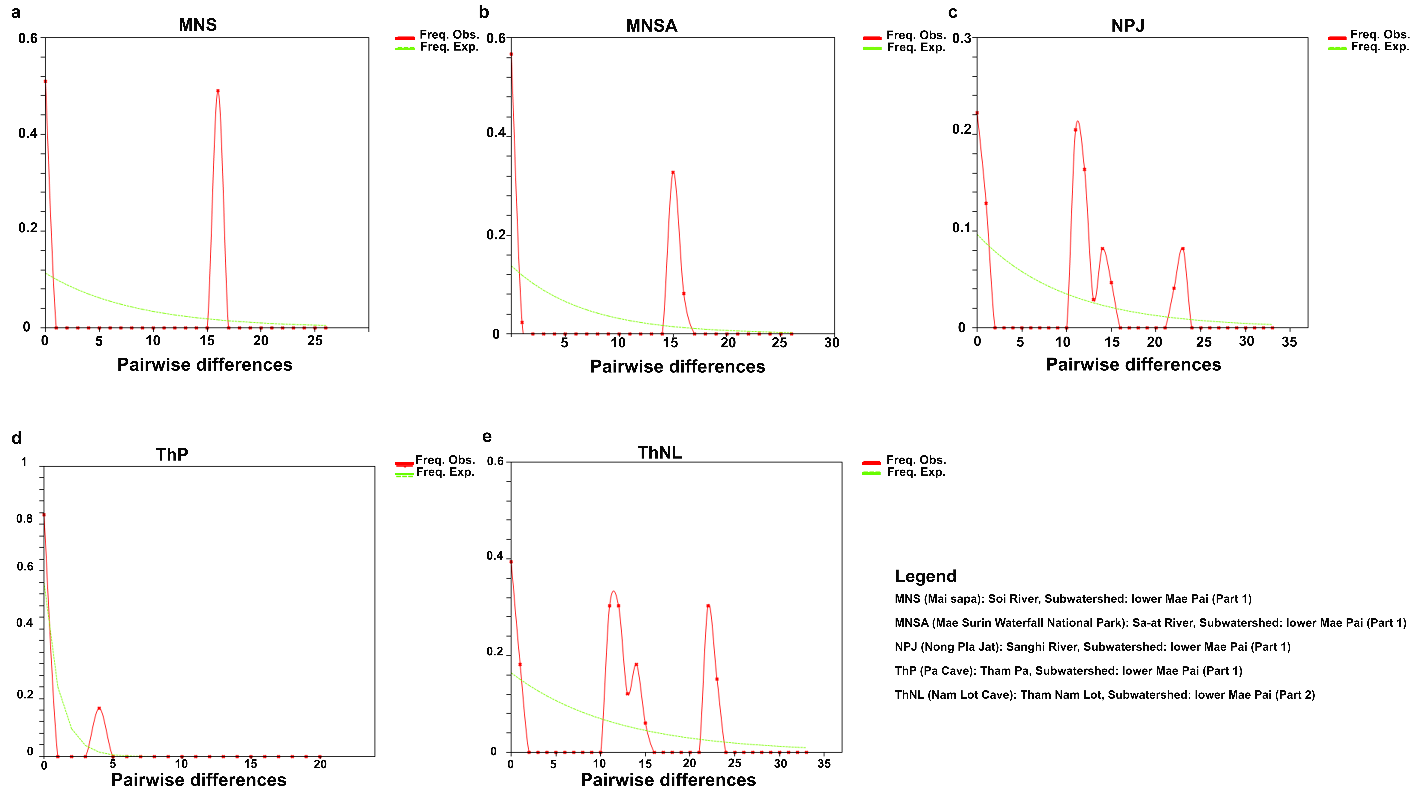
Fig. S20.** (Pongsanarm et al.)

Supplement: S20 Fig — (A) Soi River (Mai sapa), (B) Sa-at River (Mae Surin Waterfall National Park), (C) Sanghi River (Nong Pla Jat), (D) Tham Pa (Pa Cave), and (E) Tham Nam Lot (Nam Lot Cave) population. The x-axis represents the number of pairwise differences (mismatches) and the y-axis represents the frequency of these differences. The frequency distribution of the observed mismatches (red line) is compared to that of the expected mismatches (green line). (DOCX) [file pone.0313505.s020.docx]

**
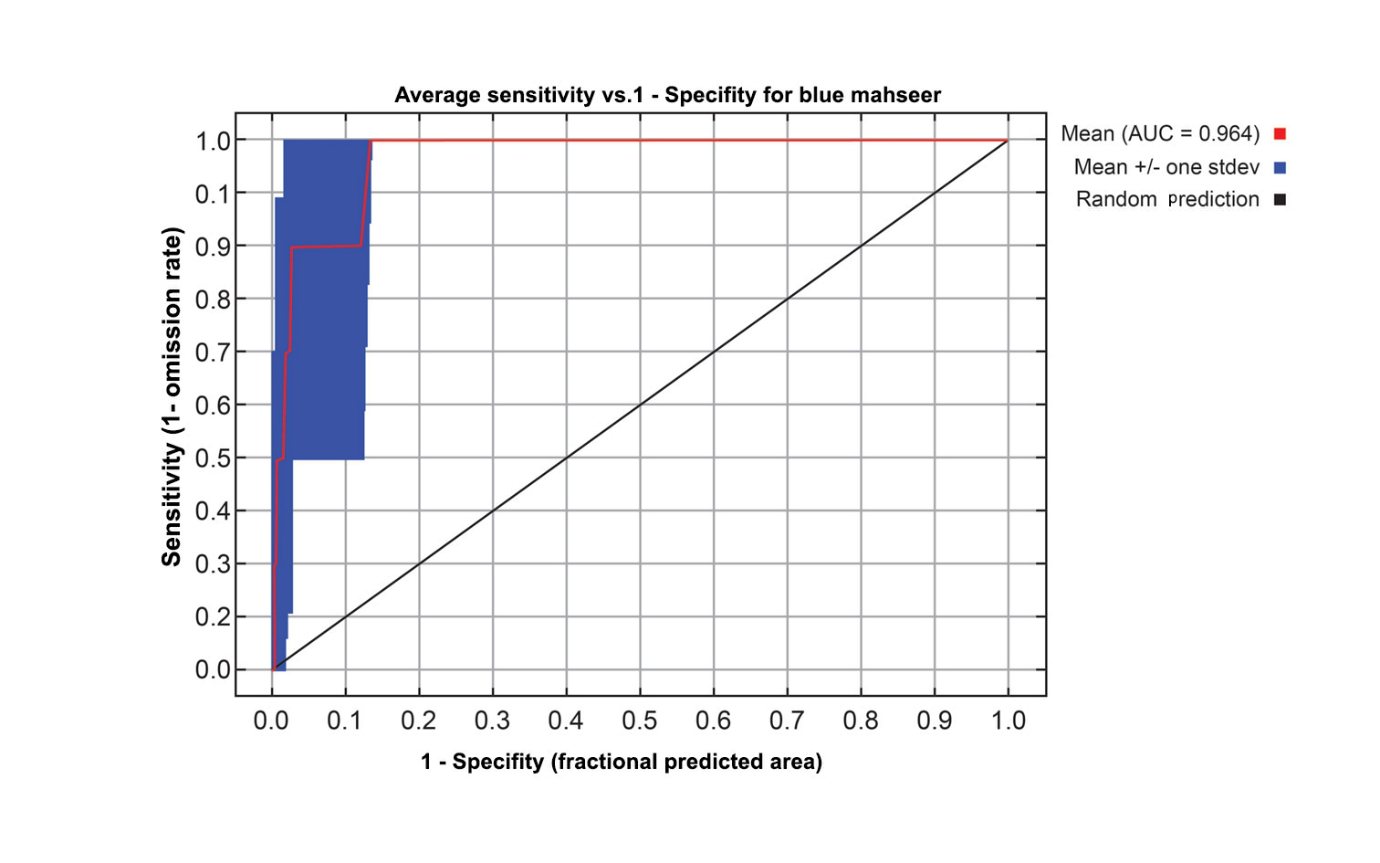
Fig. S21.** (Pongsanarm et al.)

Supplement: S21 Fig — Area under curve (AUC) between average model sensitivity and specificity. (DOCX) [file pone.0313505.s021.docx]

**
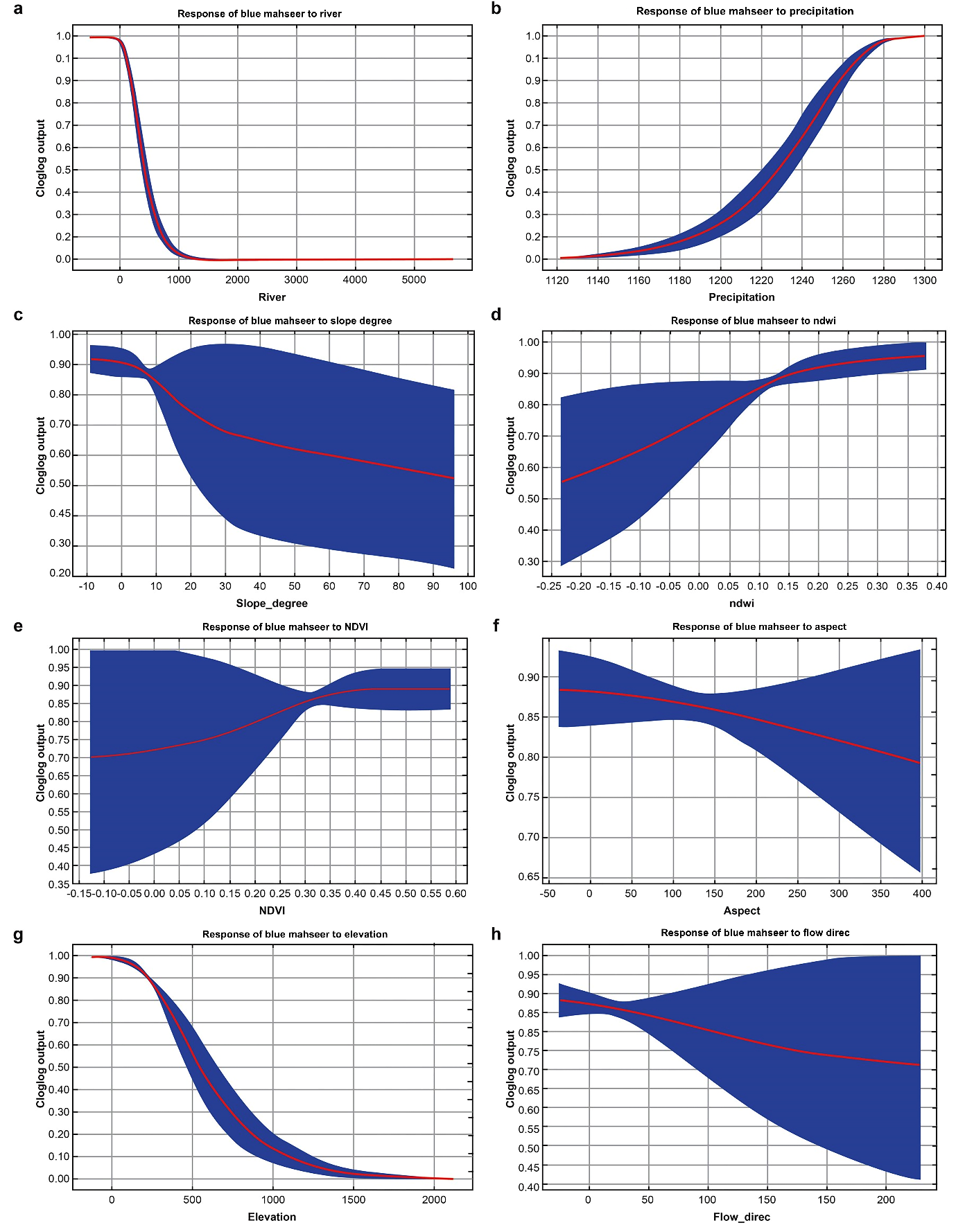
Fig. S22.** (Pongsanarm et al.)

Supplement: S22 Fig — The x‑axes represent the prediction probabilities between 0 (absent) and 1 (100% present). Distance to Rivers (51.7%), annual mean total precipitation (40.8%), slope (3.7%), normalized difference water index (NDWI, 1.1%), normalized difference vegetation index (NDVI, 0.9%), aspect (0.8%), elevation (0.4%), and flow directions (0.4%). (DOCX) [file pone.0313505.s022.docx]

**
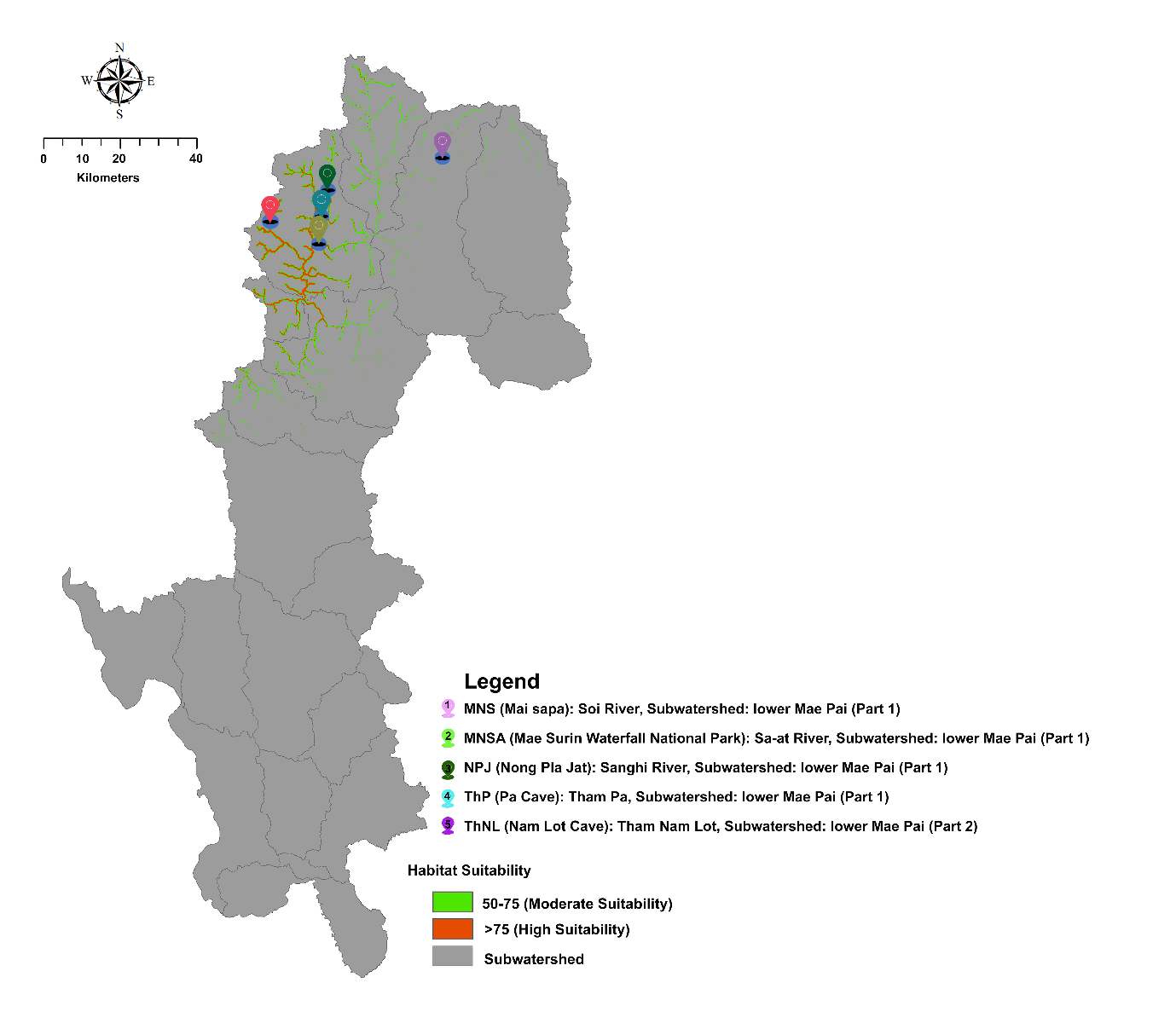
Fig. S23.** (Pongsanarm et al.)

Supplement: S23 Fig — (DOCX) [file pone.0313505.s023.docx]

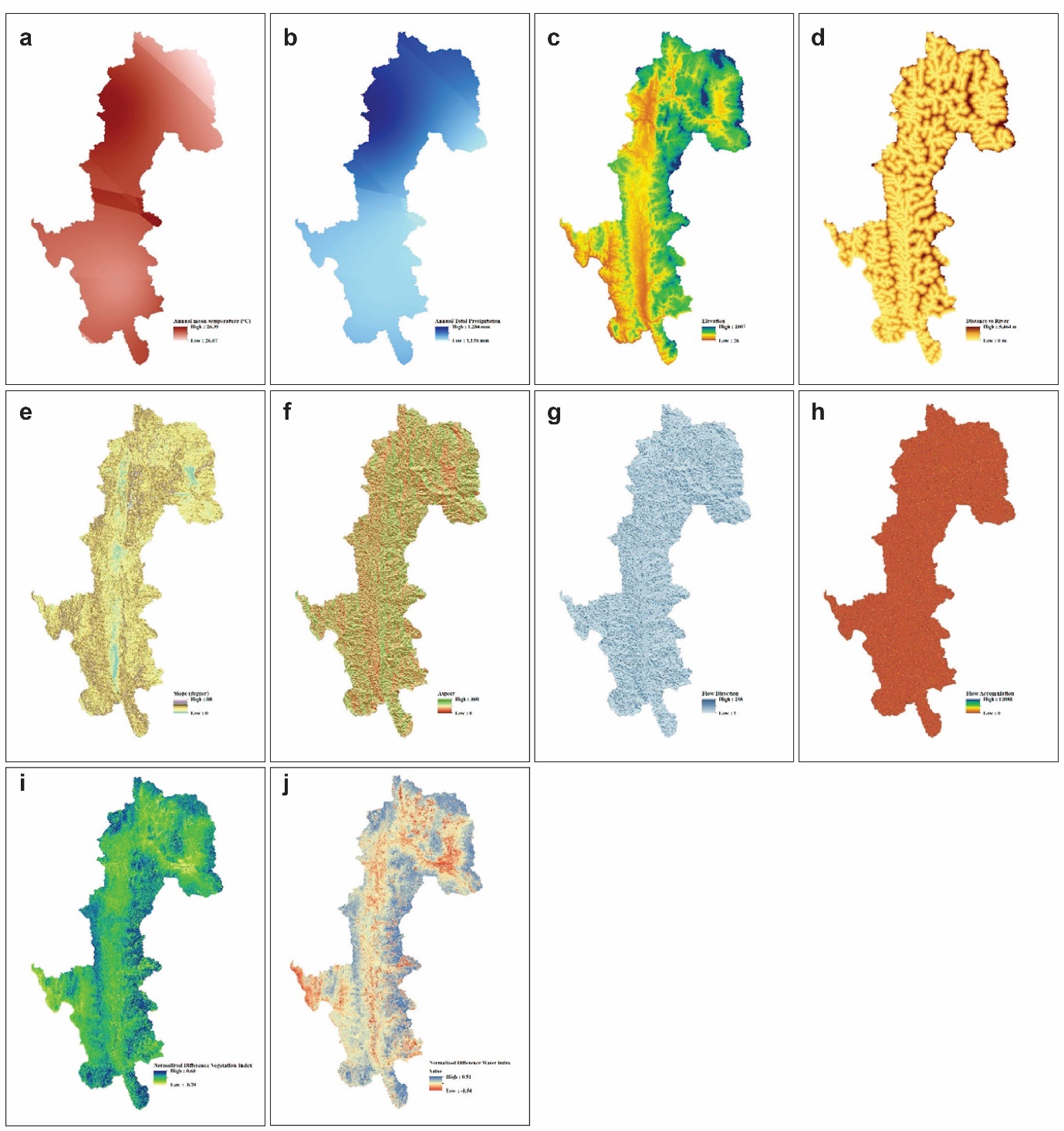


**Fig. S24.** (Pongsanarm et al.)

Supplement: S24 Fig — These include: (A) annual mean temperature (°C), (B) annual total precipitation (mm), (C) elevation, (D) distance to rivers, (E) slope, (F) aspect, (G) flow direction, and (H) flow accumulation. The data for variables (A–H) were obtained from the Land Development Department (2021) and are free for use with no restrictions. Additionally, (I) NDWI and (J) NDVI were derived from Landsat 8 satellite images taken from January to April 2023. These images were accessed via the USGS Earth Explorer (2023), and Landsat data is freely available for use. More information can be found at: https://www.usgs.gov/landsat-missions/landsat-8. (DOCX) [file pone.0313505.s024.docx]
