# Supplementary material for: Genetic and geographical insights call for early conservation of Mae Hong Son’s blue mahseer to prevent population crisis
Source: PLoS One. 2025 Feb 12;20(2):e0313505. doi: 10.1371/journal.pone.0313505 (PMC12143899; doi:10.1371/journal.pone.0313505)
Supplement: S1 File — (DOCX) [file pone.0313505.s025.docx]

**S1 Table.** Summary of blue mahseer (*Neolissochilus stracheyi*) individuals sampled in this study*.*

| **No.** | **Abbreviation/Code** | **Coordinate** | **Locality** |
| --- | --- | --- | --- |
| 1 | MNS1 | 19°25'53.6"N 97°51'18.0"E | Mae Nam Soi |
| 2 | MNS2 | 19°25'53.6"N 97°51'18.0"E | Mae Nam Soi |
| 3 | MNS3 | 19°25'53.6"N 97°51'18.0"E | Mae Nam Soi |
| 4 | MNS4 | 19°25'53.6"N 97°51'18.0"E | Mae Nam Soi |
| 5 | MNS5 | 19°25'53.6"N 97°51'18.0"E | Mae Nam Soi |
| 6 | MNS6 | 19°25'53.6"N 97°51'18.0"E | Mae Nam Soi |
| 7 | MNS7 | 19°25'53.6"N 97°51'18.0"E | Mae Nam Soi |
| 8 | MNS8 | 19°25'53.6"N 97°51'18.0"E | Mae Nam Soi |
| 9 | MNS9 | 19°25'53.6"N 97°51'18.0"E | Mae Nam Soi |
| 10 | MNS10 | 19°25'53.6"N 97°51'18.0"E | Mae Nam Soi |
| 11 | MNS11 | 19°25'53.6"N 97°51'18.0"E | Mae Nam Soi |
| 12 | MNS12 | 19°25'53.6"N 97°51'18.0"E | Mae Nam Soi |
| 13 | MNS13 | 19°25'53.6"N 97°51'18.0"E | Mae Nam Soi |
| 14 | MNS14 | 19°25'53.6"N 97°51'18.0"E | Mae Nam Soi |
| 15 | MNS15 | 19°25'53.6"N 97°51'18.0"E | Mae Nam Soi |
| 16 | MNS16 | 19°25'53.6"N 97°51'18.0"E | Mae Nam Soi |
| 17 | MNS17 | 19°25'53.6"N 97°51'18.0"E | Mae Nam Soi |
| 18 | MNS18 | 19°25'53.6"N 97°51'18.0"E | Mae Nam Soi |
| 19 | MNS19 | 19°25'53.6"N 97°51'18.0"E | Mae Nam Soi |
| 20 | MNS20 | 19°25'53.6"N 97°51'18.0"E | Mae Nam Soi |
| 21 | MNS21 | 19°25'53.6"N 97°51'18.0"E | Mae Nam Soi |
| 22 | MNS22 | 19°25'53.6"N 97°51'18.0"E | Mae Nam Soi |
| 23 | MNS23 | 19°25'53.6"N 97°51'18.0"E | Mae Nam Soi |
| 24 | MNS24 | 19°25'53.6"N 97°51'18.0"E | Mae Nam Soi |
| 25 | MNS25 | 19°25'53.6"N 97°51'18.0"E | Mae Nam Soi |
| 26 | MNS26 | 19°25'53.6"N 97°51'18.0"E | Mae Nam Soi |
| 27 | MNS27 | 19°25'53.6"N 97°51'18.0"E | Mae Nam Soi |
| 28 | MNS28 | 19°25'53.6"N 97°51'18.0"E | Mae Nam Soi |
| 29 | MNS29 | 19°25'53.6"N 97°51'18.0"E | Mae Nam Soi |
| 30 | MNS30 | 19°25'53.6"N 97°51'18.0"E | Mae Nam Soi |

| **No.** | **Abbreviation/Code** | **Coordinate** | **Locality** |
| --- | --- | --- | --- |
| 31 | MNS31 | 19°25'53.6"N 97°51'18.0"E | Mae Nam Soi |
| 32 | MNSA1 | 19°21'12.9"N 97°58'51.9"E | Mae Nam Sa-At |
| 33 | MNSA2 | 19°21'12.9"N 97°58'51.9"E | Mae Nam Sa-At |
| 34 | MNSA3 | 19°21'12.9"N 97°58'51.9"E | Mae Nam Sa-At |
| 35 | MNSA4 | 19°21'12.9"N 97°58'51.9"E | Mae Nam Sa-At |
| 36 | MNSA5 | 19°21'12.9"N 97°58'51.9"E | Mae Nam Sa-At |
| 37 | MNSA6 | 19°21'12.9"N 97°58'51.9"E | Mae Nam Sa-At |
| 38 | MNSA7 | 19°21'12.9"N 97°58'51.9"E | Mae Nam Sa-At |
| 39 | MNSA8 | 19°21'12.9"N 97°58'51.9"E | Mae Nam Sa-At |
| 40 | MNSA9 | 19°21'12.9"N 97°58'51.9"E | Mae Nam Sa-At |
| 41 | MNSA10 | 19°21'12.9"N 97°58'51.9"E | Mae Nam Sa-At |
| 42 | MNSA11 | 19°21'12.9"N 97°58'51.9"E | Mae Nam Sa-At |
| 43 | MNSA12 | 19°21'12.9"N 97°58'51.9"E | Mae Nam Sa-At |
| 44 | MNSA13 | 19°21'12.9"N 97°58'51.9"E | Mae Nam Sa-At |
| 45 | MNSA14 | 19°21'12.9"N 97°58'51.9"E | Mae Nam Sa-At |
| 46 | MNSA15 | 19°21'12.9"N 97°58'51.9"E | Mae Nam Sa-At |
| 47 | MNSA16 | 19°21'12.9"N 97°58'51.9"E | Mae Nam Sa-At |
| 48 | MNSA17 | 19°21'12.9"N 97°58'51.9"E | Mae Nam Sa-At |
| 49 | MNSA18 | 19°21'12.9"N 97°58'51.9"E | Mae Nam Sa-At |
| 50 | MNSA19 | 19°21'12.9"N 97°58'51.9"E | Mae Nam Sa-At |
| 51 | NPJ1 | 19°30'48.2"N 98°00'01.1"E | Nong Pla Jat |
| 52 | NPJ2 | 19°30'48.2"N 98°00'01.1"E | Nong Pla Jat |
| 53 | NPJ3 | 19°30'48.2"N 98°00'01.1"E | Nong Pla Jat |
| 54 | NPJ4 | 19°30'48.2"N 98°00'01.1"E | Nong Pla Jat |
| 55 | NPJ5 | 19°30'48.2"N 98°00'01.1"E | Nong Pla Jat |
| 56 | NPJ6 | 19°30'48.2"N 98°00'01.1"E | Nong Pla Jat |
| 57 | NPJ7 | 19°30'48.2"N 98°00'01.1"E | Nong Pla Jat |
| 58 | NPJ8 | 19°30'48.2"N 98°00'01.1"E | Nong Pla Jat |
| 59 | NPJ9 | 19°30'48.2"N 98°00'01.1"E | Nong Pla Jat |
| 60 | NPJ10 | 19°30'48.2"N 98°00'01.1"E | Nong Pla Jat |
| 61 | NPJ11 | 19°30'48.2"N 98°00'01.1"E | Nong Pla Jat |
| 62 | NPJ12 | 19°30'48.2"N 98°00'01.1"E | Nong Pla Jat |
| 63 | NPJ13 | 19°30'48.2"N 98°00'01.1"E | Nong Pla Jat |
| 64 | NPJ14 | 19°30'48.2"N 98°00'01.1"E | Nong Pla Jat |
| 65 | NPJ15 | 19°30'48.2"N 98°00'01.1"E | Nong Pla Jat |

| **No.** | **Abbreviation/Code** | **Coordinate** | **Locality** |
| --- | --- | --- | --- |
| 66 | NPJ16 | 19°30'48.2"N 98°00'01.1"E | Nong Pla Jat |
| 67 | NPJ17 | 19°30'48.2"N 98°00'01.1"E | Nong Pla Jat |
| 68 | NPJ18 | 19°30'48.2"N 98°00'01.1"E | Nong Pla Jat |
| 69 | NPJ19 | 19°30'48.2"N 98°00'01.1"E | Nong Pla Jat |
| 70 | ThP1 | 19°25'32.6"N 97°59'13.5"E | Tham Pla |
| 71 | ThP2 | 19°25'32.6"N 97°59'13.5"E | Tham Pla |
| 72 | ThP3 | 19°25'32.6"N 97°59'13.5"E | Tham Pla |
| 73 | ThP4 | 19°25'32.6"N 97°59'13.5"E | Tham Pla |
| 74 | ThP5 | 19°25'32.6"N 97°59'13.5"E | Tham Pla |
| 75 | ThP6 | 19°25'32.6"N 97°59'13.5"E | Tham Pla |
| 76 | ThP7 | 19°25'32.6"N 97°59'13.5"E | Tham Pla |
| 77 | ThP8 | 19°25'32.6"N 97°59'13.5"E | Tham Pla |
| 78 | ThP9 | 19°25'32.6"N 97°59'13.5"E | Tham Pla |
| 79 | ThP10 | 19°25'32.6"N 97°59'13.5"E | Tham Pla |
| 80 | ThP11 | 19°25'32.6"N 97°59'13.5"E | Tham Pla |
| 81 | ThP12 | 19°25'32.6"N 97°59'13.5"E | Tham Pla |
| 82 | ThNL1 | 19°33'55.7"N 98°16'45.0"E | Tham Nam Lot |
| 83 | ThNL2 | 19°33'55.7"N 98°16'45.0"E | Tham Nam Lot |
| 84 | ThNL3 | 19°33'55.7"N 98°16'45.0"E | Tham Nam Lot |
| 85 | ThNL4 | 19°33'55.7"N 98°16'45.0"E | Tham Nam Lot |
| 86 | ThNL5 | 19°33'55.7"N 98°16'45.0"E | Tham Nam Lot |
| 87 | ThNL6 | 19°33'55.7"N 98°16'45.0"E | Tham Nam Lot |
| 88 | ThNL7 | 19°33'55.7"N 98°16'45.0"E | Tham Nam Lot |
| 89 | ThNL8 | 19°33'55.7"N 98°16'45.0"E | Tham Nam Lot |
| 90 | ThNL9 | 19°33'55.7"N 98°16'45.0"E | Tham Nam Lot |
| 91 | ThNL10 | 19°33'55.7"N 98°16'45.0"E | Tham Nam Lot |
| 92 | ThNL11 | 19°33'55.7"N 98°16'45.0"E | Tham Nam Lot |
| 93 | ThNL12 | 19°33'55.7"N 98°16'45.0"E | Tham Nam Lot |

**S2 Table.** Microsatellite primers and sequences used in this study

| **Primer** | **Fluorescence** | **Primer sequence 5′ to 3′** | | **Size (bp)** |
| --- | --- | --- | --- | --- |
|  |  | **Reverse** | **Forward** |  |
| **NY01** | FAM | GGACTGACACTGGGGATCAT | CCGAAATGCATTCTTGTCTT | 223–243 |
| **NY02** | HEX | GCTTCCCCTCATAAGCCTTC | GAGGGGCATTTTGTTCTTGA | 238–274 |
| **NY05** | TAMRA | TGATGCCTGTCAAACCTGTG | GCTGAAGCAGGTGAATCTGA | 168–208 |
| **BS04** | FAM | GTTGGCTCTCCGTTCTTCAG | CCAAACTGTCCATGCTGTCC | 131-173 |
| **NY07** | HEX | AACCTGTGGGGATGTCCAG | CATGGACCAAATTACAAGGATTT | 233–245 |
| **NY11** | TAMRA | TGGGAGATGTTGTTTCTCCA | CCATTACGCCTTTGGAGTGT | 239–249 |
| **NY14** | FAM | GCCAGGATGATGAGCATGTA | GGCTGTGAATGTGTTTGTGG | 201–283 |
| **NY06** | FAM | TTCAGCTCAGAGGGGACACT | GAGTCCCTACAGACGTATTTCCA | 151–173 |
| **NY12** | HEX | CGTGTGTATGATGCCACCTC | ATGCCAGCTACAGGTCCAAT | 150–162 |
| **NY09** | HEX | TATGTGGTTTCAGGCAGCAG | TGGAAATTGAGACAAAGCTTCA | 183–197 |
| **BS03** | FAM | AAAGGCAATCGGAATGCAGC | CTCTTGCTTCTCGAGGGACT | 208–224 |
| **NY13** | HEX | GTGTGGCCAGTGTAGCTGAA | GAGACGACTCTAGTCGCTGACA | 140–174 |
| **BS05** | FAM | AGCCATGAATCAAAGACGCC | CTCTTGCTTCTCGAGGGAC | 241–259 |

**S3 Table**. Comparison of Kimura’s two-parameter (K2P) sequence divergence between individual sequences from two resource for comparison.

| Species | Common name | *COI* K2P | | | | *Cytb* K2P | | | | |
| --- | --- | --- | --- | --- | --- | --- | --- | --- | --- | --- |
|  |  | **Minimum**  **intraspecific** | **Maximum**  **intraspecific** | **Minimum**  **interspecific** | **Maximum**  **interspecific** | **Minimum**  **intraspecific** | **Maximum**  **intraspecific** | **Minimum**  **interspecific** | Maximum  interspecific |  |
| *T. khudree* | Deccan mahseer | 0.000 | 0.000 | 0.031 | 0.031 | 0.000 | 0.000 | 0.034 | 0.034 |  |
| *T. malabaricus* | Malabar mahseer | 0.000 | 0.000 | 0.031 | 0.031 | 0.000 | 0.000 | 0.045 | 0.045 |  |
| *T. douronensis* | Semah mahseer | 0.000 | 0.000 | 0.016 | 0.016 | 0.000 | 0.000 | 0.000 | 0.000 |  |
| *T. tambroides* | Thai mahseer | 0.000 | 0.000 | 0.016 | 0.016 | ‒ | ‒ | ‒ | ‒ |  |
| *T. sinensis* | Red mahseer | 0.000 | 0.000 | 0.019 | 0.019 | 0.000 | 0.000 | 0.000 | 0.000 |  |
| *T. putitora* | Himalayan mahseer | 0.000 | 0.000 | 0.012 | 0.012 | 0.000 | 0.000 | 0.008 | 0.008 |  |
| *T. tor* | Tor barb | 0.000 | 0.000 | 0.016 | 0.016 | 0.000 | 0.000 | 0.003 | 0.003 |  |
| *T. mosal* | Mahanadi mahseer | 0.000 | 0.000 | 0.005 | 0.005 | 0.000 | 0.000 | 0.010 | 0.010 |  |
| *T. macrolepis* | Indus mahseer | 0.000 | 0.000 | 0.005 | 0.005 | ‒ | ‒ | ‒ | ‒ |  |
| *T. mussullah* | Mussullah barb | 0.000 | 0.000 | 0.031 | 0.031 | ‒ | ‒ | ‒ | ‒ |  |
| *T. barakae* | Barak mahseer | 0.000 | 0.000 | 0.019 | 0.019 | 0.000 | 0.000 | 0.010 | 0.010 |  |
| *T. remadeviae* | Hump-backed mahseer | 0.000 | 0.000 | 0.016 | 0.016 | ‒ | ‒ | ‒ | ‒ |  |
| *T. qiaojiensis* | - | ‒ | ‒ | ‒ | ‒ | 0.000 | 0.000 | 0.051 | 0.051 |  |
| *N. stracheyi* | Blue mahseer | 0.000 | 0.000 | 0.076 | 0.076 | 0.000 | 0.000 | 0.000 | 0.000 |  |
| *N. hexagonolepis* | Copper mahseer | 0.000 | 0.000 | 0.076 | 0.076 | 0.000 | 0.000 | 0.003 | 0.003 |  |
| *N. pnar* | ‒ | ‒ | ‒ | ‒ | ‒ | 0.000 | 0.000 | 0.000 | 0.000 |  |
| *N. benasi* | ‒ | ‒ | ‒ | ‒ | ‒ | 0.000 | 0.000 | 0.068 | 0.068 |  |

**S4 Table**. Substitution saturation analyses of Cytochrome c oxidase I (*COI*) and Cytochrome b (*Cytb*) are based on the index of substitution saturation as implemented in DAMBE.

| Regions | Number of OTUa | Issb | Iss.cSymc | Dfd | *p* valuee | Iss.cAsymf | df | *p-*value |
| --- | --- | --- | --- | --- | --- | --- | --- | --- |
| *COI* | 4 | 0.356 | 0.801 | 589 | 0.0000 | 0.769 | 589 | 0.0000 |
|  | 8 | 0.372 | 0.760 | 589 | 0.0000 | 0.649 | 589 | 0.0000 |
|  | 16 | 0.387 | 0.736 | 589 | 0.0000 | 0.525 | 589 | 0.0000 |
|  | 32 | 0.405 | 0.711 | 589 | 0.0000 | 0.383 | 589 | 0.5689 |
| *Cytb* | 4 | 0.060 | 0.790 | 420 | 0.0000 | 0.758 | 420 | 0.0000 |
|  | 8 | 0.059 | 0.744 | 420 | 0.0000 | 0.633 | 420 | 0.0000 |
|  | 16 | 0.064 | 0.706 | 420 | 0.0000 | 0.497 | 420 | 0.0000 |
|  | 32 | 0.073 | 0.693 | 420 | 0.0000 | 0.365 | 420 | 0.0000 |

aNumber of sequences used for random resampling; OTU, operational taxonomic unit. bIndex of substitution saturation.
cCritical value for a symmetrical tree topology. dDegrees of freedom.

eProbability that Iss is significantly different from the critical value (Iss.cSym/Iss.cAsym) fCritical value for an asymmetrical tree topology.

**S5 Table**. Specimen populations of 15 blue mahseer (*Neolissochilus stracheyi*) in this study. All sequences for *COI* were deposited in the DNA Data Bank of Japan (DDBJ) and BLASTn of sequence identity (http://blast.ncbi.nlm.nih.gov/Blast.cgi).

| No. | Abbreviation/  Code | Species | Locality | *COI* DDBJ accession number |
| --- | --- | --- | --- | --- |
| 1 | MNS1 | *N. stracheyi* | Soi river (Mai sapa) | LC785353 |
| 2 | MNS 2 | *N. stracheyi* | Soi river (Mai sapa) | LC785353 |
| 3 | MNS 3 | *N. stracheyi* | Soi river (Mai sapa) | LC785353 |
| 4 | MNSA1 | *N. stracheyi* | Sa-at river (Mae surin waterfall National Park) | LC785353 |
| 5 | MNSA2 | *N. stracheyi* | Sa-at river (Mae surin waterfall National Park) | LC785353 |
| 6 | MNSA3 | *N. stracheyi* | Sa-at river (Mae surin waterfall National Park) | LC785353 |
| 7 | NPJ1 | *N. stracheyi* | Sanghi river (Nong Pla Jat) | LC785353 |
| 8 | NPJ2 | *N. stracheyi* | Sanghi river (Nong Pla Jat) | LC785353 |
| 9 | NPJ3 | *N. stracheyi* | Sanghi river (Nong Pla Jat) | LC785353 |
| 10 | ThP1 | *N. stracheyi* | Tham Pa (Pa cave) | LC785353 |
| 11 | ThP2 | *N. stracheyi* | Tham Pa (Pa cave) | LC785353 |
| 12 | ThP3 | *N. stracheyi* | Tham Pa (Pa cave) | LC785353 |
| 13 | ThNL1 | *N. stracheyi* | Tham Nam Lot (Nam Lot cave) | LC785353 |
| 14 | ThNL2 | *N. stracheyi* | Tham Nam Lot (Nam Lot cave) | LC785353 |
| 15 | ThNL3 | *N. stracheyi* | Tham Nam Lot (Nam Lot cave) | LC785353 |

**S6 Table**. Specimen populations of 15 Blue mahseer (*Neolissochilus stracheyi*) in this study. All sequences for *Cytb* were deposited in the DNA Data Bank of Japan (DDBJ) and BLASTn of sequence identity (http://blast.ncbi.nlm.nih.gov/Blast.cgi).

| No. | Abbreviation/  Code | Species | Locality | *Cytb* DDBJ accession number |
| --- | --- | --- | --- | --- |
| 1 | MNS1 | *N. stracheyi* | Soi river (Mai sapa) | LC785347 |
| 2 | MNS 2 | *N. stracheyi* | Soi river (Mai sapa) | LC785347 |
| 3 | MNS 3 | *N. stracheyi* | Soi river (Mai sapa) | LC785347 |
| 4 | MNSA1 | *N. stracheyi* | Sa-at river (Mae surin waterfall National Park) | LC785347 |
| 5 | MNSA2 | *N. stracheyi* | Sa-at river (Mae surin waterfall National Park) | LC785347 |
| 6 | MNSA3 | *N. stracheyi* | Sa-at river (Mae surin waterfall National Park) | LC785347 |
| 7 | NPJ1 | *N. stracheyi* | Sanghi river (Nong Pla Jat) | LC785348 |
| 8 | NPJ2 | *N. stracheyi* | Sanghi river (Nong Pla Jat) | LC785348 |
| 9 | NPJ3 | *N. stracheyi* | Sanghi river (Nong Pla Jat) | LC785348 |
| 10 | ThP1 | *N. stracheyi* | Tham Pa (Pa cave) | LC785349 |
| 11 | ThP2 | *N. stracheyi* | Tham Pa (Pa cave) | LC785349 |
| 12 | ThP3 | *N. stracheyi* | Tham Pa (Pa cave) | LC785350 |
| 13 | ThNL1 | *N. stracheyi* | Tham Nam Lot (Nam Lot cave) | LC785351 |
| 14 | ThNL2 | *N. stracheyi* | Tham Nam Lot (Nam Lot cave) | LC785352 |
| 15 | ThNL3 | *N. stracheyi* | Tham Nam Lot (Nam Lot cave) | LC785353 |

**S7 Table**. Specimen populations of 93 blue mahseer (*Neolissochilus stracheyi*) in this study. All sequences for D-loop were deposited in the DNA Data Bank of Japan (DDBJ) and BLASTn of sequence identity (http://blast.ncbi.nlm.nih.gov/Blast.cgi).

| **No.** | **Abbreviation/Code** | **Species** | **Locality** | **DDBJ accession number** |
| --- | --- | --- | --- | --- |
| 1 | MNS1 | *N. stracheyi* | Soi river (Mai sapa) | LC785336 |
| 2 | MNS2 | *N. stracheyi* | Soi river (Mai sapa) | LC785337 |
| 3 | MNS3 | *N. stracheyi* | Soi river (Mai sapa) | LC785336 |
| 4 | MNS4 | *N. stracheyi* | Soi river (Mai sapa) | LC785336 |
| 5 | MNS5 | *N. stracheyi* | Soi river (Mai sapa) | LC785337 |
| 6 | MNS6 | *N. stracheyi* | Soi river (Mai sapa) | LC785336 |
| 7 | MNS7 | *N. stracheyi* | Soi river (Mai sapa) | LC785337 |
| 8 | MNS8 | *N. stracheyi* | Soi river (Mai sapa) | LC785336 |
| 9 | MNS9 | *N. stracheyi* | Soi river (Mai sapa) | LC785336 |
| 10 | MNS10 | *N. stracheyi* | Soi river (Mai sapa) | LC785337 |
| 11 | MNS11 | *N. stracheyi* | Soi river (Mai sapa) | LC785337 |
| 12 | MNS12 | *N. stracheyi* | Soi river (Mai sapa) | LC785337 |
| 13 | MNS13 | *N. stracheyi* | Soi river (Mai sapa) | LC785337 |
| 14 | MNS14 | *N. stracheyi* | Soi river (Mai sapa) | LC785337 |
| 15 | MNS15 | *N. stracheyi* | Soi river (Mai sapa) | LC785336 |
| 16 | MNS16 | *N. stracheyi* | Soi river (Mai sapa) | LC785337 |
| 17 | MNS17 | *N. stracheyi* | Soi river (Mai sapa) | LC785337 |
| 18 | MNS18 | *N. stracheyi* | Soi river (Mai sapa) | LC785337 |
| 19 | MNS19 | *N. stracheyi* | Soi river (Mai sapa) | LC785337 |
| 20 | MNS20 | *N. stracheyi* | Soi river (Mai sapa) | LC785337 |
| 21 | MNS21 | *N. stracheyi* | Soi river (Mai sapa) | LC785337 |
| 22 | MNS22 | *N. stracheyi* | Soi river (Mai sapa) | LC785337 |
| 23 | MNS23 | *N. stracheyi* | Soi river (Mai sapa) | LC785336 |
| 24 | MNS24 | *N. stracheyi* | Soi river (Mai sapa) | LC785337 |
| 25 | MNS25 | *N. stracheyi* | Soi river (Mai sapa) | LC785336 |
| 26 | MNS26 | *N. stracheyi* | Soi river (Mai sapa) | LC785336 |
| 27 | MNS27 | *N. stracheyi* | Soi river (Mai sapa) | LC785336 |
| 28 | MNS28 | *N. stracheyi* | Soi river (Mai sapa) | LC785337 |
| 29 | MNS29 | *N. stracheyi* | Soi river (Mai sapa) | LC785337 |
| 30 | MNS30 | *N. stracheyi* | Soi river (Mai sapa) | LC785336 |
| 31 | MNS31 | *N. stracheyi* | Soi river (Mai sapa) | LC785337 |
| 32 | MNSA1 | *N. stracheyi* | Sa-at river (Mae surin waterfall National Park) | LC785338 |
| 33 | MNSA2 | *N. stracheyi* | Sa-at river (Mae surin waterfall National Park) | LC785338 |
| 34 | MNSA3 | *N. stracheyi* | Sa-at river (Mae surin waterfall National Park) | LC785338 |
| 35 | MNSA4 | *N. stracheyi* | Sa-at river (Mae surin waterfall National Park) | LC785338 |
| 36 | MNSA5 | *N. stracheyi* | Sa-at river (Mae surin waterfall National Park) | LC785337 |
| 37 | MNSA6 | *N. stracheyi* | Sa-at river (Mae surin waterfall National Park) | LC785338 |
| 38 | MNSA7 | *N. stracheyi* | Sa-at river (Mae surin waterfall National Park) | LC785338 |
| 39 | MNSA8 | *N. stracheyi* | Sa-at river (Mae surin waterfall National Park) | LC785338 |
| 40 | MNSA9 | *N. stracheyi* | Sa-at river (Mae surin waterfall National Park) | LC785338 |
| 41 | MNSA10 | *N. stracheyi* | Sa-at river (Mae surin waterfall National Park) | LC785337 |
| 42 | MNSA11 | *N. stracheyi* | Sa-at river (Mae surin waterfall National Park) | LC785337 |
| 43 | MNSA12 | *N. stracheyi* | Sa-at river (Mae surin waterfall National Park) | LC785338 |
| 44 | MNSA13 | *N. stracheyi* | Sa-at river (Mae surin waterfall National Park) | LC785337 |
| 45 | MNSA14 | *N. stracheyi* | Sa-at river (Mae surin waterfall National Park) | LC785338 |
| 46 | MNSA15 | *N. stracheyi* | Sa-at river (Mae surin waterfall National Park) | LC785338 |
| 47 | MNSA16 | *N. stracheyi* | Sa-at river (Mae surin waterfall National Park) | LC785338 |
| 48 | MNSA17 | *N. stracheyi* | Sa-at river (Mae surin waterfall National Park) | LC785338 |
| 49 | MNSA18 | *N. stracheyi* | Sa-at river (Mae surin waterfall National Park) | LC785339 |
| 50 | MNSA19 | *N. stracheyi* | Sa-at river (Mae surin waterfall National Park) | LC785338 |
| 51 | NPJ1 | *N. stracheyi* | Tham Pa (Pa cave) | LC785337 |
| 52 | NPJ2 | *N. stracheyi* | Tham Pa (Pa cave) | LC785337 |
| 53 | NPJ3 | *N. stracheyi* | Tham Pa (Pa cave) | LC785340 |
| 54 | NPJ4 | *N. stracheyi* | Tham Pa (Pa cave) | LC785341 |
| 55 | NPJ5 | *N. stracheyi* | Tham Pa (Pa cave) | LC785337 |
| 56 | NPJ6 | *N. stracheyi* | Tham Pa (Pa cave) | LC785342 |
| 57 | NPJ7 | *N. stracheyi* | Tham Pa (Pa cave) | LC785343 |
| 58 | NPJ8 | *N. stracheyi* | Tham Pa (Pa cave) | LC785340 |
| 59 | NPJ9 | *N. stracheyi* | Tham Pa (Pa cave) | LC785342 |
| 60 | NPJ10 | *N. stracheyi* | Tham Pa (Pa cave) | LC785342 |
| 61 | NPJ11 | *N. stracheyi* | Tham Pa (Pa cave) | LC785340 |
| 62 | NPJ12 | *N. stracheyi* | Tham Pa (Pa cave) | LC785340 |
| 63 | NPJ13 | *N. stracheyi* | Tham Pa (Pa cave) | LC785340 |
| 64 | NPJ14 | *N. stracheyi* | Tham Pa (Pa cave) | LC785340 |
| 65 | NPJ15 | *N. stracheyi* | Tham Pa (Pa cave) | LC785337 |
| 66 | NPJ16 | *N. stracheyi* | Tham Pa (Pa cave) | LC785343 |
| 67 | NPJ17 | *N. stracheyi* | Tham Pa (Pa cave) | LC785337 |
| 68 | NPJ18 | *N. stracheyi* | Tham Pa (Pa cave) | LC785344 |
| 69 | NPJ19 | *N. stracheyi* | Tham Pa (Pa cave) | LC785340 |
| 70 | ThP1 | *N. stracheyi* | Tham Nam Lot (Nam Lot cave) | LC785337 |
| 71 | ThP2 | *N. stracheyi* | Tham Nam Lot (Nam Lot cave) | LC785340 |
| 72 | ThP3 | *N. stracheyi* | Tham Nam Lot (Nam Lot cave) | LC785342 |
| 73 | ThP4 | *N. stracheyi* | Tham Nam Lot (Nam Lot cave) | LC785344 |
| 74 | ThP5 | *N. stracheyi* | Tham Nam Lot (Nam Lot cave) | LC785340 |
| 75 | ThP6 | *N. stracheyi* | Tham Nam Lot (Nam Lot cave) | LC785340 |
| 76 | ThP7 | *N. stracheyi* | Tham Nam Lot (Nam Lot cave) | LC785340 |
| 77 | ThP8 | *N. stracheyi* | Tham Nam Lot (Nam Lot cave) | LC785337 |
| 78 | ThP9 | *N. stracheyi* | Tham Nam Lot (Nam Lot cave) | LC785343 |
| 79 | ThP10 | *N. stracheyi* | Tham Nam Lot (Nam Lot cave) | LC785340 |
| 80 | ThP11 | *N. stracheyi* | Tham Nam Lot (Nam Lot cave) | LC785342 |
| 81 | ThP12 | *N. stracheyi* | Tham Nam Lot (Nam Lot cave) | LC785344 |
| 82 | ThNL1 | *N. stracheyi* | Tham Nam Lot (Nam Lot cave) | LC785345 |
| 83 | ThNL2 | *N. stracheyi* | Tham Nam Lot (Nam Lot cave) | LC785345 |
| 84 | ThNL3 | *N. stracheyi* | Tham Nam Lot (Nam Lot cave) | LC785345 |
| 85 | ThNL4 | *N. stracheyi* | Tham Nam Lot (Nam Lot cave) | LC785345 |
| 86 | ThNL5 | *N. stracheyi* | Tham Nam Lot (Nam Lot cave) | LC785345 |
| 87 | ThNL6 | *N. stracheyi* | Tham Nam Lot (Nam Lot cave) | LC785345 |
| 88 | ThNL7 | *N. stracheyi* | Tham Nam Lot (Nam Lot cave) | LC785345 |
| 89 | ThNL8 | *N. stracheyi* | Tham Nam Lot (Nam Lot cave) | LC785345 |
| 90 | ThNL9 | *N. stracheyi* | Tham Nam Lot (Nam Lot cave) | LC785345 |
| 91 | ThNL10 | *N. stracheyi* | Tham Nam Lot (Nam Lot cave) | LC785345 |
| 92 | ThNL11 | *N. stracheyi* | Tham Nam Lot (Nam Lot cave) | LC785345 |
| 93 | ThNL12 | *N. stracheyi* | Tham Nam Lot (Nam Lot cave) | LC785346 |

**S8 Table**. Comparison of genetic diversity parameters between blue mahseer (*Neolissochilus stracheyi*) individuals based on 13 microsatellite loci.

|  | Population 1 | Population 2 | df | SE | *t*-test | *p*-value |
| --- | --- | --- | --- | --- | --- | --- |
| Heterozygosity (*H*_o_) | MNS ^1^ | MNSA ^2^ | 0.023 | 0.154 | 0.149 | 0.882 |
|  | MNS | NPJ ^3^ | 0.018 | 0.158 | 0.114 | 0.910 |
|  | MNS | ThP ^4^ | -0.011 | 0.156 | -0.070 | 0.944 |
|  | MNS | ThNL ^5^ | -0.003 | 0.158 | -0.019 | 0.985 |
|  | MNSA | NPJ | -0.005 | 0.160 | -0.031 | 0.975 |
|  | MNSA | ThP | -0.034 | 0.158 | -0.216 | 0.831 |
|  | MNSA | ThNL | -0.026 | 0.160 | -0.163 | 0.872 |
|  | NPJ | ThP | -0.029 | 0.162 | -0.179 | 0.859 |
|  | NPJ | ThNL | -0.021 | 0.164 | -0.128 | 0.899 |
|  | ThP | ThNL | 0.008 | 0.162 | 0.049 | 0.961 |
| Heterozygosity (*H*_e_) | MNS ^1^ | MNSA ^2^ | -0.008 | 0.115 | -0.070 | 0.945 |
|  | MNS | NPJ ^3^ | -0.028 | 0.130 | -0.216 | 0.830 |
|  | MNS | ThP ^4^ | -0.035 | 0.130 | -0.270 | 0.789 |
|  | MNS | ThNL ^5^ | 0.027 | 0.110 | 0.245 | 0.808 |
|  | MNSA | NPJ | -0.02 | 0.125 | -0.160 | 0.873 |
|  | MNSA | ThP | -0.027 | 0.125 | -0.217 | 0.830 |
|  | MNSA | ThNL | 0.035 | 0.104 | 0.336 | 0.739 |
|  | NPJ | ThP | -0.007 | 0.139 | -0.051 | 0.960 |
|  | NPJ | ThNL | 0.055 | 0.120 | 0.457 | 0.651 |
|  | ThP | ThNL | 0.062 | 0.120 | 0.515 | 0.612 |

df = Difference of means.

^1^ MNS = Mae Nam Soiy,

^2^ MNSA = Mae Nam Sa-At. ^3^ NPJ = Nong Pla Jat. ^4^ ThP = Tham Pla (Pla cave). ^5^ ThNL = Tham Nam Lot (Nam Lot cave).

**S9 Table**. Welch’s *t*-test heterozygosity (*H*_o_) and Heterozygosity (*H*_e_) of blue mahseer (*Neolissochilus stracheyi*) individuals based on 13 microsatellite loci.

| **Population** | ***H*_o_** | ***H*_e_** | **df** | **SE** | ***t*-test** | ***p*-value** |
| --- | --- | --- | --- | --- | --- | --- |
| MNS ^1^ | 0.461±0.108 | 0.443±0.085 | 0.018 | 0.137 | 0.131 | 0.896 |
| MNSA ^2^ | 0.438±0.11 | 0.451±0.077 | -0.013 | 0.134 | -0.097 | 0.923 |
| NPJ ^3^ | 0.443±0.116 | 0.471±0.098 | -0.028 | 0.152 | -0.184 | 0.855 |
| ThP ^4^ | 0.472±0.113 | 0.478±0.098 | -0.006 | 0.150 | -0.040 | 0.968 |
| ThNL ^5^ | 0.443±0.116 | 0.416±0.070 | 0.048 | 0.135 | 0.354 | 0.727 |

df = Difference of means.

^1^ MNS = Mae Nam Soiy, ^2^ MNSA = Mae Nam Sa-At. ^3^ NPJ = Nong Pla Jat. ^4^ ThP = Tham Pla (Pla cave). ^5^ ThNL = Tham Nam Lot (Nam Lot cave).

**S10 Table.** Inbreeding coefficients, relatedness, effective population size and ratio of effective population size and census population (*N*_e_*/*N) of 93 blue mahseer (*Neolissochilus stracheyi*) individuals.

| Population | N | *F*_IS_ | Relatedness (*r*) | Estimated *N*_e_ | 95% CIs for *N*_e_ | *N*_e_*/*N |
| --- | --- | --- | --- | --- | --- | --- |
| MNS ^1^ | 31 | -0.083±0.067 | -0.020±0.004 | 13.1 | 1.6-36.5 | 0.423 |
| MNSA ^2^ | 19 | -0.068±0.070 | –0.006±0.030 | 10.9 | 3.0-24.0 | 0.573 |
| NPJ ^3^ | 19 | -0.079±0.055 | –0.012±0.032 | Infinite | Infinite | - |
| ThP ^4^ | 12 | -0.090±0.075 | –0.023±0.046 | 14.3 | 0.4 - 52.8 | 1.910 |
| ThNL ^5^ | 12 | -0.044±0.139 | –0.097±0.096 | 1.2 | 0.6 - 2.0 | 0.100 |

Sample size (N); inbreeding coefficient (*F*_IS_); effective population size (*N*_e_).

^1^ MNS = Mae Nam Soiy. ^2^ MNSA = Mae Nam Sa-At. ^3^ NPJ = Nong Pla Jat. ^4^ ThP = Tham Pla (Pla cave). ^5^ ThNL = Tham Nam Lot (Nam Lot cave).

**S11 Table**. Genetic diversity of 93 blue mahseer (*Neolissochilus stracheyi*) individuals based on

13 microsatellite loci. Detailed information of all individuals.

| Population | Locus | N | *N*_a_ | *AR* | *N*_e_ | *I* | *H*_o_ | *H*_e_ | *M ratio* | *PIC* | *F* |
| --- | --- | --- | --- | --- | --- | --- | --- | --- | --- | --- | --- |
| MNS ^1^ | NY01 | 31 | 6 | 4.56 | 4.3 | 1.533 | 0.742 | 0.767 | 0.25 | 0.728 | 0.033 |
|  | NY02 | 30 | 3 | 2.944 | 2.532 | 1.004 | 0.5 | 0.605 | 0.25 | 0.531 | 0.174 |
|  | NY05 | 30 | 6 | 4.778 | 4.327 | 1.563 | 0.467 | 0.769 | 0.176 | 0.732 | 0.393 |
|  | BS04 | 31 | 5 | 3.772 | 2.114 | 1.067 | 0.516 | 0.527 | 0.208 | 0.495 | 0.021 |
|  | NY07 | 31 | 1 | 1 | 1 | 0 | 0 | 0 | 0 | 0.000 | #N/A |
|  | NY11 | 31 | 8 | 5.607 | 5.058 | 1.767 | 0.226 | 0.802 | 0.235 | 0.775 | 0.719 |
|  | NY14 | 31 | 3 | 1.807 | 1.14 | 0.284 | 0 | 0.123 | 0.3 | 0.119 | 1 |
|  | NY06 | 31 | 6 | 3.849 | 2.043 | 1.075 | 0.613 | 0.51 | 0.273 | 0.482 | -0.201 |
|  | NY12 | 27 | 2 | 2 | 2 | 0.693 | 1 | 0.5 | 0.077 | 0.375 | -1 |
|  | NY09 | 27 | 3 | 2.991 | 2.876 | 1.077 | 0.926 | 0.652 | 0.214 | 0.579 | -0.42 |
|  | BS03 | 31 | 2 | 2 | 2 | 0.693 | 1 | 0.5 | 0.25 | 0.375 | -1 |
|  | NY13 | 31 | 1 | 1 | 1 | 0 | 0 | 0 | 0 | 0.000 | #N/A |
|  | BS05 | 31 | 1 | 1 | 1 | 0 | 0 | 0 | 0 | 0.000 | #N/A |
|  | Mean | 31 | 3.615 | 2.87 | 2.415 | 0.827 | 0.461 | 0.443 | 0.174 | 0.399 | -0.028 |
|  | SE | 0.411 | 0.646 | 1.491 | 0.381 | 0.17 | 0.108 | 0.085 | 0.068 | 0.286 | 0.183 |
| MNSA ^2^ | NY01 | 19 | 5 | 3.884 | 2.579 | 1.176 | 0.632 | 0.612 | 0.227 | 0.560 | -0.032 |
|  | NY02 | 17 | 2 | 2 | 2 | 0.693 | 0.647 | 0.5 | 0.25 | 0.375 | -0.294 |
|  | NY05 | 18 | 4 | 3.713 | 2.919 | 1.198 | 0.222 | 0.657 | 0.4 | 0.599 | 0.662 |
|  | BS04 | 19 | 8 | 5.244 | 3.438 | 1.554 | 0.632 | 0.709 | 0.211 | 0.677 | 0.109 |
|  | NY07 | 19 | 1 | 1 | 1 | 0 | 0 | 0 | 0 | 0.000 | #N/A |
|  | NY11 | 19 | 7 | 4.644 | 2.063 | 1.189 | 0 | 0.515 | 0.259 | 0.499 | 1 |
|  | NY14 | 19 | 4 | 2.961 | 1.81 | 0.822 | 0.053 | 0.447 | 0.4 | 0.399 | 0.882 |
|  | NY06 | 19 | 7 | 5.928 | 5.597 | 1.807 | 0.789 | 0.821 | 0.292 | 0.797 | 0.039 |
|  | NY12 | 19 | 2 | 2 | 2 | 0.693 | 1 | 0.5 | 0.077 | 0.375 | -1 |
|  | NY09 | 17 | 4 | 3.39 | 2.639 | 1.106 | 0.941 | 0.621 | 0.333 | 0.561 | -0.515 |
|  | BS03 | 18 | 2 | 2 | 1.906 | 0.668 | 0.778 | 0.475 | 0.25 | 0.362 | -0.636 |
|  | NY13 | 19 | 1 | 1 | 1 | 0 | 0 | 0 | 0 | 0.000 | #N/A |
|  | BS05 | 19 | 1 | 1 | 1 | 0 | 0 | 0 | 0 | 0.000 | #N/A |
|  | Mean | 19 | 3.692 | 2.982 | 2.304 | 0.839 | 0.438 | 0.451 | 0.2 | 0.400 | 0.022 |
|  | SE | 0.215 | 0.683 | 1.587 | 0.344 | 0.162 | 0.11 | 0.077 | 0.066 | 0.261 | 0.185 |
| NPJ ^3^ | NY01 | 19 | 7 | 5.361 | 4.457 | 1.655 | 0.842 | 0.776 | 0.318 | 0.743 | -0.086 |
|  | NY02 | 17 | 5 | 4.014 | 2.89 | 1.251 | 0.647 | 0.654 | 0.357 | 0.602 | 0.011 |
|  | NY05 | 19 | 7 | 5.773 | 5.348 | 1.774 | 0.632 | 0.813 | 0.292 | 0.787 | 0.223 |
|  | BS04 | 19 | 13 | 8.387 | 8.022 | 2.316 | 0.684 | 0.875 | 0.325 | 0.864 | 0.218 |
|  | NY07 | 19 | 1 | 1 | 1 | 0 | 0 | 0 | 0 | 0.000 | #N/A |
|  | NY11 | 19 | 8 | 6.528 | 6.119 | 1.928 | 0 | 0.837 | 0.242 | 0.817 | 1 |
|  | NY14 | 19 | 3 | 2.215 | 1.241 | 0.409 | 0 | 0.194 | 0.3 | 0.185 | 1 |
|  | NY06 | 19 | 7 | 5.426 | 4.846 | 1.696 | 0.789 | 0.794 | 0.269 | 0.763 | 0.005 |
|  | NY12 | 19 | 2 | 2 | 2 | 0.693 | 1 | 0.5 | 0.077 | 0.375 | -1 |
|  | NY09 | 18 | 3 | 2.389 | 2.111 | 0.8 | 1 | 0.526 | 0.25 | 0.414 | -0.9 |
|  | BS03 | 18 | 2 | 1.784 | 1.18 | 0.287 | 0.167 | 0.153 | 0.25 | 0.141 | -0.091 |
|  | NY13 | 18 | 1 | 1 | 1 | 0 | 0 | 0 | 0 | 0.000 | #N/A |
|  | BS05 | 18 | 1 | 1 | 1 | 0 | 0 | 0 | 0 | 0.000 | #N/A |
|  | Mean | 9 | 4.615 | 3.606 | 3.17 | 0.985 | 0.443 | 0.471 | 0.215 | 0.438 | 0.038 |
|  | SE | 0.183 | 1.003 | 2.359 | 0.65 | 0.229 | 0.116 | 0.098 | 0.096 | 0.342 | 0.183 |
| ThP ^4^ | NY01 | 12 | 6 | 5.087 | 4.114 | 1.549 | 0.917 | 0.757 | 0.3 | 0.718 | -0.211 |
|  | NY02 | 12 | 4 | 3.776 | 2.642 | 1.146 | 0.583 | 0.622 | 0.286 | 0.569 | 0.061 |
|  | NY05 | 11 | 6 | 5.552 | 5.042 | 1.685 | 0.636 | 0.802 | 0.429 | 0.772 | 0.206 |
|  | BS04 | 12 | 10 | 7.653 | 6.698 | 2.073 | 0.75 | 0.851 | 0.278 | 0.834 | 0.118 |
|  | NY07 | 12 | 1 | 1 | 1 | 0 | 0 | 0 | 0 | 0.000 | #N/A |
|  | NY11 | 12 | 6 | 5.375 | 4.571 | 1.63 | 0.083 | 0.781 | 0.375 | 0.749 | 0.893 |
|  | NY14 | 12 | 1 | 1 | 1 | 0 | 0 | 0 | 0 | 0.000 | #N/A |
|  | NY06 | 12 | 9 | 7.359 | 6.857 | 2.034 | 0.417 | 0.854 | 0.214 | 0.837 | 0.512 |
|  | NY12 | 12 | 3 | 2.583 | 2.165 | 0.837 | 1 | 0.538 | 0.107 | 0.432 | -0.858 |
|  | NY09 | 12 | 3 | 2.58 | 2.165 | 0.837 | 1 | 0.538 | 0.25 | 0.432 | -0.858 |
|  | BS03 | 12 | 2 | 2 | 1.882 | 0.662 | 0.75 | 0.469 | 0.25 | 0.359 | -0.6 |
|  | NY13 | 12 | 1 | 1 | 1 | 0 | 0 | 0 | 0 | 0.000 | #N/A |
|  | BS05 | 12 | 1 | 1 | 1 | 0 | 0 | 0 | 0 | 0.000 | #N/A |
|  | Mean | 12 | 4.077 | 3.615 | 3.088 | 0.958 | 0.472 | 0.478 | 0.193 | 0.439 | 0.499 |
|  | SE | 0.077 | 0.858 | 2.422 | 0.594 | 0.22 | 0.113 | 0.098 | 0.074 | 0.341 | 0.102 |
| ThNL ^5^ | NY01 | 12 | 3 | 2.941 | 2.462 | 0.974 | 1 | 0.594 | 0.3 | 0.511 | -0.684 |
|  | NY02 | 10 | 3 | 2.997 | 2.469 | 0.997 | 0.7 | 0.595 | 0.375 | 0.528 | -0.176 |
|  | NY05 | 12 | 4 | 3.875 | 2.717 | 1.179 | 0.333 | 0.632 | 0.333 | 0.585 | 0.473 |
|  | BS04 | 11 | 4 | 3.515 | 2.602 | 1.083 | 0.909 | 0.616 | 0.154 | 0.539 | -0.477 |
|  | NY07 | 12 | 2 | 1.837 | 1.18 | 0.287 | 0 | 0.153 | 2 | 0.141 | 1 |
|  | NY11 | 12 | 6 | 5.088 | 3.165 | 1.427 | 0.083 | 0.684 | 0.353 | 0.649 | 0.878 |
|  | NY14 | 12 | 2 | 1.837 | 1.18 | 0.287 | 0 | 0.153 | 0.286 | 0.141 | 1 |
|  | NY06 | 12 | 4 | 2.75 | 1.297 | 0.514 | 0.25 | 0.229 | 0.154 | 0.221 | -0.091 |
|  | NY12 | 12 | 3 | 2.837 | 2.341 | 0.922 | 0.917 | 0.573 | 0.115 | 0.479 | -0.6 |
|  | NY09 | 11 | 4 | 3.6 | 2.689 | 1.123 | 1 | 0.628 | 0.2 | 0.563 | -0.592 |
|  | BS03 | 12 | 2 | 2 | 1.882 | 0.662 | 0.75 | 0.469 | 0.25 | 0.359 | -0.6 |
|  | NY13 | 7 | 1 | 1 | 1 | 0 | 0 | 0 | 0 | 0.000 | #N/A |
|  | BS05 | 12 | 2 | 1.583 | 1.087 | 0.173 | 0.083 | 0.08 | 2 | 0.077 | -0.043 |
|  | Mean | 11.308 | 3.077 | 2.758 | 2.006 | 0.741 | 0.464 | 0.416 | 0.228 | 0.369 | 0.007 |
|  | SE | 0.398 | 0.366 | 1.067 | 0.211 | 0.125 | 0.116 | 0.07 | 0.039 | 0.223 | 0.183 |

Sample size (N); number of alleles (*N_a_*); allelic richness (*AR*); number of effective alleles (*N_e_*); Shannon’s information index (*I*); observed heterozygosity (*H_o_*); expected heterozygosity (*H_e_*); polymorphic information content (PIC); fixation index (*F*).

^1^ MNS = Mae Nam Soiy. ^2^ MNSA = Mae Nam Sa-At. ^3^ NPJ = Nong Pla Jat. ^4^ ThP = Tham Pla (Pla cave). ^5^ ThNL = Tham Nam Lot (Nam Lot cave).

**S12 Table**. Probability of identity estimated of blue mahseer (*Neolissochilus stracheyi*) individuals based on 13 microsatellite loci.

| Locus | Unbias/loc. | Prod(unbias) |
| --- | --- | --- |
| NY01 | 4.62 × 10^-2^ | 4.62 × 10^-2^ |
| NY02 | 1.63 × 10^-1^ | 7.51 × 10^-3^ |
| NY05 | 4.25 × 10^-2^ | 3.19 × 10^-3^ |
| BS04 | 2.16 × 10^-2^ | 6.89 × 10^-6^ |
| NY07 | 9.57 × 10^-1^ | 6.59 × 10^-6^ |
| NY11 | 2.41 × 10^-2^ | 1.59 × 10^-7^ |
| NY14 | 6.23 × 10^-1^ | 9.91 × 10^-8^ |
| NY06 | 3.84 × 10^-2^ | 3.80 × 10^-9^ |
| NY12 | 3.48 × 10^-1^ | 1.32 × 10^-9^ |
| NY09 | 1.44 × 10^-1^ | 1.90 × 10^-10^ |
| BS03 | 3.92 × 10^-1^ | 7.45 × 10^-11^ |
| NY13 | 1.00 × 10 | 7.45 × 10^-11^ |
| BS05 | 9.78 × 10^-1^ | 7.29 × 10^-11^ |
| Mean | 3.7 × 10^-1^ | 4.2 × 10^-3^ |
| S.D. | 3.8 × 10^-1^ | 1.2 × 10^-2^ |

**S13 Table**. Distributions of *r* values and *F*_IS_ values for the blue mahseer (*Neolissochilus stracheyi*).

| **Population 1** | **Population 2** | **Relatedness (*r*)** | | **Inbreeding coefficient (*F*_IS_)** | |
| --- | --- | --- | --- | --- | --- |
|  |  | **Density** | ***p-*value** | **Density** | ***p-*value** |
| All populations | MNS^1^ | 0.236 | <0.05 | 0.118 | 0.873 |
| All populations | MNSA ^2^ | 0.260 | <0.05 | 0.180 | 0.595 |
| All populations | NPJ ^3^ | 0.270 | <0.05 | 0.175 | 0.631 |
| All populations | ThP ^4^ | 0.457 | <0.05 | 0.328 | 0.158 |
| All populations | ThNL ^5^ | 0.355 | <0.05 | 0.253 | 0.431 |
| MNS | MNSA | 0.099 | 0.168 | 0.250 | 0.381 |
| MNS | NPJ | 0.103 | 0.144 | 0.261 | 0.325 |
| MNS | ThP | 0.271 | 0.000 | 0.382 | 0.122 |
| MNS | ThNL | 0.180 | 0.047 | 0.274 | 0.438 |
| MNSA | NPJ | 0.094 | 0.443 | 0.211 | 0.808 |
| MNSA | ThP | 0.245 | 0.007 | 0.465 | 0.057 |
| MNSA | ThNL | 0.169 | 0.134 | 0.250 | 0.649 |
| NPJ | ThP | 0.211 | 0.028 | 0.307 | 0.409 |
| NPJ | ThNL | 0.233 | 0.011 | 0.289 | 0.459 |
| ThP | ThNL | 0.182 | 0.216 | 0.417 | 0.256 |

^1^ MNS = Mae Nam Soiy. ^2^ MNSA = Mae Nam Sa-At. ^3^ NPJ = Nong Pla Jat. ^4^ ThP = Tham Pla (Pla cave). ^5^ ThNL = Tham Nam Lot (Nam Lot cave)

**S14 Table**. Pairwise population Nei’s genetic distance (*D*) values of 93 blue mahseer (*Neolissochilus stracheyi*) individuals based on 13 microsatellite loci.

| Nei *D* | MNS | MNSA | NPJ | ThP | ThNL |
| --- | --- | --- | --- | --- | --- |
| MNS ^1^ | 0.000 |  |  |  |  |
| MNSA ^2^ | 0.143 | 0.000 |  |  |  |
| NPJ ^3^ | 0.145 | 0.108 | 0.000 |  |  |
| ThP ^4^ | 0.161 | 0.111 | 0.047 | 0.000 |  |
| ThNL ^5^ | 0.348 | 0.339 | 0.310 | 0.291 | 0.000 |

^1^ MNS = Mae Nam Soiy. ^2^ MNSA = Mae Nam Sa-At. ^3^ NPJ = Nong Pla Jat. ^4^ ThP = Tham Pla (Pla cave). ^5^ ThNL = Tham Nam Lot (Nam Lot cave).

**S15 Table**. Analysis of molecular variance (AMOVA) results for blue mahseer (*Neolissochilus stracheyi*) individuals based on 13 microsatellite loci.

| Source of variation | df | Sum of squares | Variance components | Percentage of variation |
| --- | --- | --- | --- | --- |
| Among populations | 4 | 69.420 | 0.412 | 14.94 % |
| Among individuals within populations | 88 | 225.188 | 0.212 | 7.690 % |
| Within individuals | 93 | 198.500 | 2.134 | 77.370 % |
| Total | 185 | 493.108 | 2.758 |  |

**S16 Table**. Pairwise genetic differentiation (*F_ST_*), pairwise *F*_ST_^ENA^ values with ENA correction for null alleles and *R*_ST_ values of blue mahseer (*Neolissochilus stracheyi*) based on 13 microsatellite loci.

| Combination | *F*_ST_ | *F*_ST_^ENA^ | *R*_ST_ |
| --- | --- | --- | --- |
| MNS ^1^ x MNSA ^2^ | 0.137* | 0.112 | 0.117 |
| MNS x NPJ ^3^ | 0.131* | 0.118 | 0.126 |
| MNS x ThP ^4^ | 0.141* | 0.122 | 0.231 |
| MNS x ThNL ^5^ | 0.262* | 0.254 | 0.368 |
| MNSA ^2^ x NPJ ^3^ | 0.090* | 0.070 | 0.259 |
| MNSA ^2^ x ThP ^4^ | 0.089* | 0.073 | 0.201 |
| MNSA ^2^ x ThNL ^5^ | 0.247* | 0.235 | 0.393 |
| NPJ ^3^ x ThP ^4^ | 0.024* | 0.016 | 0.069 |
| NPJ ^3^ x ThNL ^5^ | 0.209* | 0.219 | 0.504 |
| ThP ^4^ x ThNL ^5^ | 0.192* | 0.203 | 0.556 |

* *p*-value < 0.05

^1^ MNS = Mae Nam Soiy. ^2^ MNSA = Mae Nam Sa-At. ^3^ NPJ = Nong Pla Jat. ^4^ ThP = Tham Pla (Pla cave). ^5^ ThNL = Tham Nam Lot (Nam Lot cave).

**S17 Table**. Wilcoxon sign rank test to evaluate 93 blue mahseer (*Neolissochilus stracheyi*) for mutation drift equilibrium under different models.

| **Locality** | **Wilcoxon test** | | **Mode-shift test** |
| --- | --- | --- | --- |
|  | **T.P.M.** | **S.M.M** |  |
|  | **Probability for one tail test for *H* excess** | **Probability for one tail test for *H* excess** |  |
| MNS ^1^ | 0.423 | 0.500 | normal L-shaped distribution |
| MNSA^2^ | 0.577 | 0.615 | normal L-shaped distribution |
| NPJ ^3^ | 0.278 | 0.422 | normal L-shaped distribution |
| ThP ^4^ | 0.003 | 0.005 | normal L-shaped distribution |
| ThNL ^5^ | 0.765 | 0.809 | normal L-shaped distribution |

^1^ MNS = Mae Nam Soiy. ^2^ MNSA = Mae Nam Sa-At. ^3^ NPJ = Nong Pla Jat. ^4^ ThP = Tham Pla (Pla cave). ^5^ ThNL = Tham Nam Lot (Nam Lot cave).

**S18 Table**. All source/recipient population comparisons contain the mean migration rates and 95% confidence intervals determined by BAYESASS using the microsatellite data.

| **Migration Rates** | **Posterior mean of migration rates** | **Standard deviation** |
| --- | --- | --- |
| MNS->MNS | 0.962 | 0.017 |
| MNS->MNSA | 0.010 | 0.010 |
| MNS->NPJ | 0.010 | 0.010 |
| MNS->ThP | 0.009 | 0.009 |
| MNS->ThNL | 0.009 | 0.009 |
| MNSA->MNS | 0.017 | 0.016 |
| MNSA->MNSA | 0.868 | 0.114 |
| MNSA->NPJ | 0.087 | 0.113 |
| MNSA->ThP | 0.014 | 0.013 |
| MNSA->ThNL | 0.014 | 0.013 |
| NPJ->MNS | 0.014 | 0.013 |
| NPJ->MNSA | 0.021 | 0.020 |
| NPJ->NPJ | 0.938 | 0.028 |
| NPJ->ThP | 0.013 | 0.013 |
| NPJ->ThNL | 0.013 | 0.013 |
| ThP->MNS | 0.020 | 0.019 |
| ThP->MNSA | 0.021 | 0.020 |
| ThP->NPJ | 0.234 | 0.036 |
| ThP->ThP | 0.686 | 0.019 |
| ThP->ThNL | 0.039 | 0.025 |
| ThNL->MNS | 0.021 | 0.019 |
| ThNL->MNSA | 0.021 | 0.020 |
| ThNL->NPJ | 0.021 | 0.020 |
| ThNL->ThP | 0.023 | 0.022 |
| ThNL->ThNL | 0.914 | 0.036 |

^1^ MNS = Mae Nam Soiy. ^2^ MNSA = Mae Nam Sa-At. ^3^ NPJ = Nong Pla Jat. ^4^ ThP = Tham Pla (Pla cave). ^5^ ThNL = Tham Nam Lot (Nam Lot cave).

**S19 Table**. Bayesian estimates of mutation-scaled effective population sizes (Θ) and asymmetric migration rates (*M*) among blue mahseer (*Neolissochilus stracheyi*) for the 13 microsatellite loci.

| **Locus** | **Parameter** | **2.50%** | **25.00%** | **Mode** | **75.00%** | **97.50%** |
| --- | --- | --- | --- | --- | --- | --- |
| All | ΘMNS | 0.001 | 0.003 | 0.004 | 0.005 | 0.007 |
| All | ΘMNSA | 0.073 | 0.096 | 0.099 | 0.100 | 0.100 |
| All | ΘNPJ | 0.068 | 0.094 | 0.096 | 0.098 | 0.100 |
| All | ΘThP | 0.065 | 0.090 | 0.093 | 0.096 | 0.098 |
| All | ΘThNL | 0.001 | 0.002 | 0.003 | 0.004 | 0.005 |
| All | M_MNSA->MNS_ | 4.000 | 20.000 | 30.333 | 39.333 | 70.667 |
| All | M_NPJ->MNS_ | 12.667 | 31.333 | 43.000 | 54.667 | 99.333 |
| All | M_ThP->MNS_ | 0.000 | 6.000 | 13.667 | 21.333 | 38.667 |
| All | M_ThNL->MNS_ | 0.000 | 9.333 | 17.667 | 25.333 | 36.000 |
| All | M_MNS->MNSA_ | 10.000 | 32.667 | 43.000 | 54.667 | 87.333 |
| All | M_NPJ->MNSA_ | 125.333 | 240.000 | 259.667 | 284.667 | 310.000 |
| All | M_ThP->MNSA_ | 24.667 | 45.333 | 57.667 | 70.000 | 96.000 |
| All | M_ThNL->MNSA_ | 0.000 | 0.000 | 7.667 | 24.000 | 90.000 |
| All | M_MNS->NPJ_ | 37.333 | 50.000 | 65.667 | 82.667 | 106.000 |
| All | M_MNSA->NPJ_ | 820.000 | 956.000 | 983.667 | 1000.000 | 1000.000 |
| All | M_ThP->NPJ_ | 247.333 | 278.000 | 325.000 | 361.333 | 529.333 |
| All | M_ThNL->NPJ_ | 5.333 | 22.000 | 33.000 | 43.333 | 66.000 |
| All | M_MNS->ThP_ | 0.000 | 1.333 | 13.667 | 26.000 | 36.667 |
| All | M_MNSA->ThP_ | 184.667 | 206.000 | 223.000 | 242.000 | 303.333 |
| All | M_NPJ->ThP_ | 103.333 | 131.333 | 156.333 | 176.667 | 183.333 |
| All | M_ThNL->ThP_ | 83.333 | 107.333 | 118.333 | 128.000 | 147.333 |
| All | M_MNS->ThNL_ | 26.667 | 132.667 | 147.000 | 164.000 | 224.667 |
| All | M_MNSA->ThNL_ | 93.333 | 125.333 | 138.333 | 150.667 | 175.333 |
| All | M_NPJ->ThNL_ | 151.333 | 172.000 | 200.333 | 240.000 | 278.667 |
| All | M_ThP->ThNL_ | 0.000 | 0.000 | 1.667 | 10.667 | 26.000 |

^1^ MNS = Mae Nam Soiy. ^2^ MNSA = Mae Nam Sa-At. ^3^ NPJ = Nong Pla Jat. ^4^ ThP = Tham Pla (Pla cave). ^5^ ThNL = Tham Nam Lot (Nam Lot cave).

**S20 Table**. The effective number of immigrants (*N*_m_) from population *i* into population *j* per generation.

| *j*  *i* | MNS^1^ | MNSA^2^ | NPJ^3^ | ThP^4^ | ThNL^5^ |
| --- | --- | --- | --- | --- | --- |
| MNS^1^ |  | 43.000 | 65.667 | 13.667 | 147.000 |
| MNSA^2^ | 30.333 |  | 983.667 | 223.000 | 138.333 |
| NPJ^3^ | 43.000 | 259.667 |  | 156.333 | 200.333 |
| ThP^4^ | 13.667 | 57.667 | 325.000 |  | 1.667 |
| ThNl^5^ | 17.667 | 7.667 | 33.000 | 118.333 |  |

^1^ MNS = Mae Nam Soiy. ^2^ MNSA = Mae Nam Sa-At. ^3^ NPJ = Nong Pla Jat. ^4^ ThP = Tham Pla (Pla cave). ^5^ ThNL = Tham Nam Lot (Nam Lot cave).

**S21 Table.** Genetic differentiation between the five populations of blue mahseer (*Neolissochilus stracheyi*) for D-loop.

| **Population 1** | **Population 2** | ***G*_ST_** | ***Ф*_ST_** | ***F*_ST_** | ***D*_xy_** | ***D*_a_** | ***N*_m_** |
| --- | --- | --- | --- | --- | --- | --- | --- |
| MNS ^1^ | MNSA ^2^ | 0.292 | 0.175 | 0.280^*^ | 0.017 | 0.005 | 1.284 |
| MNS | NPJ ^3^ | 0.139 | 0.104 | 0.162^*^ | 0.018 | 0.003 | 2.588 |
| MNS | ThP ^4^ | 0.153 | 0.088 | 0.147^*^ | 0.019 | 0.002 | 2.906 |
| MNS | ThNL ^5^ | 0.405 | 0.451 | 0.654^*^ | 0.027 | 0.020 | 0.265 |
| MNSA | NPJ | 0.219 | 0.303 | 0.435^*^ | 0.024 | 0.010 | 0.650 |
| MNSA | ThP | 0.223 | 0.259 | 0.383^*^ | 0.024 | 0.009 | 0.806 |
| MNSA | ThNL | 0.502 | 0.498 | 0.653^*^ | 0.019 | 0.014 | 0.265 |
| NPJ | ThP | -0.023 | 0.007 | -0.056^ns^ | 0.017 | -0.001 | infinite |
| NPJ | ThNL | 0.317 | 0.551 | 0.701^*^ | 0.033 | 0.025 | 0.213 |
| ThP | ThNL | 0.347 | 0.554 | 0.687^*^ | 0.033 | 0.022 | 0.228 |

**S22 Table**. Neutrality tests of mitochondrial D-loop sequence of blue mahseer (*Neolissochilus stracheyi*) population.

| **Population** | **Tajima** | **Fu and Li’s *D**** | **Fu and Li’s *F**** | **Fu's *F*_s_** | **Ewens-Watterson test** | **Chakraborty’s test** | **Ramos-Onsins and Rozas’s R_2_** | **Raggedness index** |
| --- | --- | --- | --- | --- | --- | --- | --- | --- |
| MNS^1^ | 3.235^***^ | 1.566^**^ | 2.461^**^ | 17.470^ns^ | 0.123^ns^ | 0.922 | 0.245 | 0.741* |
| MNSA^2^ | 1.372^ns^ | 1.214^ns^ | 1.460^ns^ | 9.004^ns^ | 0.668^ns^ | 0.498 | 0.192 | 0.471** |
| NPJ^3^ | 1.207^ns^ | 1.618^**^ | 1.739^*^ | 7.350^ns^ | 0.187^ns^ | 0.821 | 0.187 | 0.103** |
| ThP^4^ | 1.554^ns^ | 1.403^*^ | 1.643^*^ | 5.235^ns^ | 0.369^ns^ | 0.723 | 0.220 | 0.091 |
| ThNl^5^ | -1.747^ns^ | -2.113^ns^ | -2.285^ns^ | 1.559^ns^ | 1.000^ns^ | 0.353 | 0.276 | 0.750 |
| All population | 1.799^ns^ | 0.922^ns^ | 1.518^ns^ | 13.198^ns^ | 0.469^ns^ | 0.664 | 0.158 | 0.055 |

ns = nonsignificant, * *p*-value < 0.05, ** *p*-value < 0.01, *** *p*-value < 0.001

^1^ MNS = Mae Nam Soiy. ^2^ MNSA = Mae Nam Sa-At. ^3^ NPJ = Nong Pla Jat. ^4^ ThP = Tham Pla (Pla cave). ^5^ ThNL = Tham Nam Lot (Nam Lot cave).

**S23 Table**. Mutation-scaled effective population sizes (Θ) in 5 populations of the blue mahseer (*Neolissochilus stracheyi*) and asymmetric migration rates (*M*) between populations estimated with the mitochondrial DNA D-loop sequences.

| **Parameter** | **2.50%** | **25.00%** | **Mode** | **75.00%** | **97.50%** |
| --- | --- | --- | --- | --- | --- |
| Θ_MNS_ | 0.096 | 0.098 | 0.099 | 0.099 | 0.100 |
| Θ_MNSA_ | 0.095 | 0.097 | 0.098 | 0.099 | 0.100 |
| Θ_NPJ_ | 0.081 | 0.094 | 0.098 | 0.099 | 0.100 |
| Θ_ThP_ | 0.095 | 0.098 | 0.099 | 0.099 | 0.100 |
| Θ_ThNL_ | 0.093 | 0.097 | 0.098 | 0.100 | 0.100 |
| M_MNSA->MNS_ | 0.000 | 4.000 | 10.300 | 16.700 | 26.000 |
| M_NPJ->MNS_ | 10.000 | 20.700 | 29.000 | 36.000 | 46.700 |
| M_ThP->MNS_ | 0.000 | 7.300 | 15.000 | 22.000 | 30.700 |
| M_ThNL->MNS_ | 0.000 | 0.700 | 7.000 | 12.000 | 22.700 |
| M_MNS->MNSA_ | 0.000 | 0.000 | 4.300 | 9.300 | 20.700 |
| M_NPJ->MNSA_ | 42.000 | 54.000 | 62.300 | 70.000 | 82.000 |
| M_ThP->MNSA_ | 64.000 | 76.700 | 85.700 | 93.300 | 106.000 |
| M_ThNL->MNSA_ | 58.000 | 70.700 | 79.700 | 87.300 | 100.000 |
| M_MNS->NPJ_ | 59.300 | 74.000 | 84.300 | 92.700 | 108.700 |
| M_MNSA->NPJ_ | 18.000 | 30.700 | 39.700 | 47.300 | 60.700 |
| M_ThP->NPJ_ | 202.700 | 224.700 | 236.300 | 248.700 | 271.300 |
| M_ThNL->NPJ_ | 99.300 | 116.700 | 127.700 | 136.700 | 154.000 |
| M_MNS->ThP_ | 0.000 | 5.300 | 12.300 | 18.700 | 28.000 |
| M_MNSA->ThP_ | 64.000 | 77.300 | 85.700 | 94.000 | 106.700 |
| M_NPJ->ThP_ | 0.000 | 0.000 | 5.700 | 10.700 | 22.000 |
| M_ThNL->ThP_ | 107.300 | 122.000 | 131.000 | 139.300 | 154.000 |
| M_MNS->ThNL_ | 0.000 | 0.700 | 6.300 | 12.000 | 22.700 |
| M_MNSA->ThNL_ | 119.300 | 135.300 | 145.000 | 154.000 | 170.000 |
| M_NPJ->ThNL_ | 112.000 | 130.700 | 141.700 | 152.000 | 170.700 |
| M_ThP->ThNL_ | 43.300 | 56.700 | 65.000 | 73.300 | 86.000 |

**S24 Table**. The effective number of immigrants (*N*_m_) from population *i* into population *j* per generation in five populations blue mahseer (*Neolissochilus stracheyi*) for the mitochondrial DNA D-loop sequences.

| ***_i_*  *^j^*** | **MNS** | **MNSA** | **NPJ** | **ThP** | **ThNL** |
| --- | --- | --- | --- | --- | --- |
| MNS |  | 0.106 | 2.067 | 0.303 | 0.155 |
| MNSA | 0.254 |  | 0.974 | 2.110 | 3.568 |
| NPJ | 0.715 | 1.531 |  | 0.140 | 3.487 |
| ThP | 0.370 | 2.106 | 5.795 |  | 1.599 |
| ThNL | 0.173 | 1.959 | 3.132 | 3.226 |  |
